# MicroRNAs and targets in senescent litchi fruit during ambient storage and post-cold storage shelf life

**DOI:** 10.1186/s12870-015-0509-2

**Published:** 2015-07-16

**Authors:** Furong Yao, Hong Zhu, Chun Yi, Hongxia Qu, Yueming Jiang

**Affiliations:** Key Laboratory of Plant Resources Conservation and Sustainable Utilization, South China Botanical Garden, Chinese Academy of Sciences, Guangzhou, 510650 P R of China; University of Chinese Academy of Sciences, Beijing, 100049 P R of China

**Keywords:** *Litchi chinensis*, MicroRNAs, Targets, Senescence, Storage

## Abstract

**Background:**

Litchi has a high commercial value due to its bright color and rich nutrients. However, it deteriorates with the pericarp turning brown within 1–2 days after harvest. The factors that mediate litchi fruit senescence are complicated. MicroRNAs act as negative regulators involved in almost every physiological process. To understand the mechanism of litchi fruit senescence and pericarp browning at the miRNA level, five small RNA libraries and a degradome library prepared from the pericarp of litchi fruit subjected to ambient storage and post-cold storage shelf life were sequenced.

**Results:**

By aligning the sRNA reads onto the litchi unigene assembly, 296 miRNAs belonging to 49 known miRNA families were first identified from litchi. In addition, 11 litchi-specific miRNAs were identified. Among these, 167 known miRNAs were identified to cleave 197 targets, and three litchi-specific miRNAs were found to have five targets. Through combined analysis of stem-loop quantitative real-time polymerase chain reaction (qRT-PCR) and transcriptome profiling, 14 miRNA-target pairs were found to be actively involved in litchi fruit senescence-related processes, including energy regulation, anthocyanin metabolism, hormone signaling, and pathogen-infection defense.

**Conclusions:**

A network of miRNA-targets that regulate litchi fruit senescence has been proposed, revealing the miRNA-mediated regulation in senescent litchi fruit. This will aid in developing new strategies to postpone the senescence of litchi fruit and other horticultural products.

**Electronic supplementary material:**

The online version of this article (doi:10.1186/s12870-015-0509-2) contains supplementary material, which is available to authorized users.

## Background

Litchi (*Litchi chinensis* Sonn*.*), a native fruit of China, has a high commercial value in the international market due to its bright color and the presence of rich nutrients in the pulp [1]. However, this perishable fruit senesces quickly after harvest, which is reflected in the browning of the pericarp within 1–2 days at ambient temperature [2]. Fruit senescence is controlled by multiple internal and environmental factors, each of which upregulates a subset of senescence-associated genes (*SAG*s), which are sequentially involved in perception, signal transduction pathways, and downstream responses; all of these genes are subject to complex regulatory crosstalk [3, 4]. It has been reported that cellular energy status plays a vital role in regulating litchi fruit senescence and browning [5]. As an energy regulator, snf1-related kinase 1 (SnRK1) has been reported to be involved in the global regulation of carbon and nitrogen metabolism, further affecting the cellular energy level [6, 7]. Moreover, pathogen infection usually induces fruit senescence and browning. Plants have their own mechanism to defend against pathogen attack, among which the accumulation of callose is important [8]. Transcription factors (TFs) usually regulate a wide range of biological processes, including senescence [9, 10]. For example, MYBs play an important role in fruit senescence [11]; in particular, they have been reported to be widely involved in the regulation of anthocyanin metabolism [12–15], which is an important factor affecting pericarp browning [16, 17].

MicroRNAs (miRNAs) are endogenous noncoding small RNAs that have been found in plants, animals, and even viruses [18–20]. They play important roles in plant development through the regulation of gene expression by mRNA degradation or translational inhibition [18, 21]. In plants, cleavage occurs in the middle of the mRNA–miRNA duplex (between the tenth and eleventh nucleotides from 5′ end of the miRNA) [22]. As a result, the 3′ fragment of the target mRNA has a monophosphate at its 5′ end, which is an important factor that validates a miRNA target [23]. In recent years, with the rapid development of high throughput sequencing, many miRNAs and their target genes have been identified from diverse plant species [24]. The discovery of these miRNAs and their targets makes it possible to further understand the gene regulation network and biological mechanism mediated by miRNAs. However, little is known about miRNAs in litchi.

Previous studies have demonstrated that miRNAs can mediate plant senescence. For example, miR164 has been found to regulate the NAC transcription factor *ORE1*, and senescence is accelerated in the miR164 mutant [25]. In another case, miR390 triggers the production of trans-acting siRNA from *TAS3*, leading to mRNA degradation of the auxin response factors ARF2/3/4 and ultimately affecting the timing of senescence [26]. Additionally, the overexpression of miR319 targeting TCP transcription factors has been reported to result in a stay-green phenotype [27, 28]. With regard to fruit, only a few miRNAs (miR156 and miR172) have been verified to fine-tune the expression of *CNR* and *AP2a*, which are important regulators in tomato fruit ripening [29, 30]. However, little is known about miRNA expression and its targets in stored fruit senescence in general. Thus, our study has a broader appeal to stored fruits other than litchi alone.

To understand the regulation of miRNAs on the senescence of harvested litchi fruit, five small RNA libraries of litchi fruit after storage under different temperatures and a mixed degradome library were sequenced. MiRNAs and their targets were identified using a litchi unigene reference assembled from transcriptome sequencing. A number of miRNAs were then validated by stem-loop real-time quantitative RT-PCR, and a possible miRNA-target regulatory network in litchi browning and senescence was proposed. Our study provides basic information for further understanding of the miRNA-mediated senescence in harvested litchi fruit and offers a new theoretical strategy to postpone the senescence of harvested horticultural products.

## Results

### Analysis of small RNA library datasets from litchi fruit

**Fig. 1:**
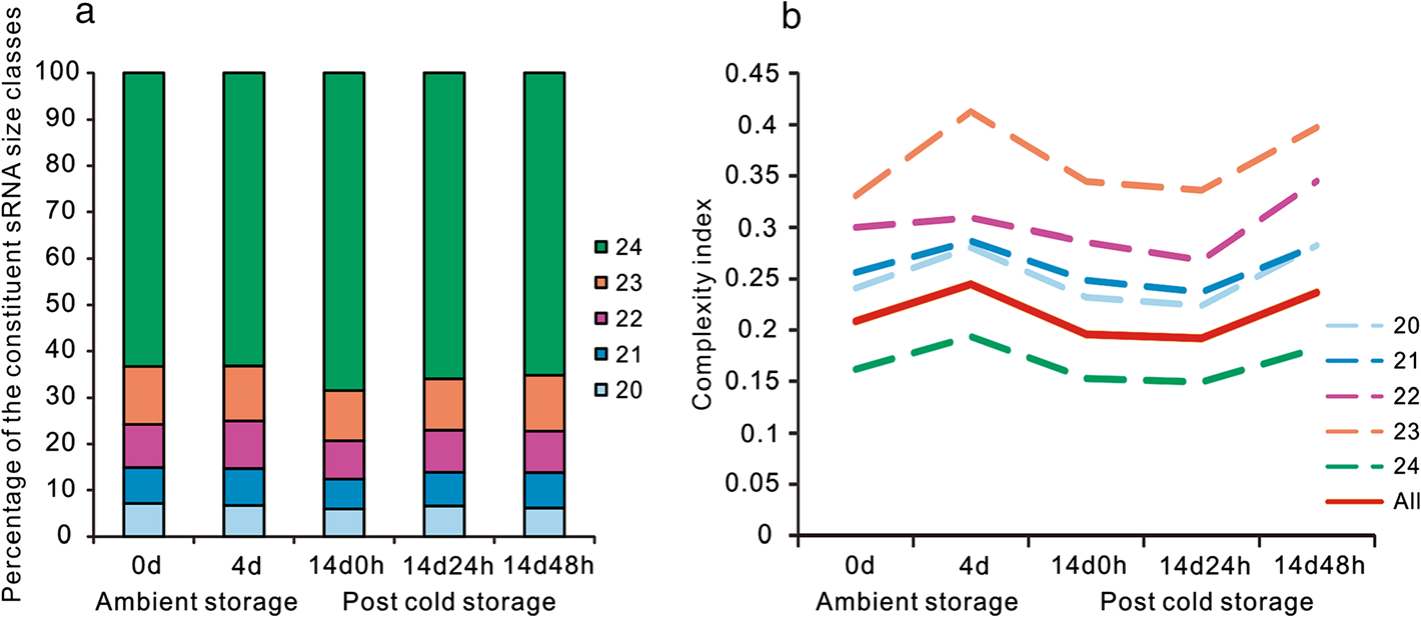
Distribution and complexity variation of sRNA in litchi pericarp. (**a**) Size class distribution of 20 ~ 24-nt sRNA reads during ambient and post-cold storage. The composition of sRNA classes is shown for each of the storage time points, defined as 0 d and 4 d at 25 °C, and 0 h, 24 h and 48 h at 25 °C after 2-week cold storage at 1 °C. rRNA and tRNA sequences are removed. (**b**) Change of the complexity for different size classes and all sRNA reads during ambient and post-cold storage. The complexity index is calculated as unique read count divided by total read count for all and each size class separately. Higher index represents more diverse sRNA sequence composition

A total of approximately 45 million reliable reads were obtained from high-throughput sequencing of 0 d-, 4 d-, 14 d 0 h-, 14 d 24 h-, and 14 d 48 h-derived sRNA libraries from litchi pericarp (Additional file 1). All of the reads were first processed by removing the 3′ adaptor sequence, and reads with low complexity and those homologous to t/rRNAs were excluded. Approximately 10 % of the reads aligned to the litchi unigene assembly with up to one mismatch (Additional file 1), and reads of 20 ~ 24 nucleotides were used for further analyses. The length distribution of the sRNA from five libraries showed an uneven pattern. In general, the majority of the small RNAs were distributed from 20 to 24-nt in size, and the most abundant was 24-nt, followed by 22-nt and 23-nt (Fig. 1a). One possible cause of the observed changes in the predominance of different groups of mature sRNAs could be the differential activity of the various sRNA biogenesis pathways. The size of mature sRNAs and the complexity of produced sRNA proportions are two key features that can distinguish various sRNA pathways. We observed in our data that 24-nt sRNAs were the most abundant class of sRNAs, which was consistent with previous reports [24]. This bias towards 24-nt sRNAs was particularly strong in litchi just after cold storage. Twenty-one-nucleotide sRNAs, on the contrary, showed a decreased abundance upon cold storage (Fig. 1a). This tradeoff suggested that different sRNA pathways are interlinked during the storage. Unexpectedly, 21-nt sRNAs in our study were less abundant than 22-nt and 23-nt sRNAs, suggesting that litchi may only harbor a limited number of miRNAs or that the length of miRNAs in litchi may vary greatly. As shown in Fig. 1b, we noticed an increase in the complexity indexes of all classes of sRNAs as the storage time progressed, regardless of whether the fruit was at ambient temperature or at post-cold storage shelf life, indicating a shift towards a more heterogenized population of sRNAs when the fruit senesced. Furthermore, it was strikingly strange that 24-nt sRNAs showed the lowest complexity index while other classes of sRNAs showed the longer size the more diverse sRNA sequence composition (Fig. 1b). This finding was different from those of most reports in other plants and reflected a unique feature of litchi sRNAs.

### Identification of known and novel miRNAs in litchi fruit

**Fig. 2:**
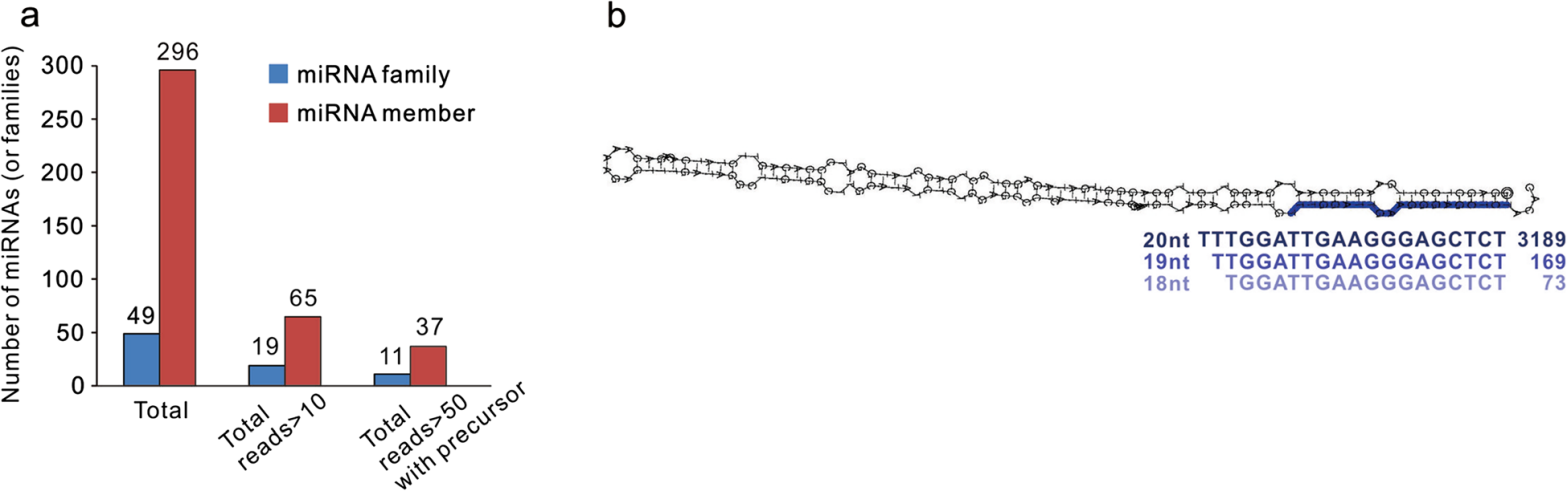
Conserved litchi miRNAs. (**a**) Bar plot showing the number of conserved miRNA families and members identified from litchi pericarp. (**b**) Stem-loop structure of lch-miR159 precursor, which comes from litchi unigene No. 45530. Isoforms of lch-miR159, showing a variable number of uridines at the 5′ end, are detected with highly variable abundances

Known miRNAs in litchi fruit were identified by matches to sequences from the miRNA repository (miRBase 20, [31]), allowing up to two mismatches. In total, 296 miRNAs were identified, among which 65 miRNAs from 19 families showed >50 total reads and 37 miRNAs from 11 families showed >10 total reads, and precursor sequences were also identified (Fig. 2a). Based on precursor sequences, these 296 miRNAs were classified into 49 miRNA families. Expression levels of the known miRNAs, as reflected by normalized reads, showed great variation among families in fruit samples at different time points. The highest read abundance was detected for miR319, miR396 and miR159, ranging from 16,155 to 5,466, which was many times more than other relatively abundant miRNA families, including miR393, miR166, miR162, and miR160, whose total abundance ranged from 159 to 534 (Additional file 2). These miRNAs were highly conserved in a variety of plant species. In addition, many less-conserved miRNAs found only in a few plant species were also identified in our dataset, such as miR403, miR858, miR477, miR530, and miR894, with a total abundance ranging from 37 to 217 (Additional file 2). Interestingly, we noticed a prevalent isoforming process for lch-miR159 precursors from litchi unigene no. 45530, which yielded precisely three miR159 isoforms with variable numbers of uridines at the 5′ end and highly variable abundances (Fig. 2b). There were more sRNA reads homologous to mature miR159 with one or two mismatches, which were also likely to be derived from this unigene (Additional file 2).

After excluding sRNA reads matched to known miRNAs, the remaining sRNAs (20 ~ 24-nt) were subjected to identifying novel miRNAs in litchi using MiRCat and RNAfold. The putative miRNAs were finally considered to be novel miRNA candidates when they met the criterion of plant miRNA identification [32]. This analysis resulted in 11 novel miRNA candidates in litchi from 10 unigenes (Table 1, Additional files 3 and 4), termed litchi-specific miRNAs. Only two of them belonged to the 21- or 22-nt classes of miRNAs, while all of the remaining ones belonged to the 23- or 24-nt class. Equal numbers of miRNAs were derived from the sense and antisense strands. Similar to lch-miR159, two litchi-specific miRNAs (lch-miRC5 and lch-miRC10), only differing in two nucleotides at the 5′ end, were identified to originate from the same unigene no. 41226 (Table 1). In general, the litchi-specific miRNAs were less abundant compared to most of the known miRNAs. For example, only lch-miRC1 had a total read abundance greater than 400, while eight of the 11 candidates yielded levels less than 120. Furthermore, most litchi-specific miRNAs showed a stage-specific expression pattern (Table 1).

**Table 1:** Predicted candidate litchi-specific miRNAs

Name	Sequence	Len	Unigene	Match site	Str	Normalized abundance					
						0d	4d	14d0h	14d24h	14d48h	Total
lch-miRC1	TGGGTGAAAGATGCAGCAAAATCT	24	lychee_29764	294	+	72.4	53.5	53.6	103.8	130.9	414.2
lch-miRC2	TTCGATTCGAACCCAGAGATGTCT	24	lychee_3356	8439	+	28.8	29.4	63.8	53.1	41.6	216.7
lch-miRC3	TTGTGGTGCTATTGTTTCTCCTCT	24	lychee_28612	3025	+	14.9	21.7	23.5	29.3	25.7	115.1
lch-miRC4	TTCAAGACAACAACTATTGGCTCT	24	lychee_43109	2259	+	47.1	26.4	79.6	82.7	34.8	270.7
lch-miRC5	CCTGTTGAGCTTGACTCTAGTCT	23	lychee_41226	539	−	21.5	6.5	12.9	20.4	5.3	66.6
lch-miRC6	CGAAAAGAACTCTGACTGGTCT	22	lychee_57528	1330	−	18.1	5.7	35.5	40.8	7.5	107.6
lch-miRC7	ATTTGGTAGTAGCTGAGATTCTCT	24	lychee_25799	986	+	13.1	3.4	6.8	19.9	3.3	46.6
lch-miRC8	CTATCAAACGATGATTGTTGGTCT	24	lychee_32974	83	−	2.0	3.1	9.1	8.1	1.3	23.6
lch-miRC9	ATTAAAGGAAGAAAAAGGACCTCT	24	lychee_2125	101	−	7.4	0.5	0.0	10.3	1.0	19.2
lch-miRC10	TGTTGAGCTTGACTCTAGTCT	21	lychee_41226	539	−	12.5	4.7	7.7	10.3	3.3	38.6
lch-miRC11	CGGAGAAGGGCAATTACTCATTCT	24	lychee_25026	5635	+	1.4	0.3	2.0	3.0	1.2	7.9

### Validation of identified litchi miRNAs by stem-loop qRT-PCR

**Fig. 3:**
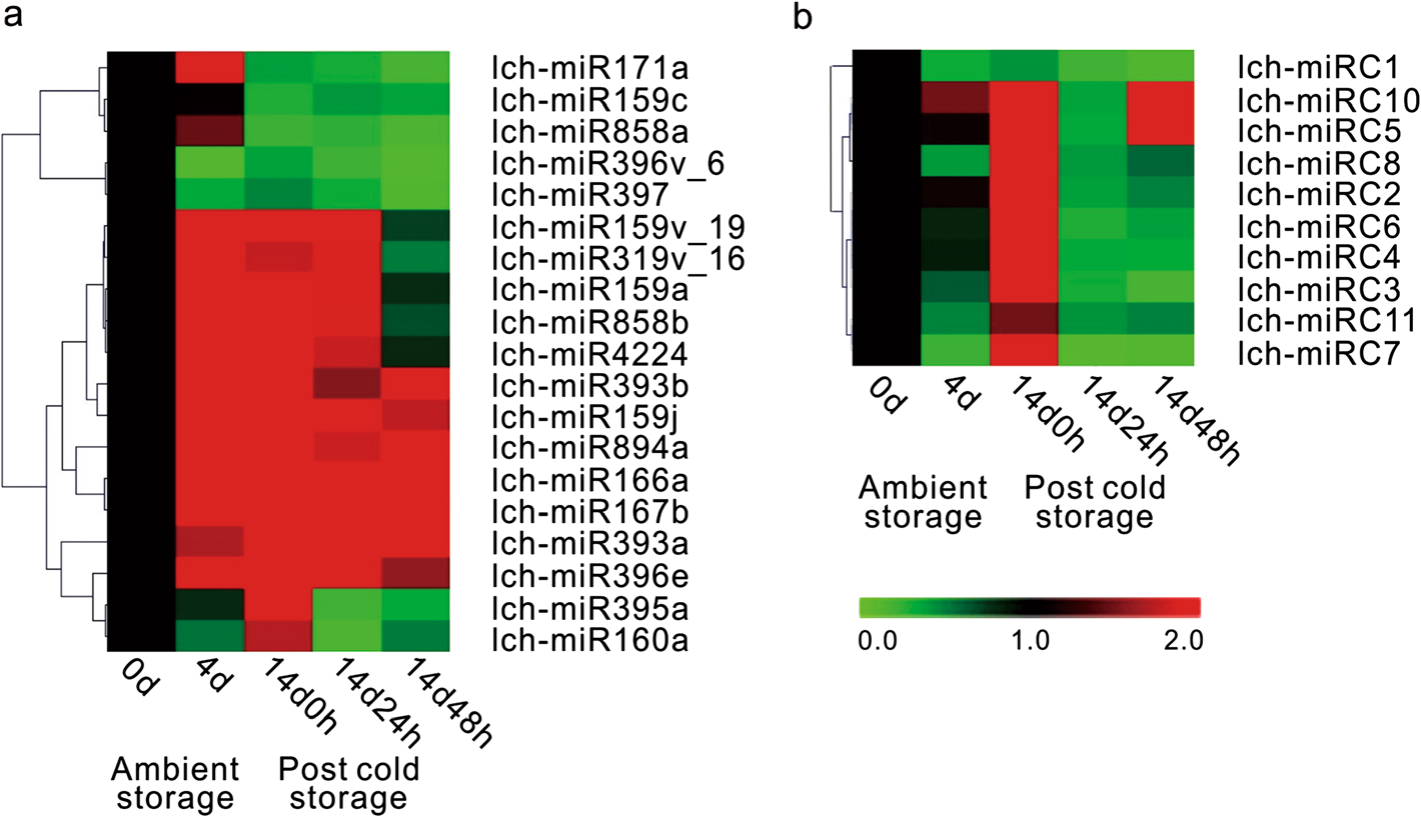
qPCR analysis of relative expression of (**a**) conserved and (**b**) novel miRNAs. The bar represents the scale of relative expression levels of miRNAs. Up (*red*) or down (*green*) regulation in expression are based on normalized data (color bar at the bottom) generated by MeV4.9. Each column represents a sampling point, and each row represents a single miRNA. The relative expression is normalized to actin and the normalized miRNA expression at day 0 is arbitrarily set to 1

To confirm the existence and expression of the above litchi miRNAs, 29 miRNAs (19 known miRNAs and 10 novel miRNAs) were selected for stem-loop qRT-PCR analysis (Fig. 3). In general, these miRNAs all showed significant expression changes during different storage periods compared with the levels at 0 d. Specifically, 23 miRNAs were up-regulated at 14 d 0 h post-cold storage, among which the relative expression levels of lch-miR396e, lch-miRC10 and lch-miRC5 were 11.0, 12.7 and 14.5, respectively. Notably, the expression of most miRNAs changed quickly when stored at 25 °C for 24 h after 14 d at 1 °C. The expression levels of lch-miR396e, lch-miRC10, and lch-miRC5, declined by 9.07-, 12.35- and 14.16-fold, respectively (Fig. 3). In addition, in contrast to other miRNAs, lch-miR396v_6, lch-miR397, lch-miRC1 were consistently down-regulated. Furthermore, some miRNAs were sequenced in low or even no reads, but they were detected by qRT-PCR, such as lch-miR159v_19, lch-miR319v_16 and lch-miRC11 (Fig. 3). Similarly, one miRNA (lch-miRC9) was not detected by qRT-PCR but was identified from sequencing data.

### Target genes of litchi miRNAs by degradome analysis

To identify targets of litchi miRNAs in our dataset, degradome sequencing was performed based on the information about miRNA and mRNA sequences obtained from previous transcriptome analysis. A total of 32,396,114 raw reads were generated with 7,677,220 (23.7 %) unique raw reads and 13,087,512 (40.4 %) cDNA mapped reads, of which 27,015 covered cDNAs were obtained. After processing and analysis by CleaveLand 3.0, 197 genes targeted by 167 known miRNAs were identified (Additional file 5). The identified targets were further classified into five categories (0–4) (Additional file 6). Among the targets for the known miRNA families, 40 belonged to category 0, representing the most abundant degradome tags corresponding to the cleavage site and matching cognate transcripts, and 198 belonged to category 2, whose cleavage abundance was higher than the median but below the maximum. The number of identified gene targets greatly varied for different miRNAs (Additional file 5). It was shown that most miRNAs could regulate more than one target gene, especially miRNAs in the miR396 family, such as lch-miR396v_1 (11 targets), lch-miR396v_7 (10 targets), lch-miR396v_10 (11 targets), lch-miR396v_12 (13 targets), and lch-miR396v_31 (15 targets). This corresponded to the high expression of miR396 family members throughout the storage of litchi fruit. Conversely, one target gene might be regulated by multiple miRNAs at either the same or different cleavage sites, particularly for lychee_43435 annotated as an MYB transcription factor, which was targeted by 31 miRNAs from the miR159 and miR319 families, suggesting that *MYB*s, as key transcription factors in various biological processes, are likely to be strictly controlled by miRNAs.

Gene targets were also identified for the 11 litchi-specific miRNAs. Of the five gene targets identified, one belonged to category 0 and two to category 2, while the remaining two fell into category 4 (Table 2). While most targets of the litchi-specific miRNAs had justifiable alignment scores, they were relatively poorly annotated; only two genes were annotated as a NAC transcription factor and a caleosin-related protein, respectively (Table 2).

**Table 2:** Targets of litchi miRNAs (or families)

miRNA	Target	Align score	Category	Cleavage abundance (tpb)	Target annotation
Conserved targets for conserved miRNAs					
lch-miR319	lychee_2636	3.5	2	229.2261509	transcription factor TCP4
lch-miR393	lychee_55456	1	0	6189.106073	auxin signaling F-box 2
lch-miR397	lychee_9267	4	2	1910.217924	laccase
Non-conserved targets for conserved miRNAs					
lch-miR159	lychee_2636	3.5	2	229.2261509	transcription factor TCP4
	lychee_39039	4	2	382.0435848	transcription factor WRKY28
lch-miR396	lychee_55389	1	3	229.2261509	cysteine protease CP1
	lychee_53269	3	2	152.8174339	chalcone isomerase
	lychee_9553	3.5	2	21012.39716	serine/threonine-protein kinase SRK2A
	lychee_15210	4	2	840.4958865	transcription factor AP2/ERF
	lychee_31188	4	2	764.0871695	transcription factor WRKY7
	lychee_16455	3.5	2	229.2261509	callose synthase 1
Targets for other known miRNAs					
lch-miR5252	lychee_39716	3.5	2	458.4523017	glucan endo-1,3-beta-D-glucosidase
lch-miR858	lychee_18326	2.5	0	7258.82811	MYB29 family protein
	lychee_30086	4	0	3514.80098	transcription repressor MYB1
Targets for litchi-specific miRNAs					
lch-miRC6	lychee_57528	0	0	1203.848091	hypothetical protein
lch-miRC7	lychee_30135	2.5	2	586.4900957	NAC domain-containing protein
	lychee_37170	3.5	4	30.86789977	unknown
	lychee_7801	2.5	4	30.86789977	Caleosin related protein
lch-miRC10	lychee_4839	4	2	1142.112292	predicted protein

To better understand the potential biological functions of litchi miRNAs, the identified target genes were subjected to Gene Ontology (GO) and Kyoto Encyclopedia of Genes and Genomes (KEGG) analyses (Additional file 7). Among all functional categories, targets involved in nuclear components occupied the highest frequency, followed by targets participating in DNA-dependent transcription, DNA-dependent regulation of transcription, and DNA binding, respectively, indicating that most targets were transcription factors. In addition, there were some biological processes of low frequency but closely related to litchi fruit browning and senescence. These included a defense response to bacteria/fungi, the auxin/ethylene signaling pathway, cell wall organization, and anthocyanin metabolism. KEGG analysis demonstrated that these targets played roles in five aspects, including metabolism, genetic information processing, environmental information processing, cellular processes, and so on. In the KEGG functional classification, some classes of target genes related to senescence were also found, such as bacterial invasion of epithelial cells, flavonoid biosynthesis, and plant-pathogen interaction (Additional file 7).

### Screening of miRNAs and their targets involved in the post-harvest litchi fruit senescence and RLM-5′-RACE validation

**Fig. 4:**
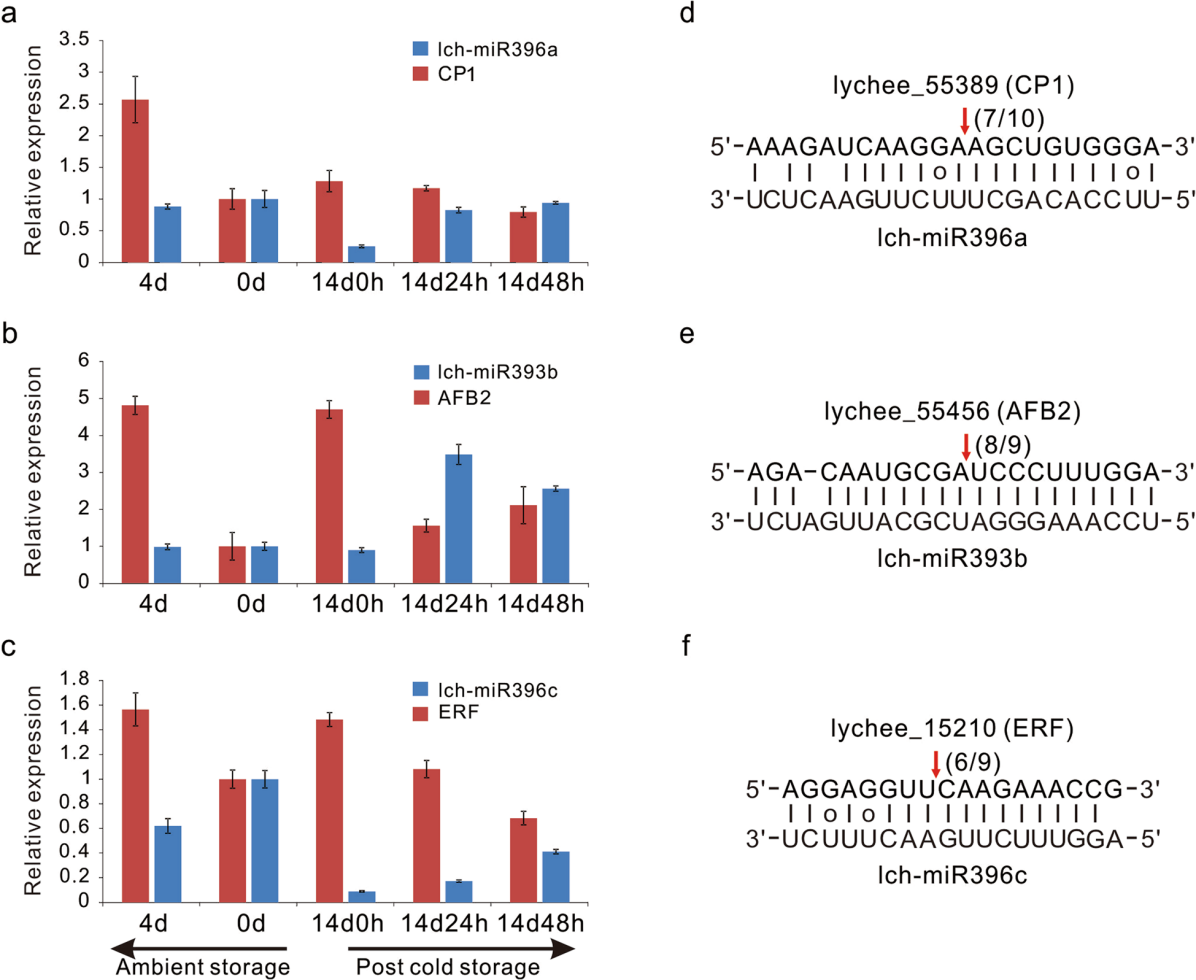
Differential expression of miRNA-targets in senescent litchi during ambient and post-cold storage and RLM-5′-RACE validation. Relative expression is normalized to actin, and the normalized miRNA expression at day 0 is arbitrarily set to 1. The data are the mean ± standard deviation (*n* = 3). (**a**) lch-miR396a targeting cysteine protease gene, which mediates proteolysis. (**b**) lch-miR393b targeting auxin signaling F-box2, which mediates auxin signal transduction. (**c**) lch-miR396c targeting transcription factor ERF, which mediates the process of senescence. (**d-f**) RLM-5′-RACE validation of the CP1, AFB2 and ERF cleavage sites by lch-miR396a, lch-miR393b and lch-miR396c, respectively. The number next to the arrow indicates the number of sequences found at the exact cleavage site

To screen miRNAs and their target genes that were most likely responsive to post-harvest litchi fruit senescence, several steps were carried out. First, based on GO/KEGG functional classifications and non-redundant (Nr) annotation, 62 target genes related to fruit browning and/or senescence were selected (Additional file 8). These targets were mainly associated with cell wall organization, pathogen defense, hormone signaling, anthocyanin metabolism pathway, and energy regulation. Here, it was necessary to note that some of these 62 targets were cleaved by multiple miRNAs, such as lychee_55456 targeted by 10 miRNAs, and some targets were predicted to be involved in more than one process, such as lychee_9267 participating in the organization of both the cell wall and anthocyanin. Second, the above targets were further filtered according to their expression obtained by transcriptome sequencing. For the five sampling time points, genes with the highest expression level of no more than 30 rpkm were excluded. Thus, 40 target genes remained after this step (Additional file 9). Third, the number of targets was further decreased, combining the relative expression of their corresponding miRNAs via stem-loop qPCR (Fig. 3, Additional file 10). The miRNAs that showed significant expression changes during storage were selected along with their targets. Finally, 10 miRNAs and 18 targets likely related to litchi fruit senescence were listed in Table 2. Afterwards, nine senescence-related target genes were selected for RLM-5′-RACE validation, and the cleavage sites of six targets were independently confirmed (Fig. 4d-f, Additional file 10).

## Discussion

### Litchi miRNAs with conserved and new gene targets

In this study, we used deep sequencing and computational analyses to identify 49 known miRNA families and 11 litchi-specific miRNAs (Fig. 2, Table 1, Additional file 2) in litchi. While the number of litchi miRNAs was limited, compared to those identified in other plant species [24], our study provides the most reliable and comprehensive list of identified miRNAs in litchi to date. The majority of these miRNAs showed stage-specific expression (Fig. 3). In litchi, we identified a total of 197 gene targets for 167 known miRNAs using degradome analysis (Table 2, Additional file 5), and the majority of these targets are conserved in plant species, indicating broad conservation of the known miRNA regulatory roles in plants. However, a few of the known miRNAs, including miR159 and miR396, were found to target additional genes in litchi, which have not been previously reported. Thus, while these known miRNAs conserve their gene targets, they also appear to have an expanded target population in litchi.

Although many newly evolved miRNAs are believed to have no biological function due to low expression or no identified target, some of these miRNAs have been shown to regulate specific genes or gene families in various species [33–35]. In our degradome data, three of the 11 litchi-specific miRNAs were found to target specific genes, suggesting a role for these litchi-specific miRNAs in the control of transcription and stress responses, which may be unique to litchi fruit senescence (Table 2). The inability to detect targets for the remaining litchi-specific miRNAs may be due to a low level of expression or the stage-specific nature of their target genes.

### MiRNAs participate in proteolysis during litchi fruit senescence

Senescence is a physiological process that has a close relationship with proteolysis in plants. Senescence-associated proteolysis could trigger a massive degradation network of relative proteins and eventually lead to cell death [36]. In this study, the *CP1* gene was down-regulated after cold storage compared with that at 0 d. However, when the fruit was transferred from cold to room temperature, *CP1* showed an obvious increase in expression during shelf life (Fig. 4a). Such a fluctuation in *CP1* expression was partly supported by previous results, which confirmed that low temperature can inhibit fruit senescence while accelerated fruit senescence is associated with post-cold storage [37]. In addition, lch-miR396a identified to target *CP1* showed an opposite trend of expression by qPCR, especially during cold and post-cold storage (Fig. 4a), which exactly fit the mechanism of miRNA action [18, 21]. However, the miRNA was up-regulated 2.5-fold at 4 d during room temperature storage, while its target *CP1* showed no change in expression, implicating that *CP1* activation or suppression may also depend on pH, the action of other proteases and the cellular or extracellular environment.

### MiRNAs participate in hormone pathways during litchi fruit senescence

Among plant hormone pathways, the relationship between auxin and miRNAs has been well studied [38]. In our study, lch-miR393b was found to target lychee_55456, which is annotated as AFB2 (Table 2). At the five sampling time points, the expression of miR393 was essentially complementary to that of the target *AFB2* (Fig. 4b). This trend of expression was similar to previous results [39]. It is also worth mentioning that the expression level of miR393 changed sharply during both ambient storage and post-cold storage, with the relative expression increasing fivefold compared with that at 0 d (Fig. 4b). Later, this miRNA expression declined significantly, nearly fourfold after post-cold storage at 24 h. Conversely, the expression of its target gene *AFB2* increased almost fourfold during the same period. The significant variation in expression suggested that miR393 played a key role in the post-harvest senescence of litchi fruit by mediating auxin signaling. The auxin response has been suggested to be common between *Arabidopsis* silique senescence and the over-ripening process in tomato [40]. However, the effect of auxin on the delay or acceleration of fruit senescence may depend on various factors. It has been reported that auxin can inhibit the ripening process in tomato [41], while low levels of auxin are required for seed dehiscence in *Arabidopsis* [42].

### A miRNA-TF network may contribute to the regulation of litchi fruit senescence

As main target genes of most miRNAs, transcription factors (TFs) have a crucial function in a wide range of biological processes. In this study, we also identified several miRNAs that regulate downstream TFs that are associated with litchi fruit senescence (Table 2, Fig. 4c).

Previous studies have shown that ERFs are key elements in integrating ethylene and jasmonic acid pathways for fruit ripening and senescence [43, 44]. In addition to miR172, other miRNAs, such as miR156, miR159, miR393, miR396, have recently been predicted to inhibit *Hevea* transcripts of 29 *HbAP2/ERF* genes [45]. In our study, lch-miR396c was identified to slice lychee_15210, which was annotated as a transcription factor containing an AP2/ERF domain. The miRNA was up-regulated regardless of whether the fruit was stored at 1 °C or 25 °C, while the expression continued to decline when fruit was kept for 24 h and 48 h at 25 °C after cold storage (Fig. 4c). At the same time, the target gene *ERF* showed an opposite trend in expression, which demonstrated the regulation by lch-miR396c and their joint participation in litchi fruit senescence. However, because different ERF gene family members can have either positive or negative effects on the downstream gene expression [46], the exact role of miR396 in litchi fruit senescence remains to be further determined.

**Fig. 5:**
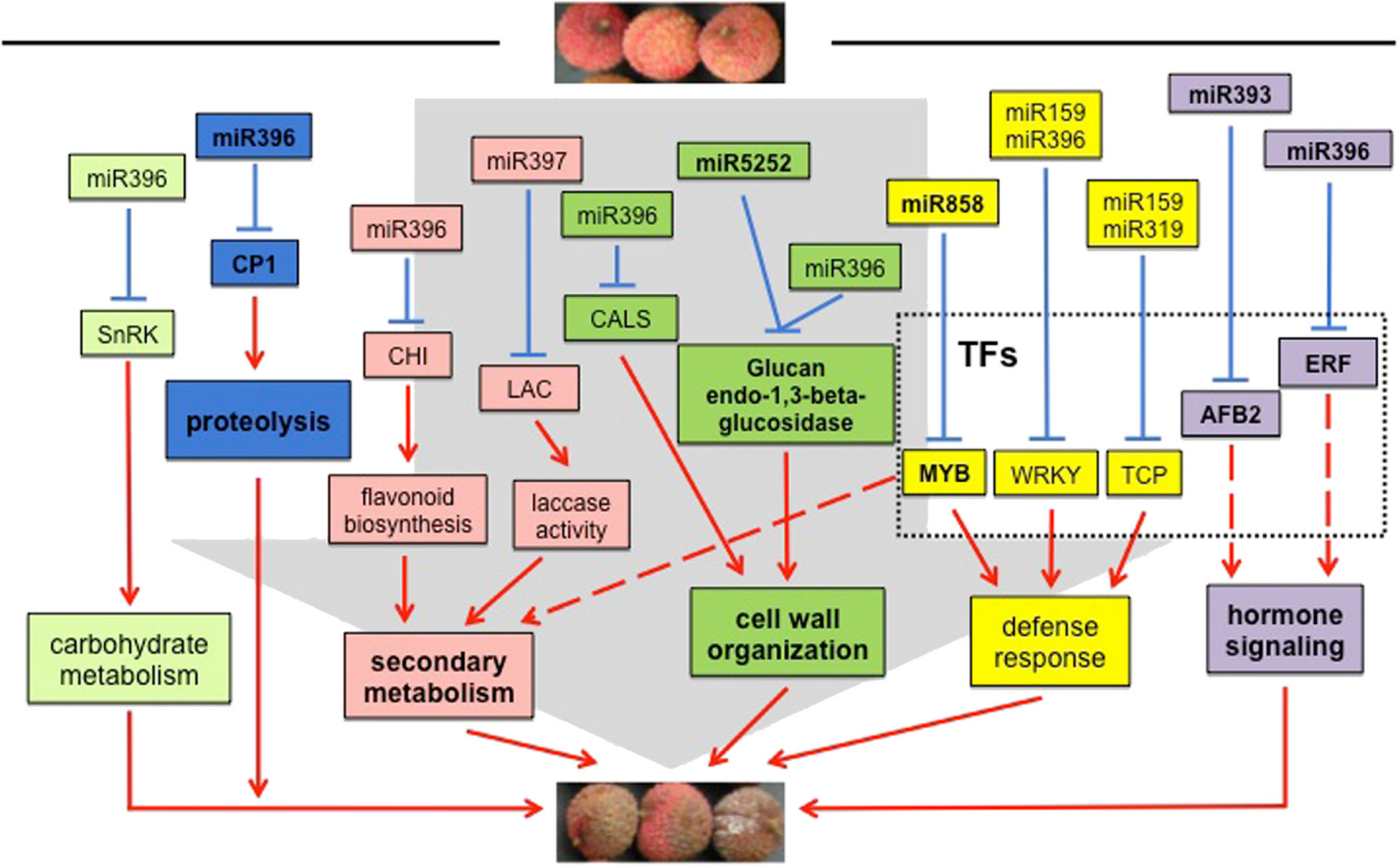
A proposed miRNA-regulatory network during litchi fruit senescence. Those miRNAs and their target genes validated by 5′-RACE are highlighted in bold in the figure. It is proposed that these miRNAs and their targets may interplay in proteolysis, hormone signaling, cell wall organization and secondary metabolism, thus collectively regulating the process of post-harvest litchi fruit senescence

Taken together, we proposed that these miRNAs and their target genes may interplay in proteolysis, hormone signaling, cell wall organization and secondary metabolism, thus collectively regulating the process of post-harvest litchi fruit senescence (Fig. 5).

## Conclusions

In this study, we constructed five small RNA libraries from litchi pericarp at different storage periods and a mixed degradome library. Through sequencing and analysis, 296 miRNAs belonging to 49 conserved miRNA families and 11 novel miRNAs were first obtained in litchi. Among these, 170 miRNAs were identified to cleave 202 targets. After a series of analyses, 14 miRNA-target pairs were found to be involved in fruit senescence. We proposed the possible regulatory network that these miRNAs and their targets collectively mediated the post-harvest litchi fruit senescence (Fig. 5). These findings offered new ideas that miRNAs are actively involved in litchi fruit senescence by regulating the upstream transcription factor genes. We may take advantage of specific miRNAs as a marker for early senescence prediction and further as the candidate for miRNA-based posttranscriptional gene silencing to delay fruit senescence.

## Methods

### Plant material, sample treatment and RNA isolation

Litchi (*Litchi chinensis* Sonn. cv. Huaizhi) trees were grown in a commercial orchard in Guangzhou, China. After harvesting at the same stage of maturity, unblemished and disease-free fruit with uniform color were selected. To minimize the microbe effect, all fruit were dipped in 500 mg/L thiabendazole (Syngenta Crop Protection, Shanghai, China) for 3 min and air dried for 1 h at 25 °C. Each 30 fruit was packed in 0.015-mm-thick polyethylene bags, and three bags were used as biological replicates for each treatment. In this study, ambient temperature (25 °C) was selected just to simulate the shelf life temperature in practical application, and cold storage at 1 °C can most effectively control fruit senescence and maintain fruit quality. The bagged fruit was then divided into two groups. One group was stored at 25 °C for 0 and 4 days. The other group was stored at 1 °C for 2 weeks and then transferred to room temperature (25 °C) for a shelf time of 0, 24 h and 48 h. Pericarp tissues from the five sampling points were immediately frozen, crushed in liquid nitrogen, and stored at −80 °C for RNA extraction, small RNA and degradome sequencing. Total RNA was isolated from litchi pericarp using a previously described method [47]. The integrity and quality of the RNA were checked using a NanoDrop spectrophotometer (Thermo Fischer Scientific, Wilmington, DE, USA) and agarose gel electrophoresis. Only RNA samples with A260/A280 ratios between 1.9 and 2.1 were used.

### Small RNA library construction, sequencing and analysis

Small RNA libraries were constructed using Illumina Small RNA Sample Prep Kit, following the manufacturer’s instructions. In brief, 16 ~ 30-nt small RNAs were isolated by 15 % denaturing polyacrylamide gel electrophoresis from the total RNA. Subsequently, the small RNAs were ligated with 5′ and 3′ RNA adapter, reverse transcribed into cDNA, PCR amplified and sequenced using Illumina Genome Analyzer II platform.

To identify known miRNAs in the litchi pericarp, sRNA raw reads were preprocessed using the Illumina Genome Analyzer Pipeline. Then, all of the clean reads were mapped to miRBase 20 (http://www.mirbase.org/), allowing up to two mismatches. For novel miRNA identification, MiRCat (http://seqanswers.com/wiki/MiRCat/URL_0) was applied to fold flanking genome sequences of the unique small RNAs, followed by secondary structure prediction using RNAfold (http://rna.tbi.univie.ac.at/cgi-bin/RNAfold.cgi). Further criteria for annotation of plant miRNAs according to Meyers et al. (2008) were used to screen for candidate miRNAs [48].

### Transcriptome library construction, sequencing and analysis

Five cDNA libraries were created as described by Li et al. [49], with fruit samples from the same time points as the sRNA libraries, and the samples were sequenced using Solexa HiSeq™ 2000 with a paired-end strategy. After removing adaptors and low-quality reads, all of the remaining reads were processed with de novo assembly (BGI, Shenzhen, China). The assembled unigenes were then annotated via BlastX according to National Center for Biotechnology Information (NCBI) NR protein database (http://www.ncbi.nlm.nih.gov), KEGG pathway (http://www.genome.jp/kegg) and COG database (http://www.ncbi.nlm.nih.gov/COG). Additionally, GO functional classification was performed using Blast2GO and WEGO [50, 51]. Finally, gene expression at five time points was profiled through normalized sequencing reads matching the unigene assembly.

### Degradome library construction, sequencing and analysis

A degradome library from litchi pericarp was constructed as described recently [52] with minor modifications. Briefly, poly (A) mRNA was extracted after biotinylated random primers were mixed with total RNA and combined with dynabeads. The obtained mRNA fragment was ligated to a custom 5′ RNA oligonucleotide adaptor containing an MmeI site and reverse transcribed, followed by PCR amplification. The amplified library was then gel purified for sequencing on Illumina GAIIx (LC Sciences, Houston, TX, USA).

After adaptor sequences and low-quality reads were removed, all of the reads were analyzed to detect potentially cleaved miRNA targets via the CleaveLand 3.0 pipeline [53], with litchi miRNA and mRNA sequences as references. The miRNA to mRNA alignments were scored as follows: G:U pairs were scored as 0.5, while mismatched pairs or single nucleotide bulges were scored twice, respectively. The mismatched and G:U pairs within the core segment (at positions 2–13 nt) were scored twice. At this stage, to further validate miRNA targets and distinguish the cleavage site, t-plots were generated according to the abundance of the resulting mRNA tags relative to the overall degradome reads that aligned to the target transcript [54, 55]. All of the identified targets were then classified into five categories. In category 0, the most abundant tag was the only maximum on the transcript, which was located at the predicted miRNA guided cleavage site; compared with category 0, there was more than one abundant maximum in category 1; and in category 2, the abundance of cleavage tags was less than the maximum but higher than the median. If the abundance of cleavage tags was equal to or less than the median, it was indicated as category 3; when only one raw read was matched at the cleavage position of the transcript, it was grouped as category 4.

Similar to the annotation of the transcriptome, all of the identified targets were annotated by BlastX (E-value < 0.00001) to NR and KEGG, followed by GO term analysis. These results were helpful for uncovering the miRNA-target regulatory network based on biological processes, cellular components, molecular function, and so on.

### Validation of litchi miRNAs and their target mRNAs by qRT-PCR

To validate the existence and expression of the identified miRNAs and their target mRNAs, 30 known miRNAs, 10 litchi-specific miRNAs and 16 targets were selected for real-time quantitative PCR (qRT-PCR). Total RNA was extracted, and reverse transcription was performed with the PrimeScript™ RT reagent Kit (Takara, Dalian, China) according to the manufacturer’s instructions but using specific stem-loop RT primers for microRNAs (Additional file 11) and the oligo dT primer for target mRNAs. qPCR was performed on an ABI 7500 systems (Applied Biosystems, Carlsbad, CA, USA) with SYBR Premix Ex Taq™ II (Takara, Dalian, China), according to the standard protocol. The reverse and forward primers for all selected miRNAs and targets are available in Additional file 11. The litchi *Actin* gene was used as an internal reference to calculate the relative expression level of miRNAs and targets.

### Confirmation of miRNA-guided cleavage site by RLM-5′-RACE

Following the manufacturer’s instructions for the FirstChoice RLM-RACE Kit (Ambion, Austin, TX), 1 μg of mixed RNA isolated from litchi pericarp at the five sampling points was used for ligating 5′ RNA adaptors at 15 °C overnight. Then the oligo dT primer was used to synthesize the first cDNA with M-MLV Reverse Transcriptase. Gene-specific primers (Additional file 11) were designed to conduct nested PCRs, and PCR products were gel purified, cloned into the pGM-18 T vector (TaKaRa) and sequenced.

### Availability of supporting data

The small RNA and degradome sequencing data are available under NCBI-GEO accession no: GSE63658. (http://www.ncbi.nlm.nih.gov/geo/query/acc.cgi?acc=GSE63658).

## Additional files

Additional file 1:
**Statistics of sRNA sequences from litchi pericarp.**
Additional file 2:
**Conserved miRNAs identified from litchi pericarp.**
Additional file 3:
**Detailed table of predicted candidate litchi-specific miRNAs.**
Additional file 4:
**Predicted secondary stem-loop structures of the litchi-specific miRNAs.** This file contains all the stem-loop structures for the litchi-specific miRNAs, with the miRNA sequence denoted in blue. Additional file 5:
**Detailed table of degradome sequencing data.**
Additional file 6:
**T-plots for all targets of litchi miRNAs.** This file contains t-plots for all targets of litchi miRNAs. Signature abundance throughout the length of the indicated lychee unigene transcripts is plotted. Red line indicates signatures consistent with miRNA-directed cleavage. miRNA:mRNA alignment is shown on the top. Additional file 7:
**GO and KEGG functional classification for litchi miRNA targets.** This file contains gene functional enrichment analysis for both GO and KEGG pathways. The numbers of genes in the enriched functional classes are presented in the differently colored bars. Additional file 8:
**Targets related to litchi fruit senescence via gene functional analyses.**
Additional file 9:
**Targets in Additional file 8 with high expression abundance according to transcriptome sequencing.**
Additional file 10:
**Real-time quantitative PCR and RLM-5′-RACE confirming the expression of other miRNA-targets potentially involved in litchi fruit senescence.** This file contains both qPCR and 5′-RACE validations of selected litchi miRNA-target pairs. Relative expression is normalized to actin, and the normalized miRNA expression at day 0 is arbitrarily set to 1. The data are the mean ± standard deviation (*n* = 3). The number next to the red arrow above the miRNA:mRNA alignment indicates the number of sequences found at the exact cleavage site. Additional file 11:
**Primers for stem-loop qRT-PCR and RLM-5′-RACE**

## Abbreviations

GO: Gene Ontology
KEGG: Kyoto encyclopedia of genes and genomes
miRNA: microRNA
NCBI: National Center for Biotechnology Information
qRT-PCR: Quantitative real-time reverse transcription polymerase chain reaction
