# Supplementary material for: MicroRNAs and targets in senescent litchi fruit during ambient storage and post-cold storage shelf life
Source: BMC Plant Biol. 2015 Jul 16;15:181. doi: 10.1186/s12870-015-0509-2 (PMC4504174; doi:10.1186/s12870-015-0509-2)

Figure S1. Predicted secondary stem-loop structures of the litchi-specific miRNAs.

Name: lch-miRC1
Sequence: TGGGTGAAAGATGCAGCAAAATCT
Normalized abundance: 414.24

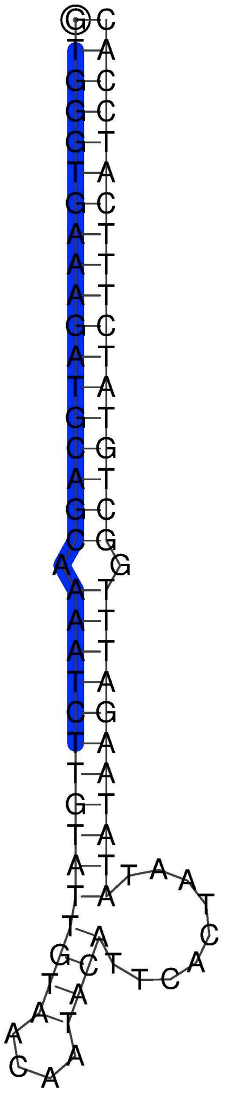

Name: lch-miRC2
Sequence: TTCGATTCGAACCCAGAGATGTCT
Normalized abundance: 216.73

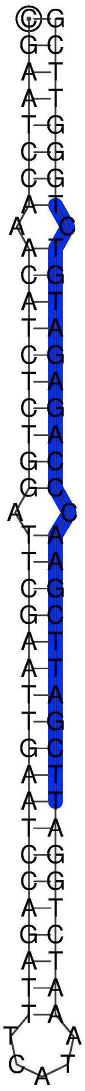

Name: lch-miRC3

Sequence: TTGTGGTGCTATTGTTTCTCCTCT

Normalized abundance: 115.11

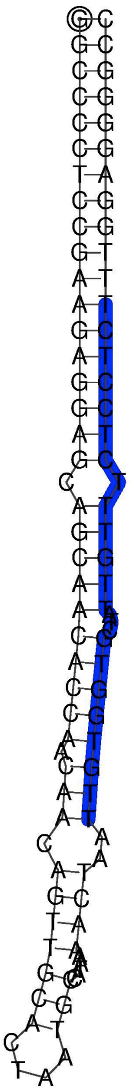

Name: lch-miRC4
Sequence: TTCAAGACAACAACTATTGGCTCT
Normalized abundance: 270.65

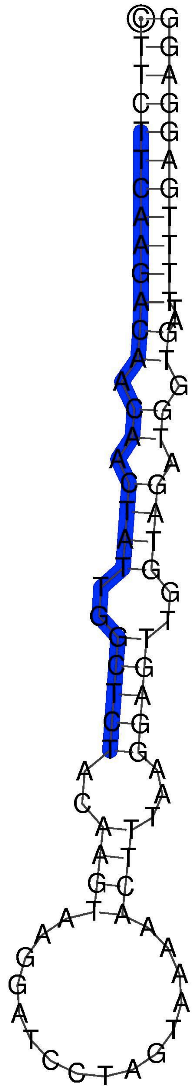

Name: lch-miRC5
Sequence: CCTGTTGAGCTTGACTCTAGTCT
Normalized abundance: 66.62

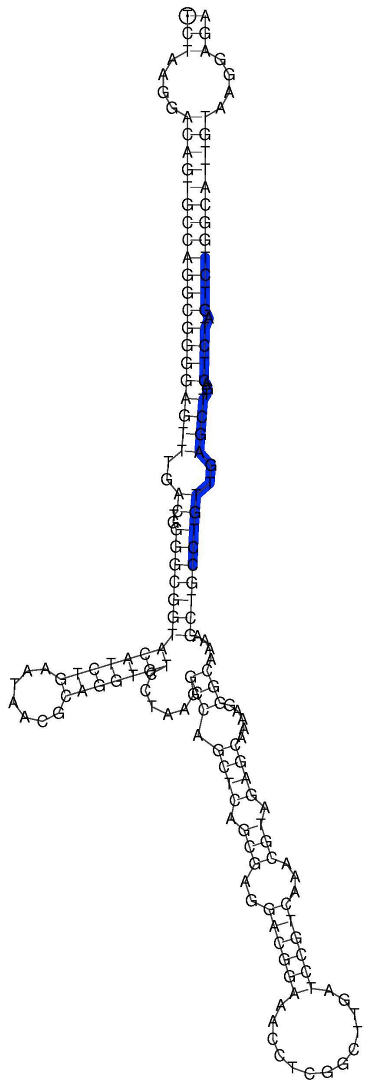

Name: lch-miRC6

Sequence: CGAAAAGAACTCTGACTGGTCT

Normalized abundance: 107.62

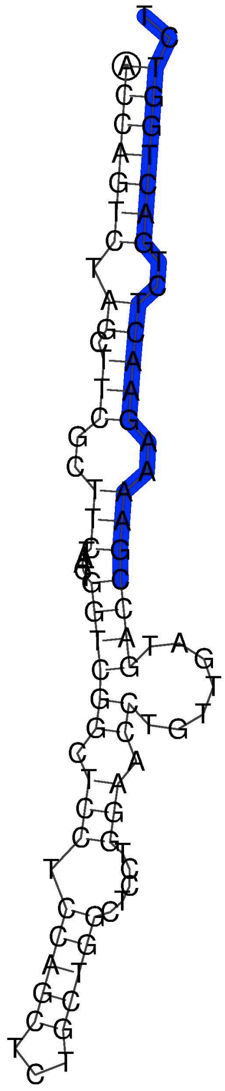

Name: lch-miRC7
Sequence: ATTTGGTAGTAGCTGAGATTCTCT
Normalized abundance: 46.58

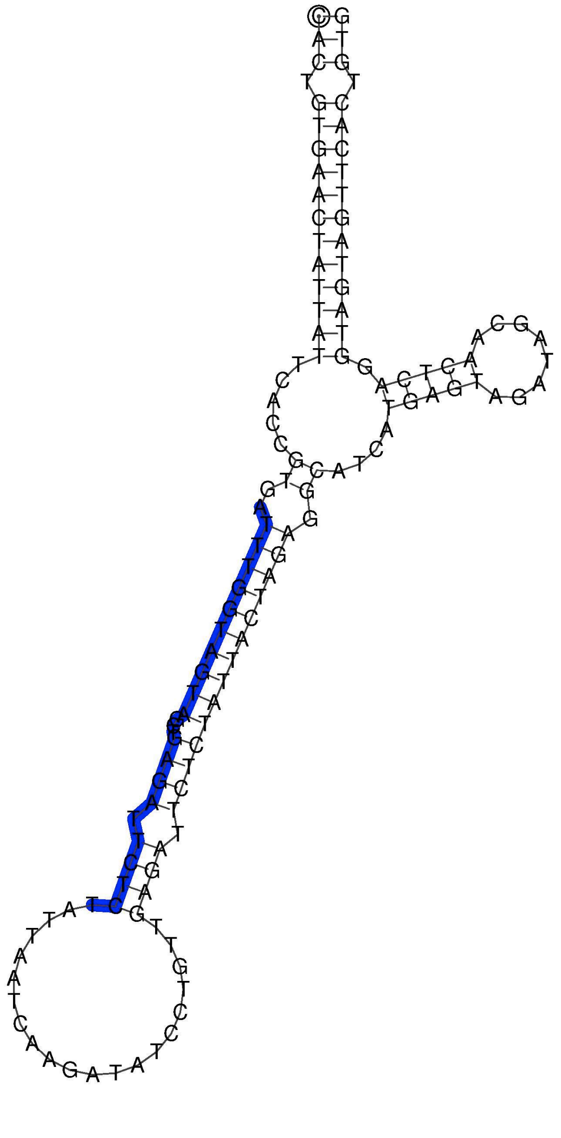

Name: lch-miRC8
Sequence: CTATCAAACGATGATTGTTGGTCT
Normalized abundance: 23.58

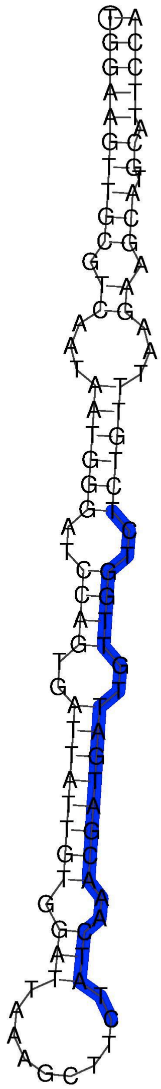

Name: lch-miRC9
Sequence: ATTAAAGGAAGAAAAAGGACCTCT
Normalized abundance: 19.18

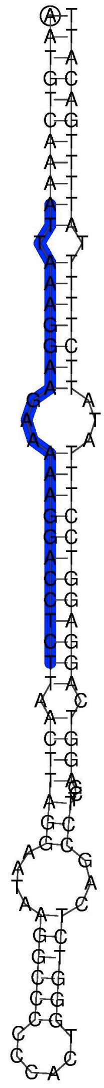

Name: lch-miRC10

Sequence: TGTTGAGCTTGACTCTAGTCT

Normalized abundance: 38.60

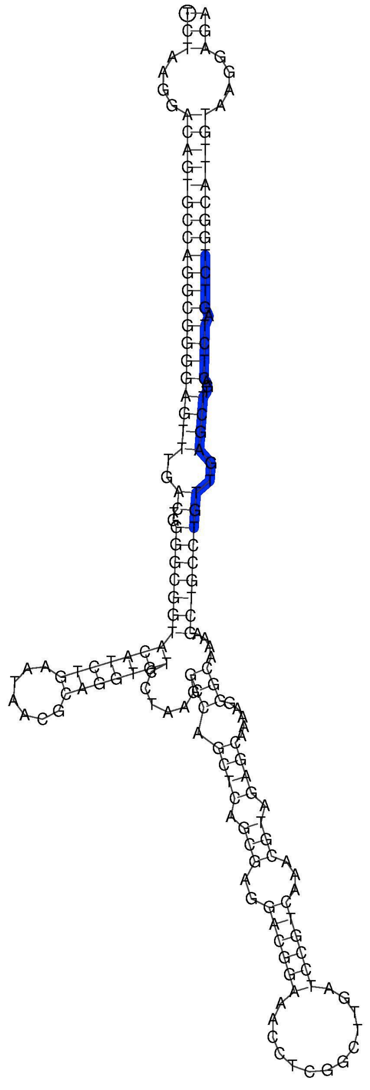

Name: lch-miRC11

Sequence: CGGAGAAGGGCAATTACTCATTCT

Normalized abundance: 7.87

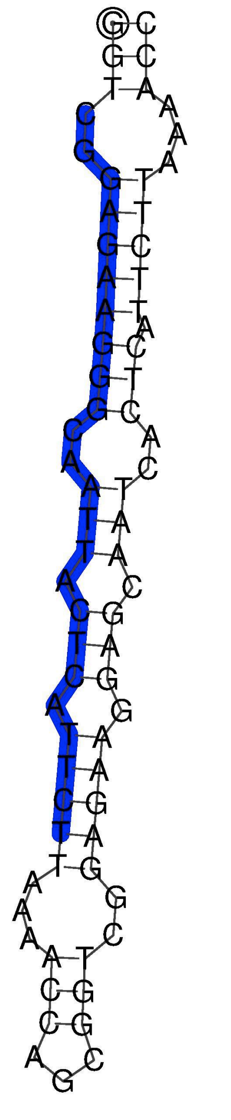

Supplement: Additional file 4: — Predicted secondary stem-loop structures of the litchi-specific miRNAs. This file contains all the stem-loop structures for the litchi-specific miRNAs, with the miRNA sequence denoted in blue. [file 12870_2015_509_MOESM4_ESM.pdf]
