# Supplementary material for: MicroRNAs and targets in senescent litchi fruit during ambient storage and post-cold storage shelf life
Source: BMC Plant Biol. 2015 Jul 16;15:181. doi: 10.1186/s12870-015-0509-2 (PMC4504174; doi:10.1186/s12870-015-0509-2)

Figure S2. T-plots for all targets of litchi miRNAs.

6_miR156_169_lychee_15999

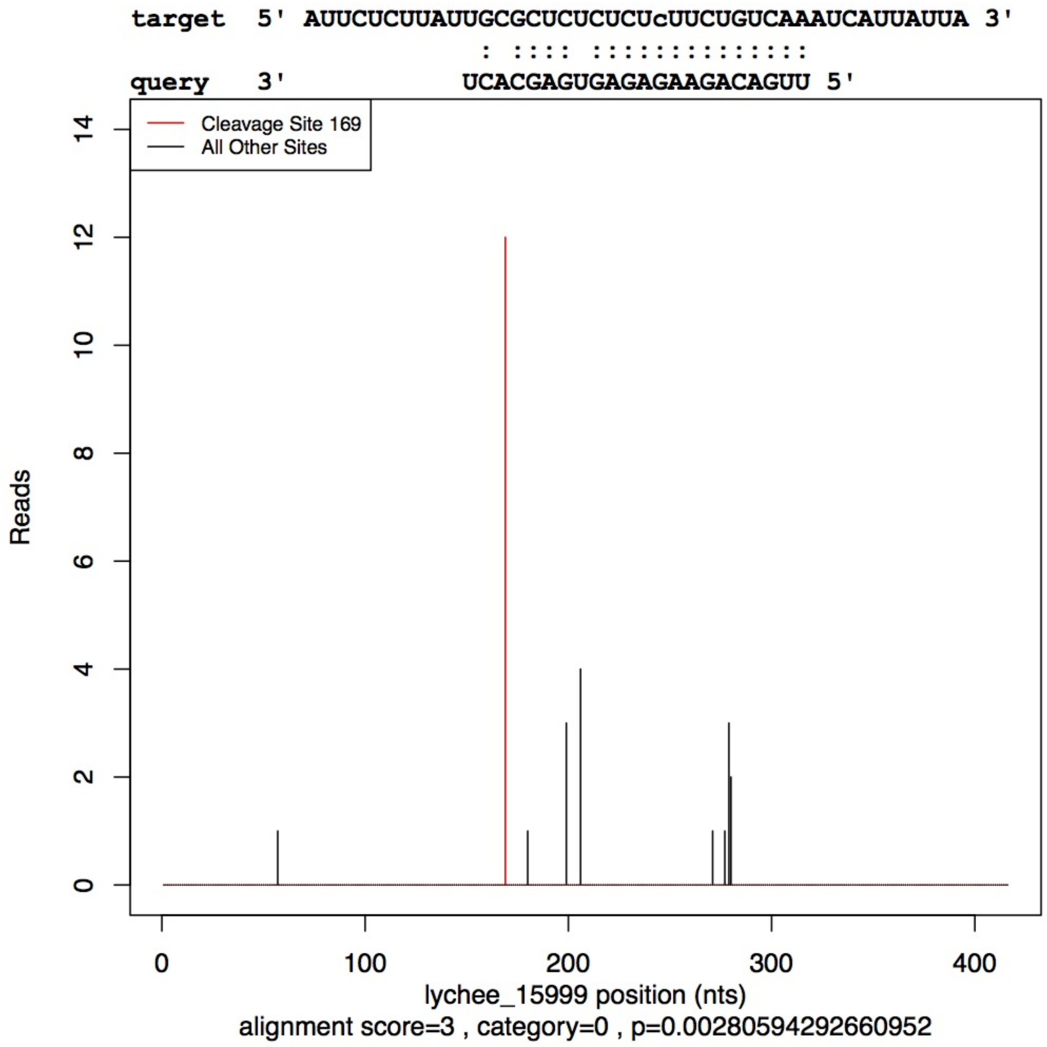

9_miR159_1692_lychee_43435

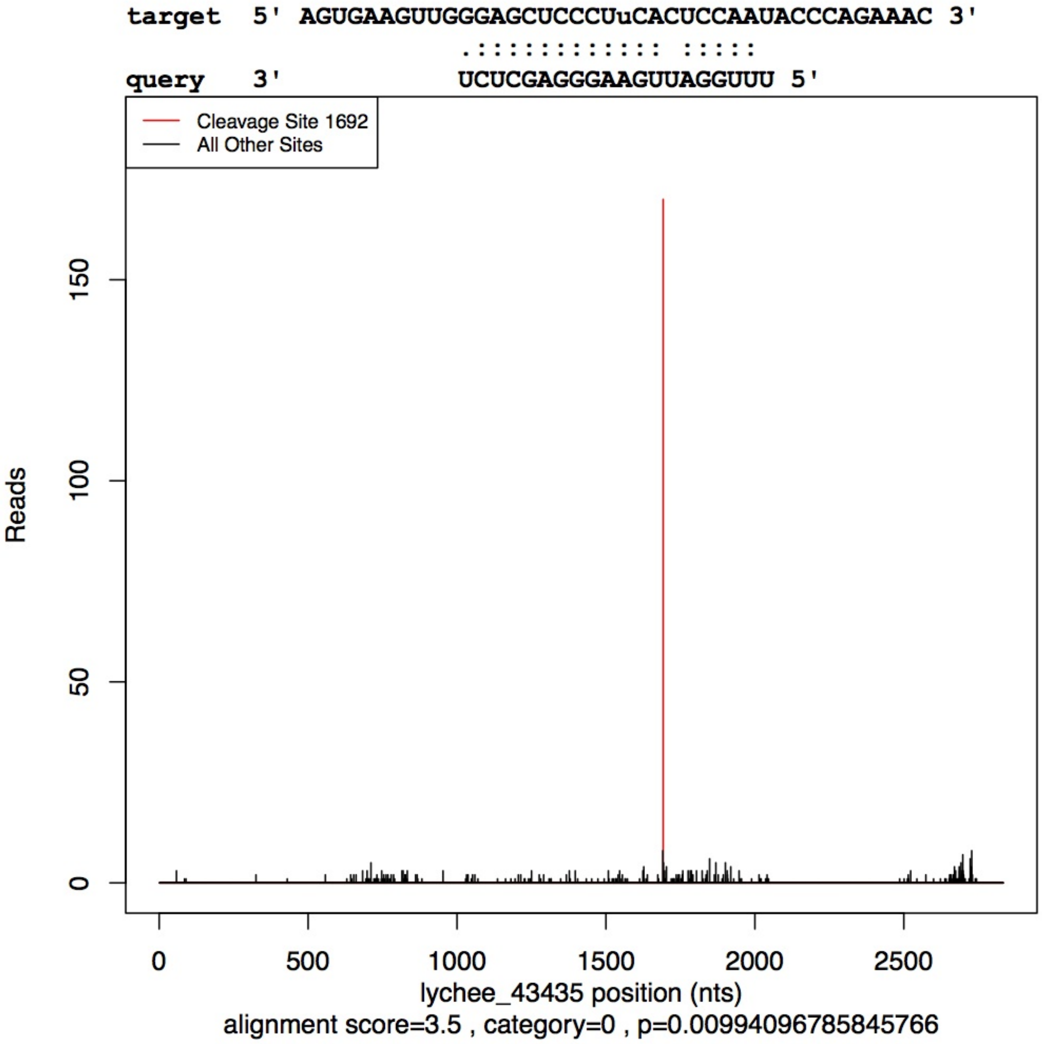

16_miR159_1692_lychee_43435

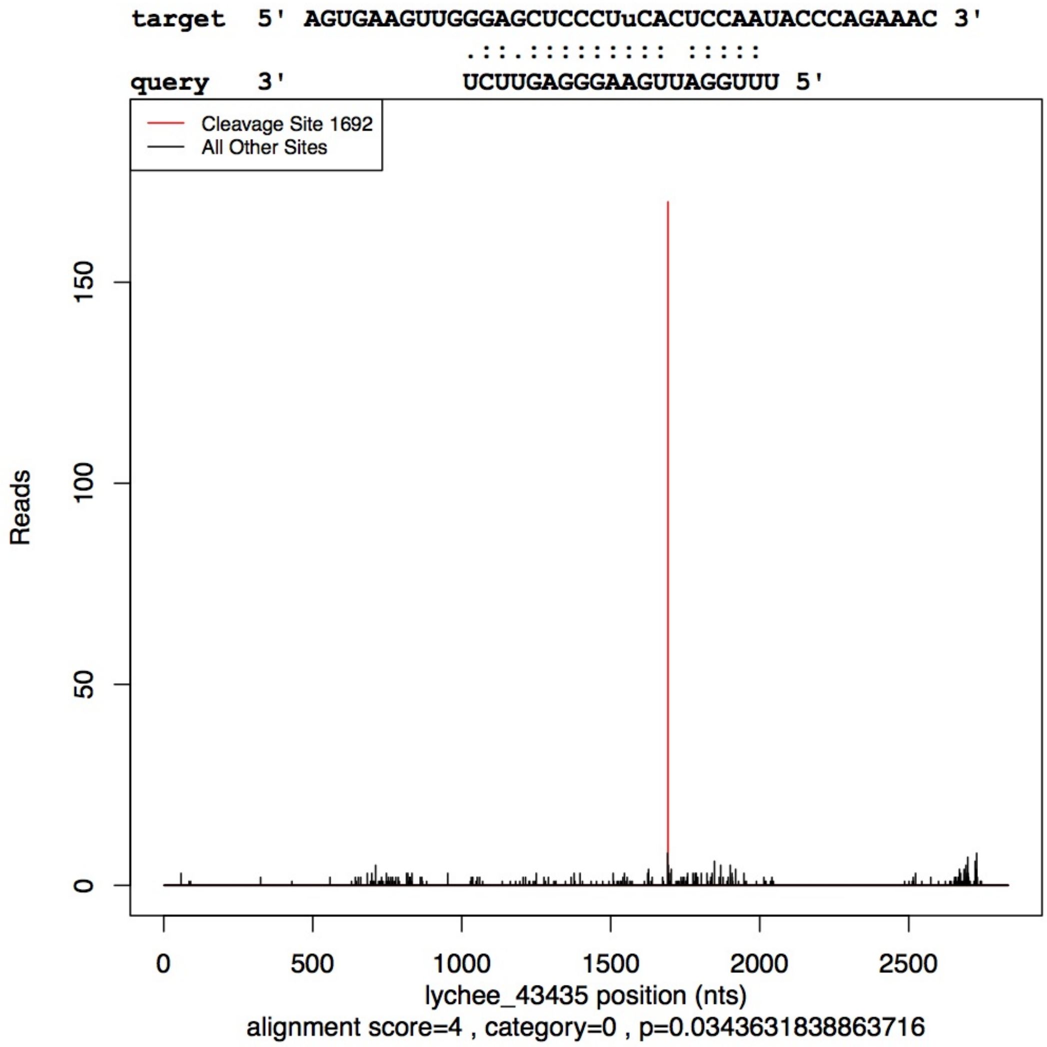

20_miR159_1692_lychee_43435

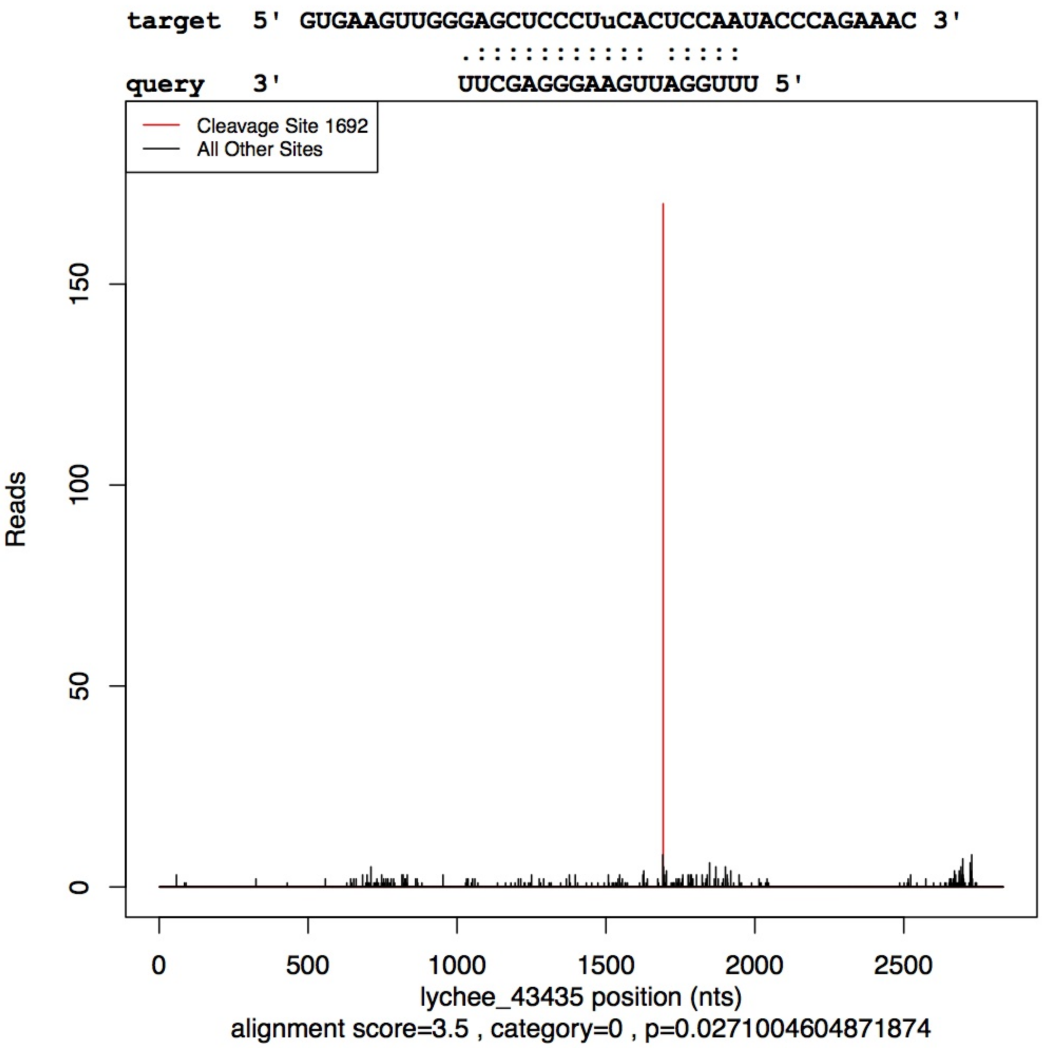

26_miR159_1692_lychee_43435

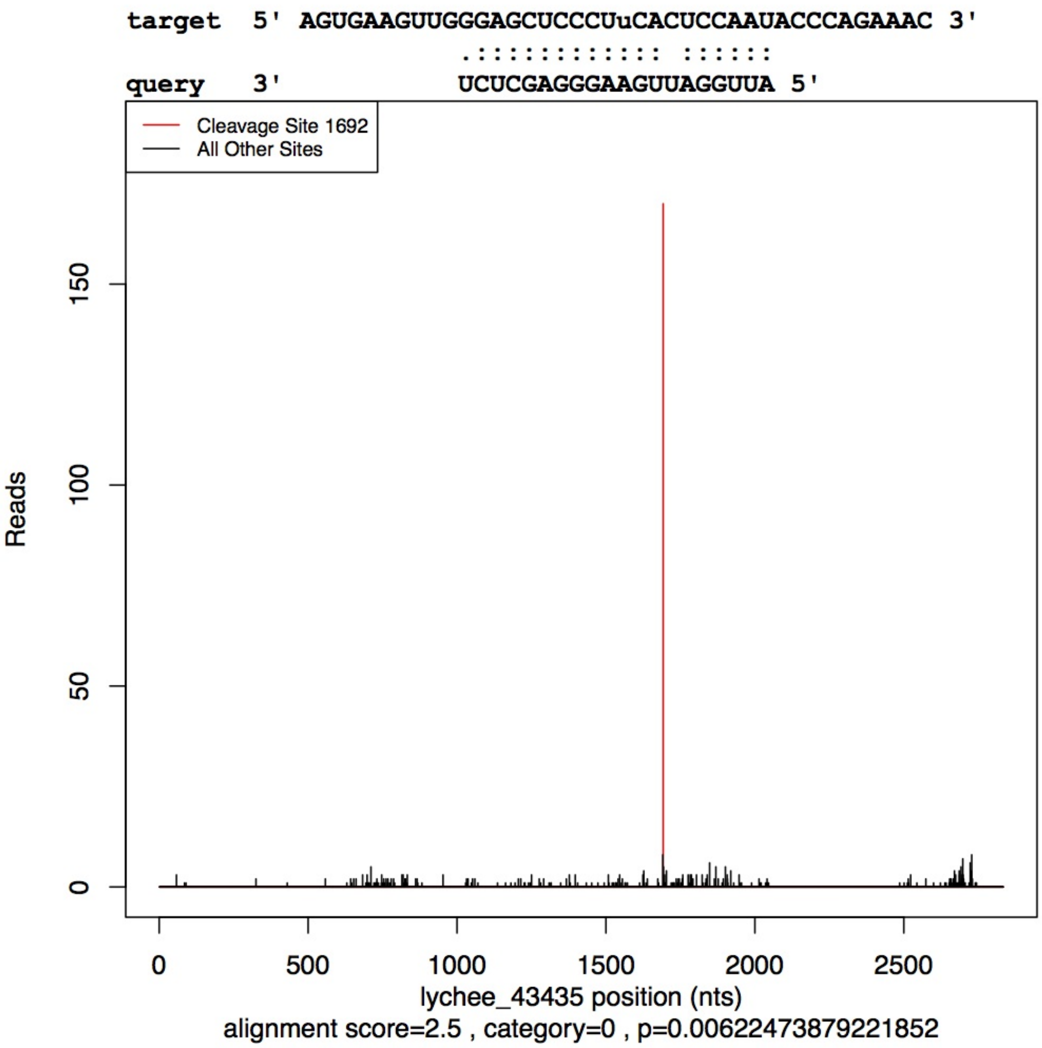

27_miR159_1692_lychee_43435

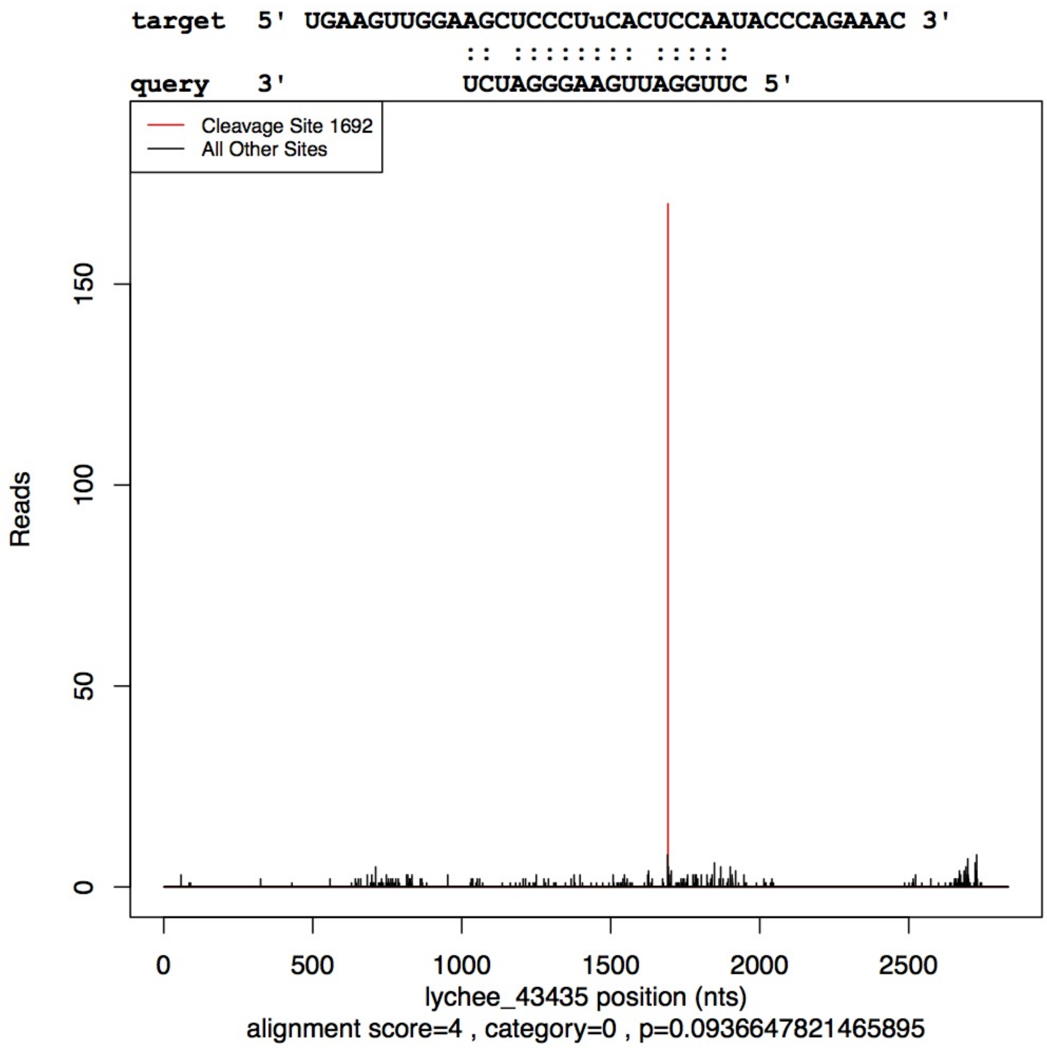

29_miR159_1692_lychee_43435

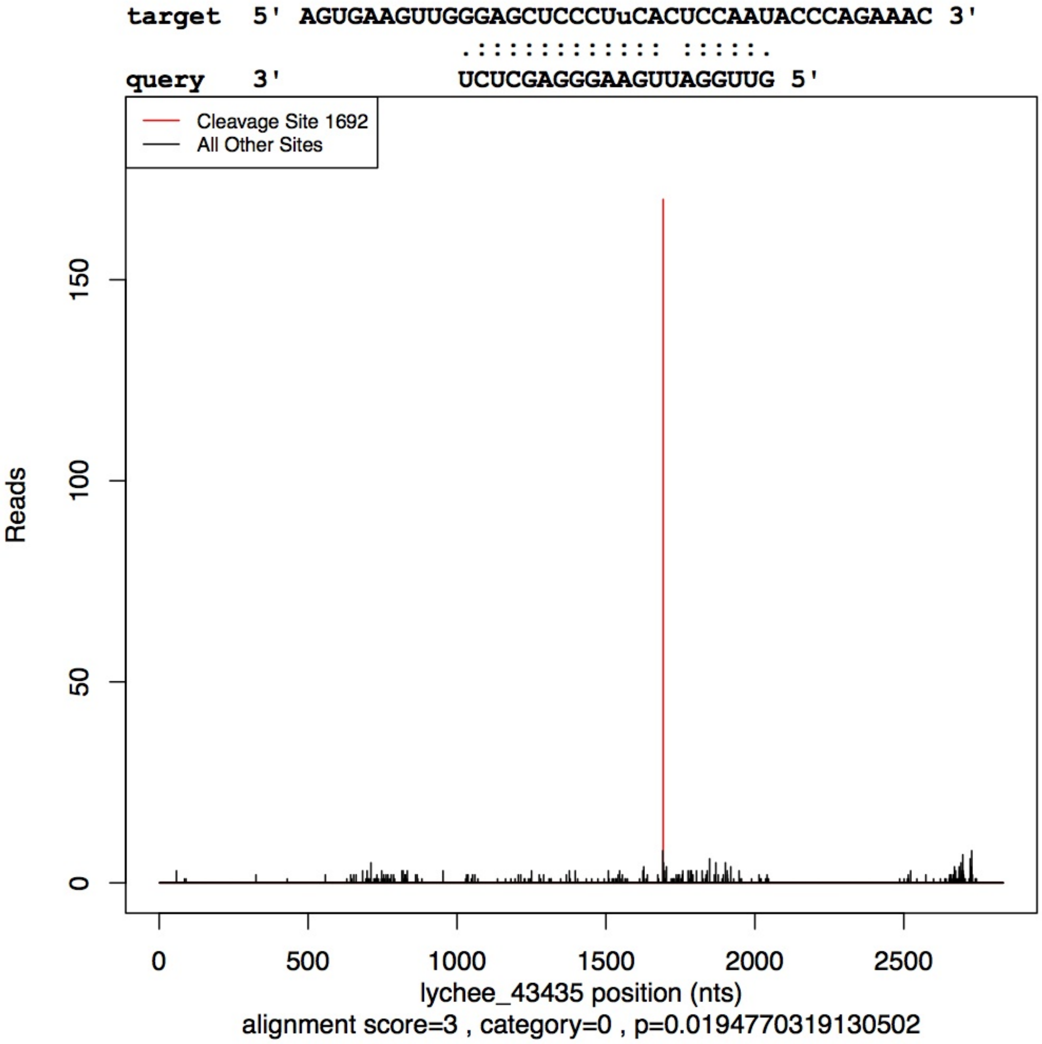

40_miR159_1692_lychee_43435

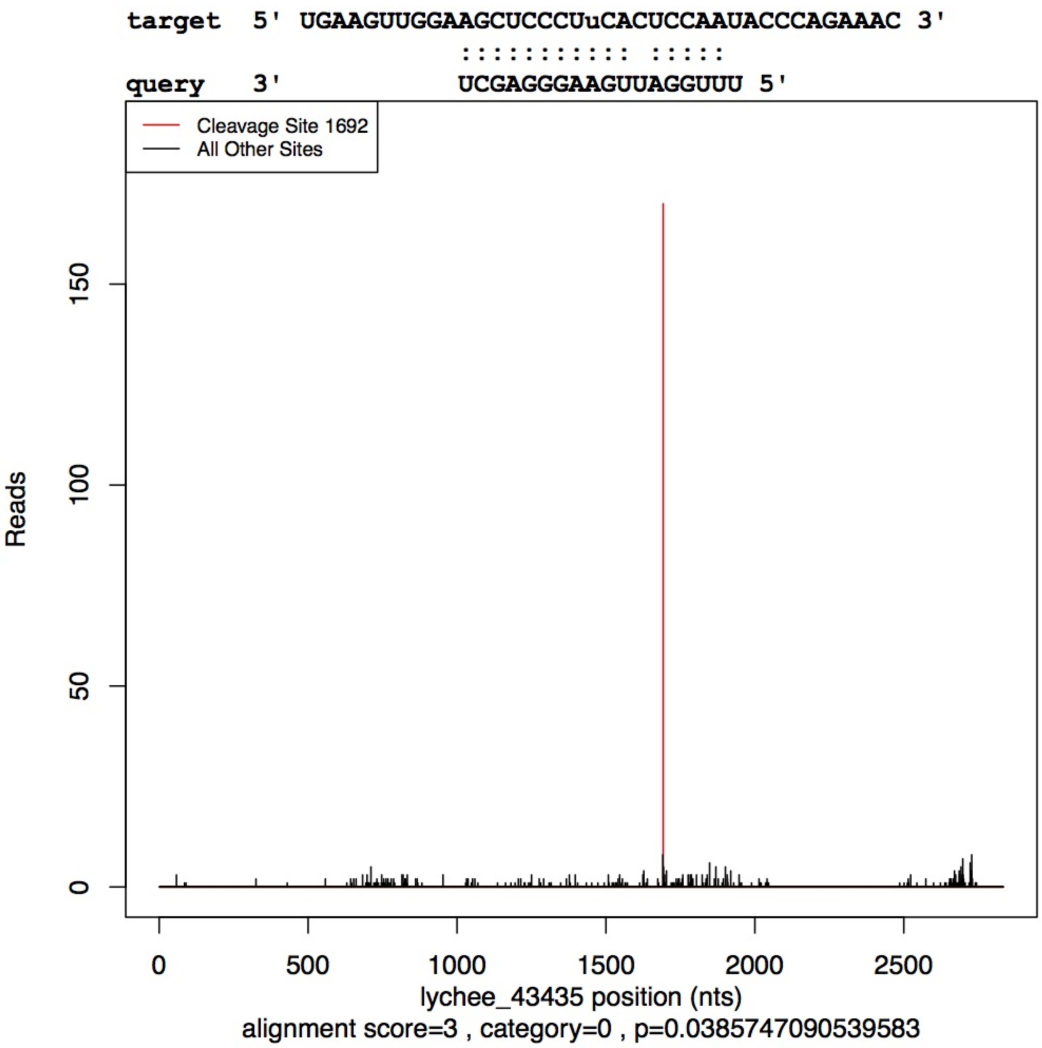

41_miR159_1692_lychee_43435

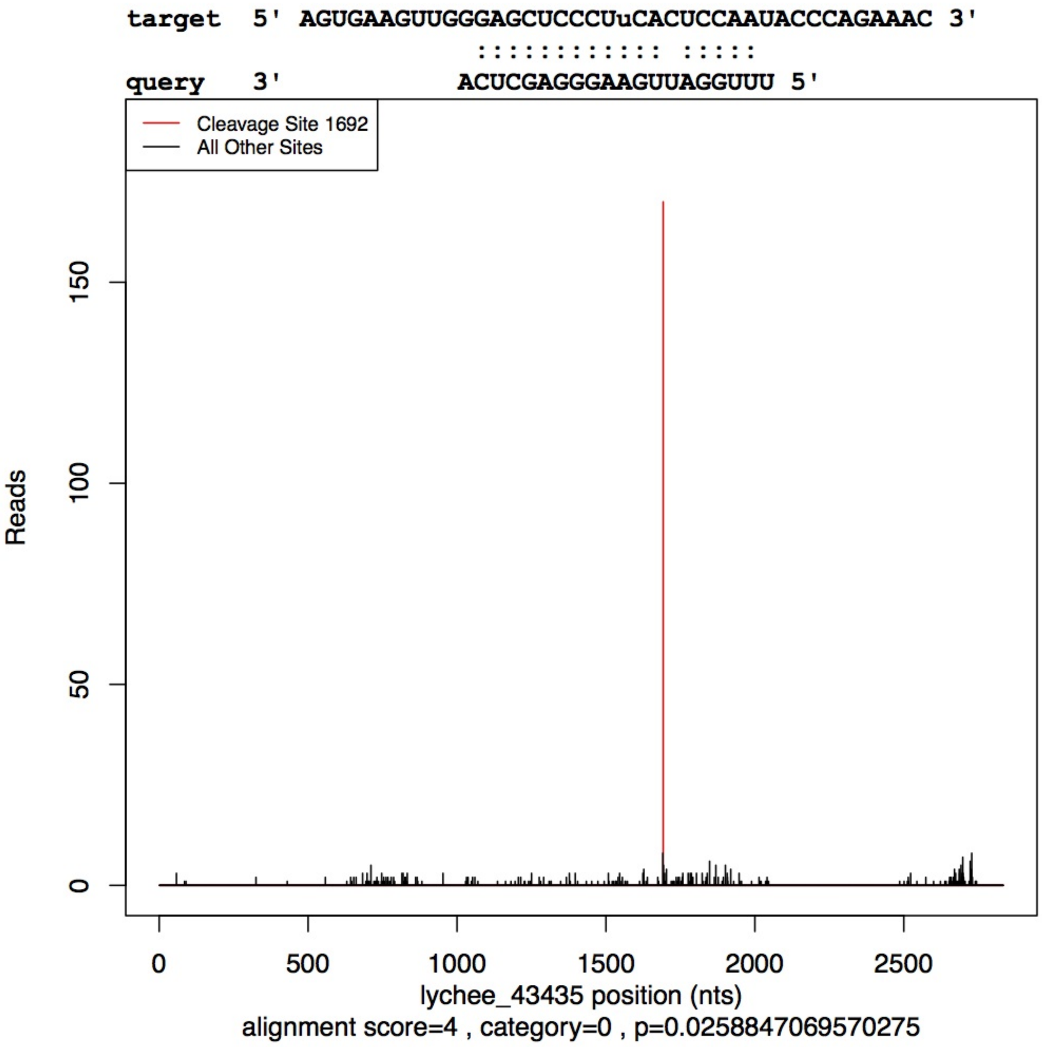

42_miR159_1692_lychee_43435

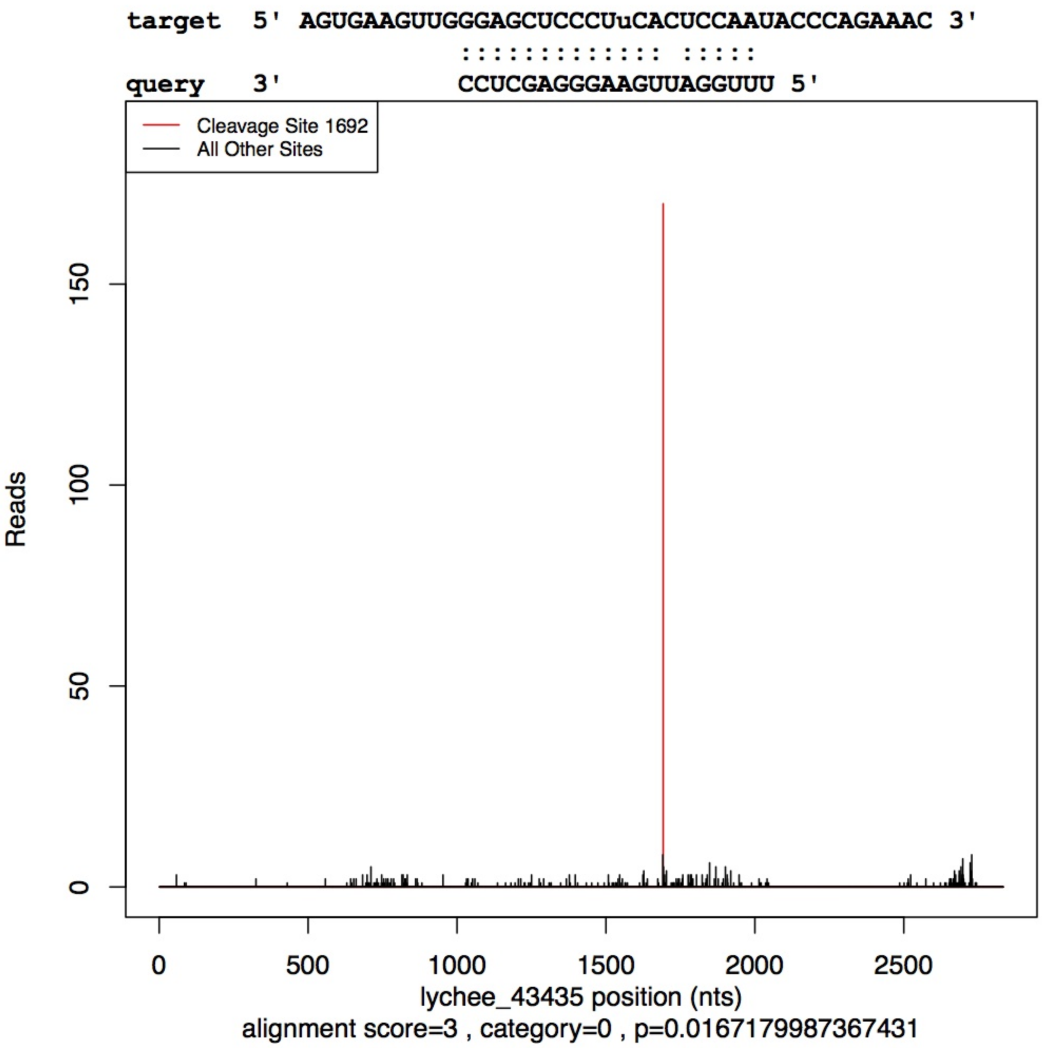

45_miR159_1692_lychee_43435

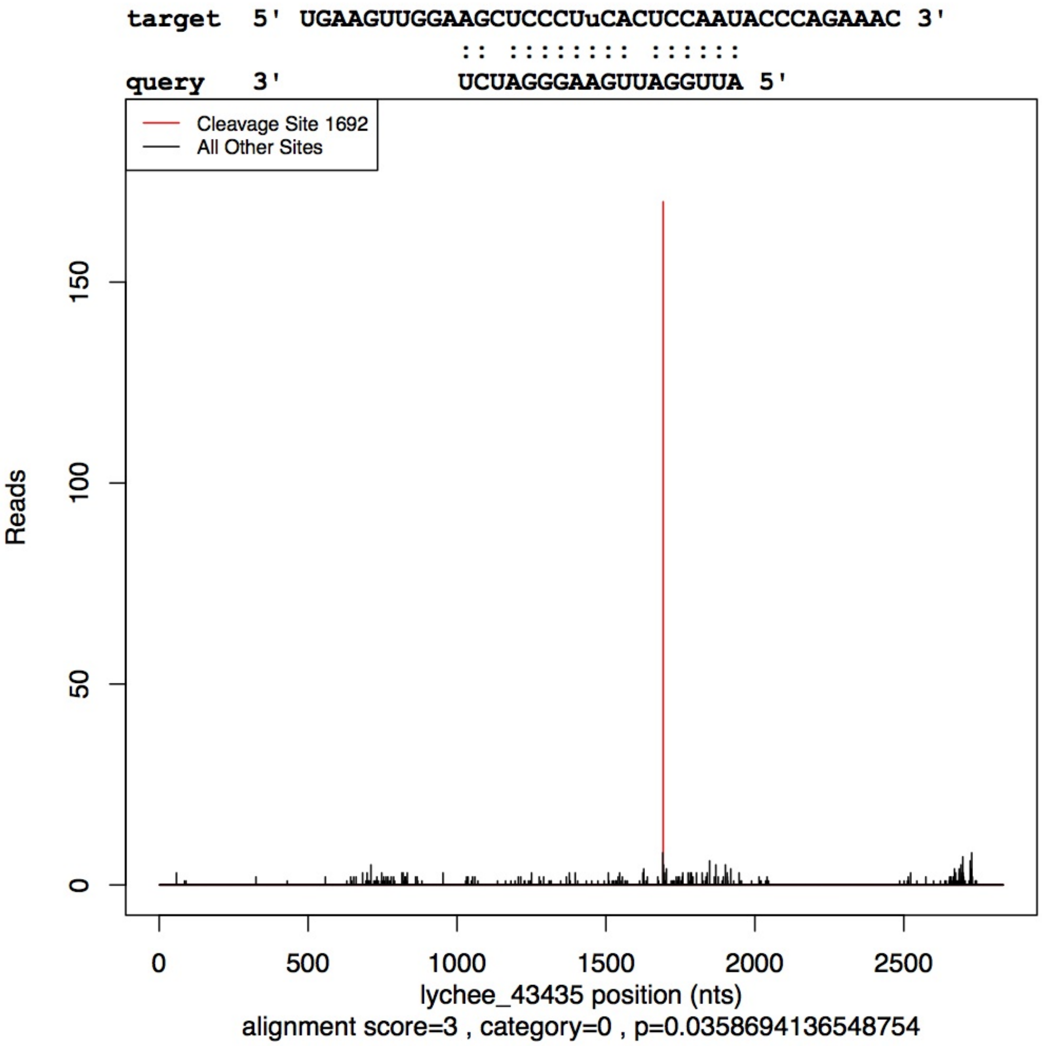

56_miR159_1692_lychee_43435

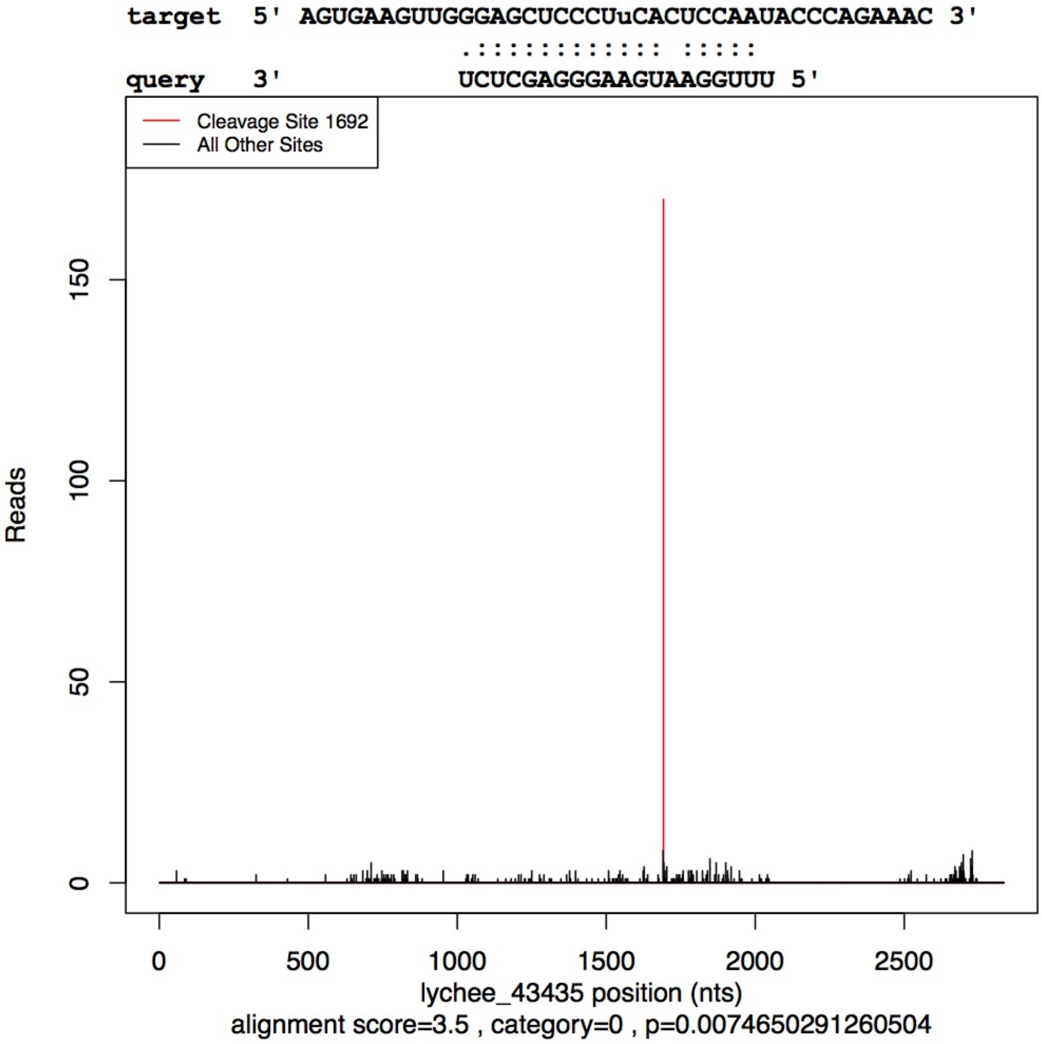

57_miR159_1692_lychee_43435

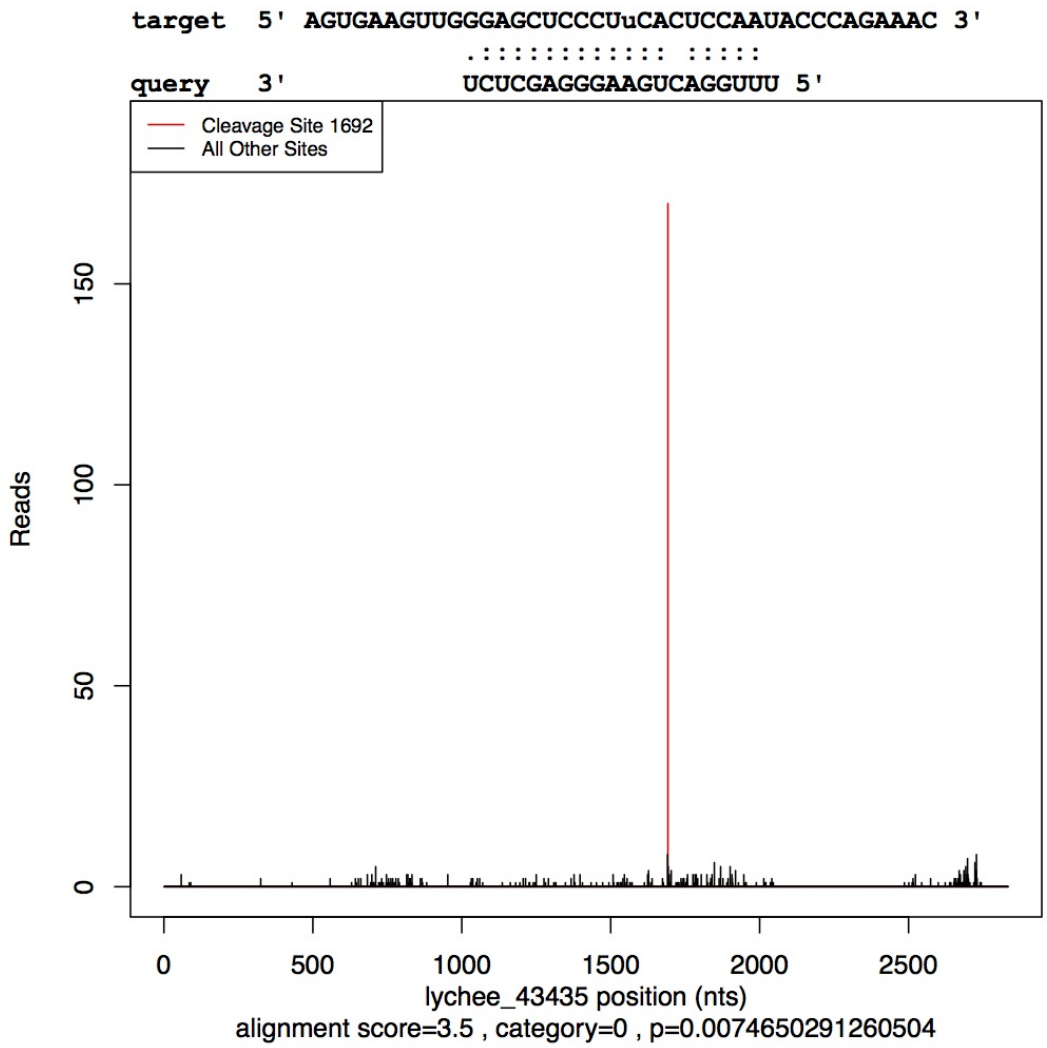

65_miR159_1692_lychee_43435

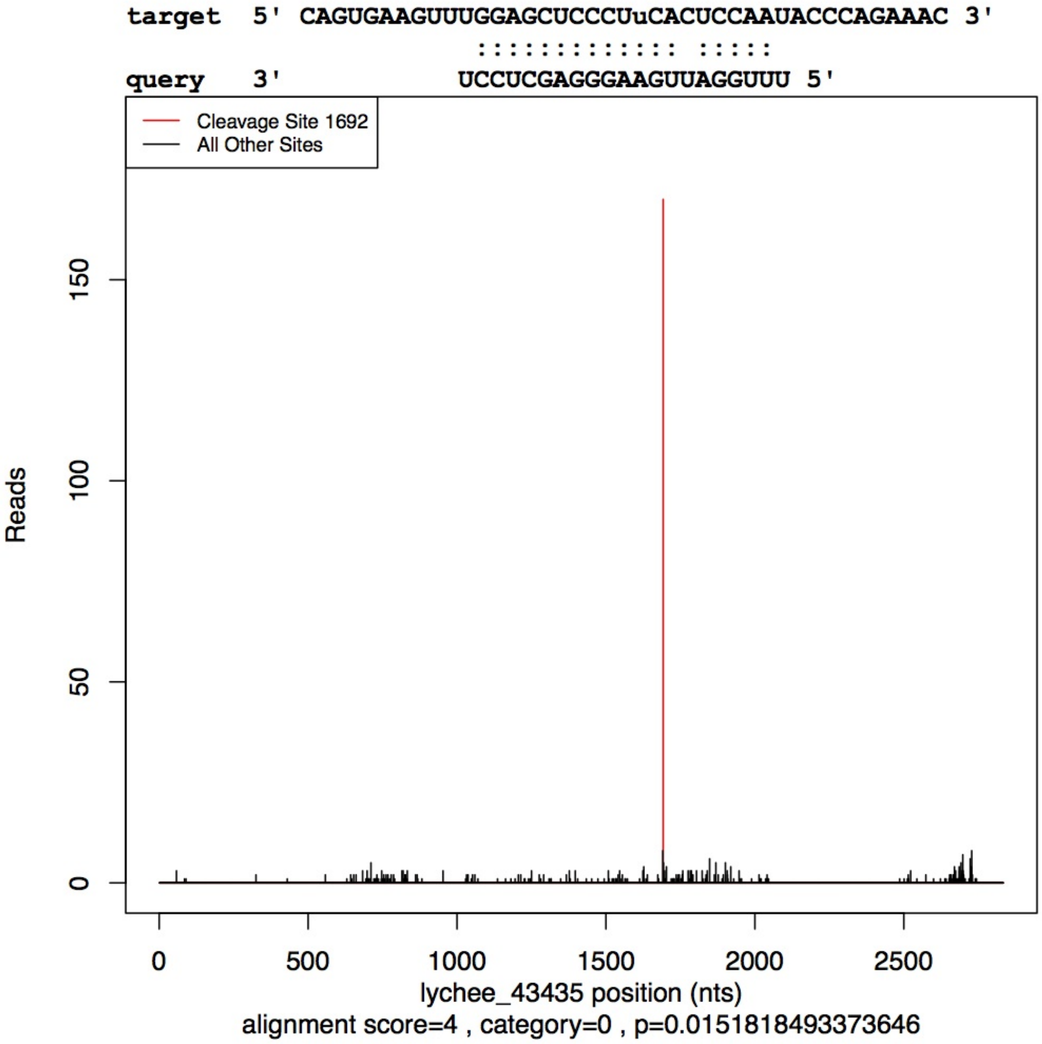

66_miR159_1692_lychee_43435

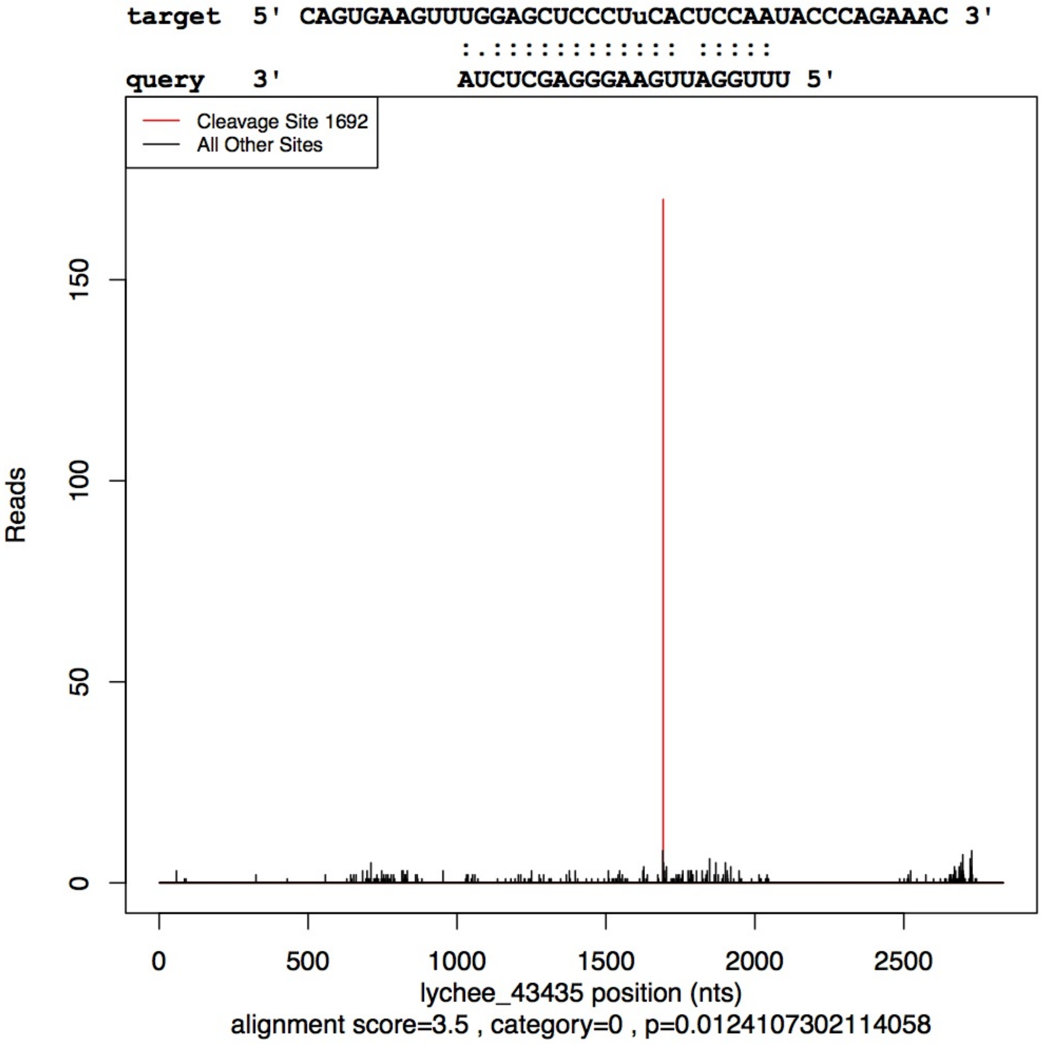

70_miR159_1692_lychee_43435

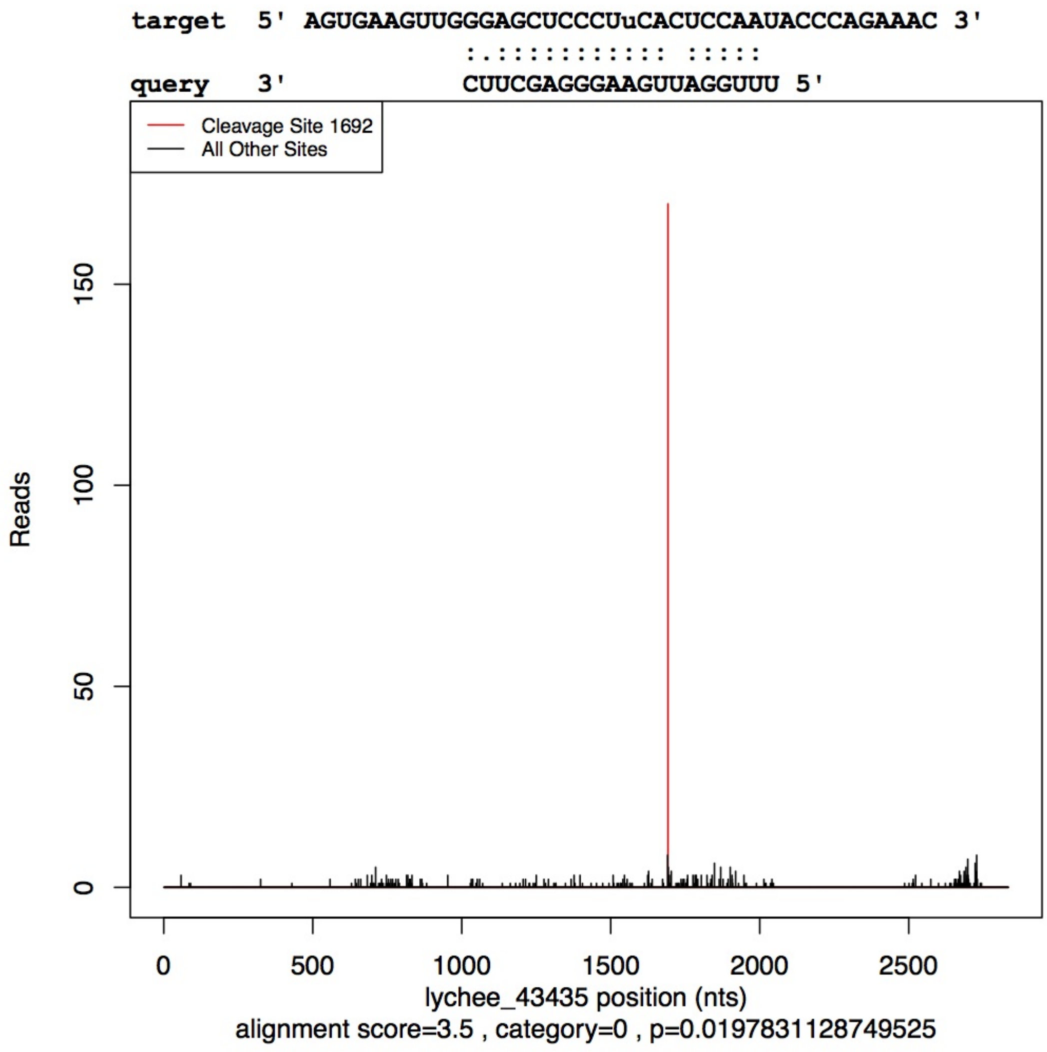

73_miR159_1692_lychee_43435

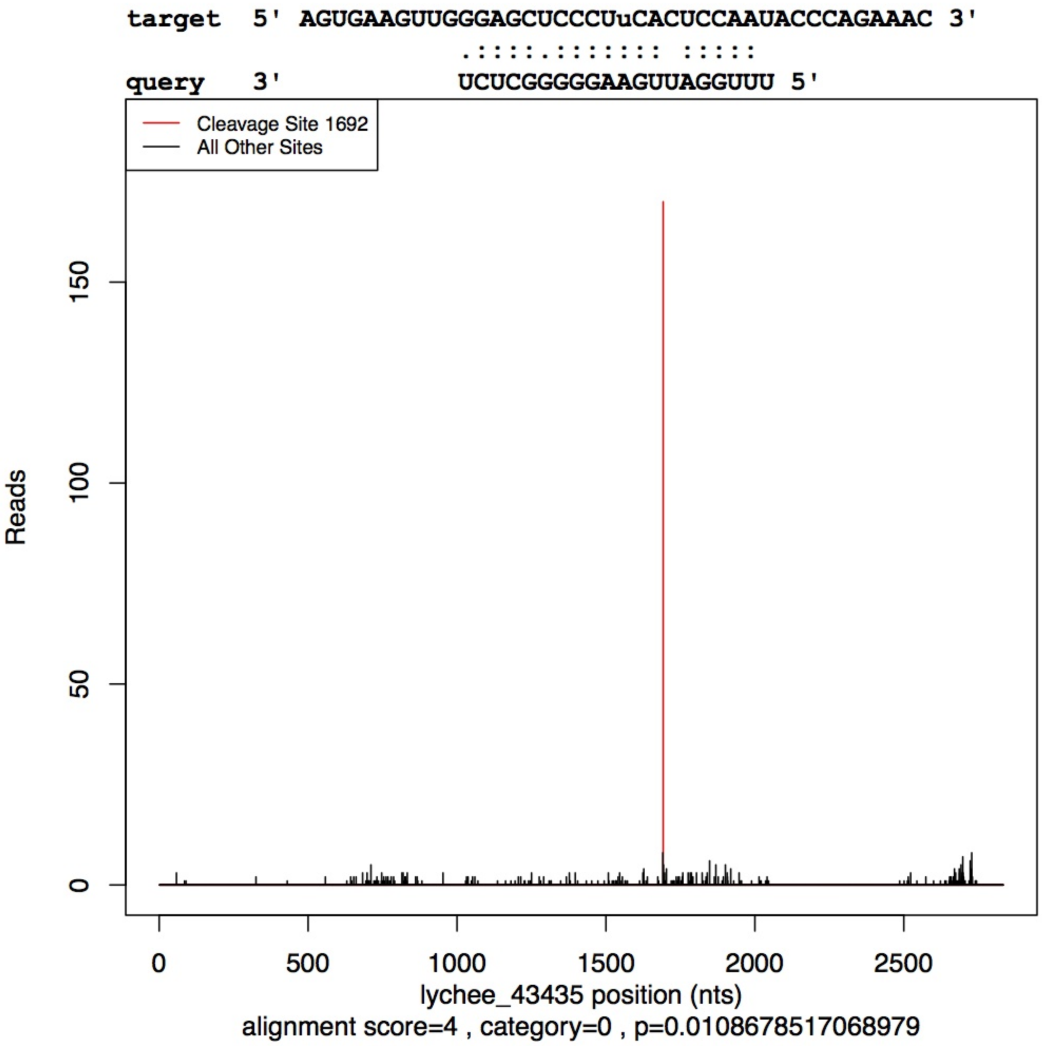

83_miR165_11_lychee_12179

```
target   5' UGAAUAAGGAUGAAGcCUGGUCCGGAUUCGGUUGG 3'
                           .:::::::::::::::::.
query    3'                UCUACUUCGGACCAGGCU 5'
```

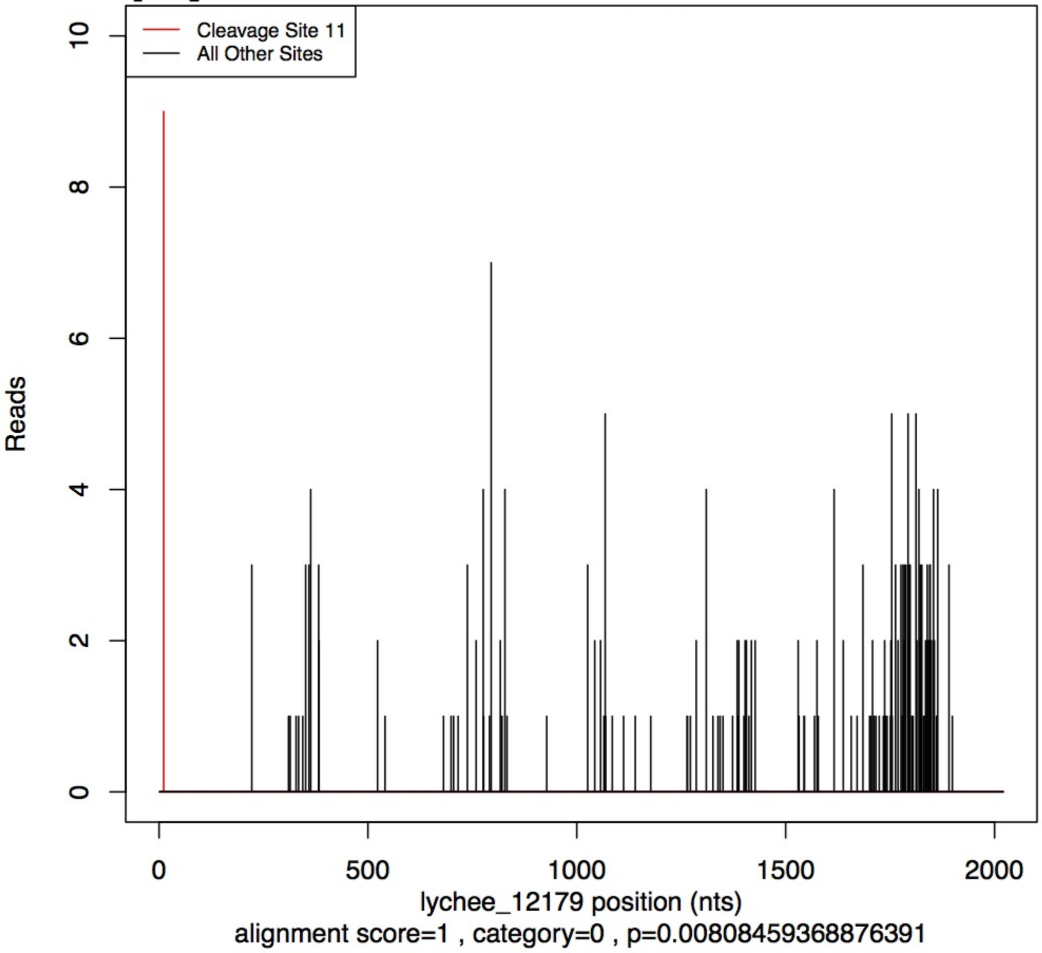

83_miR165_9405_lychee_50219

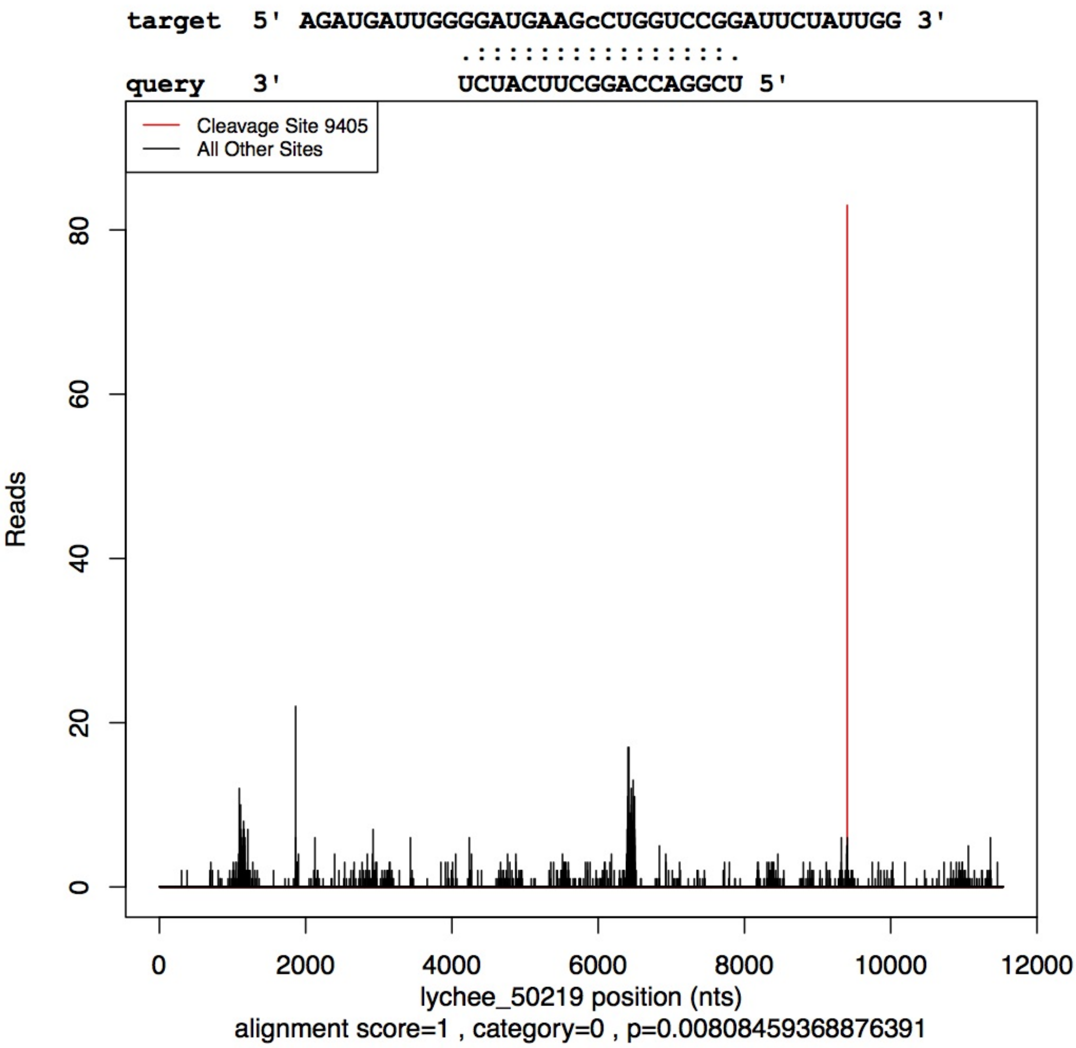

97_miR166_11_lychee_12179

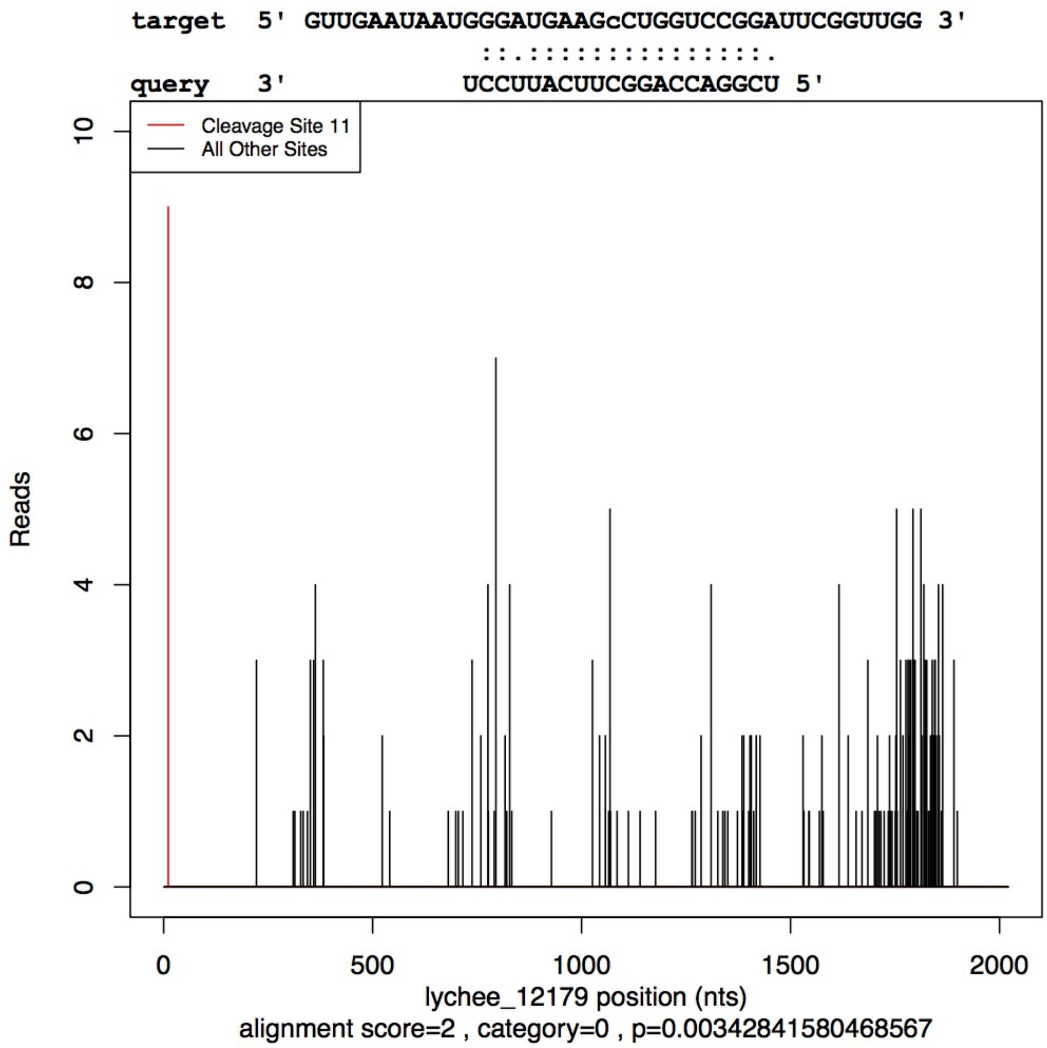

99_miR166_11_lychee_12179

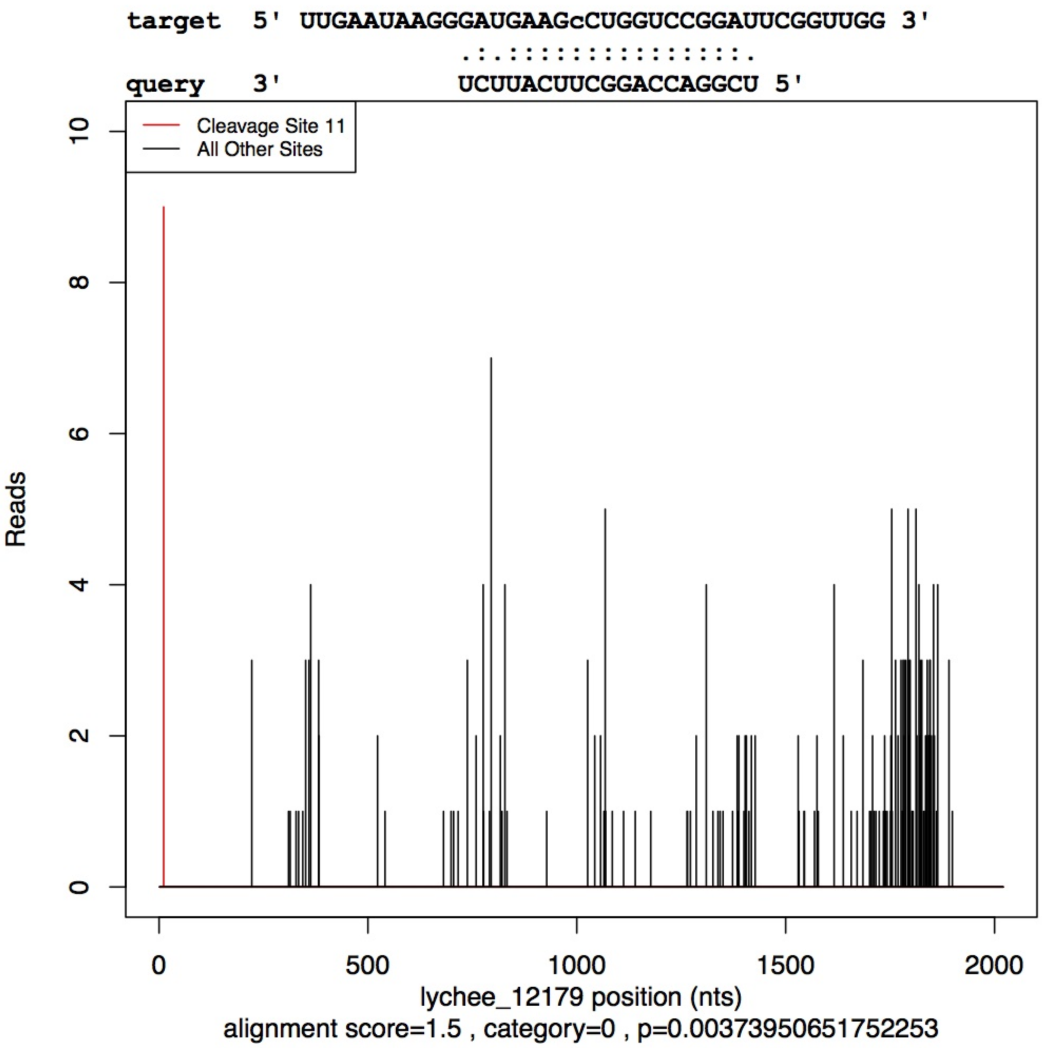

100_miR166_11_lychee_12179

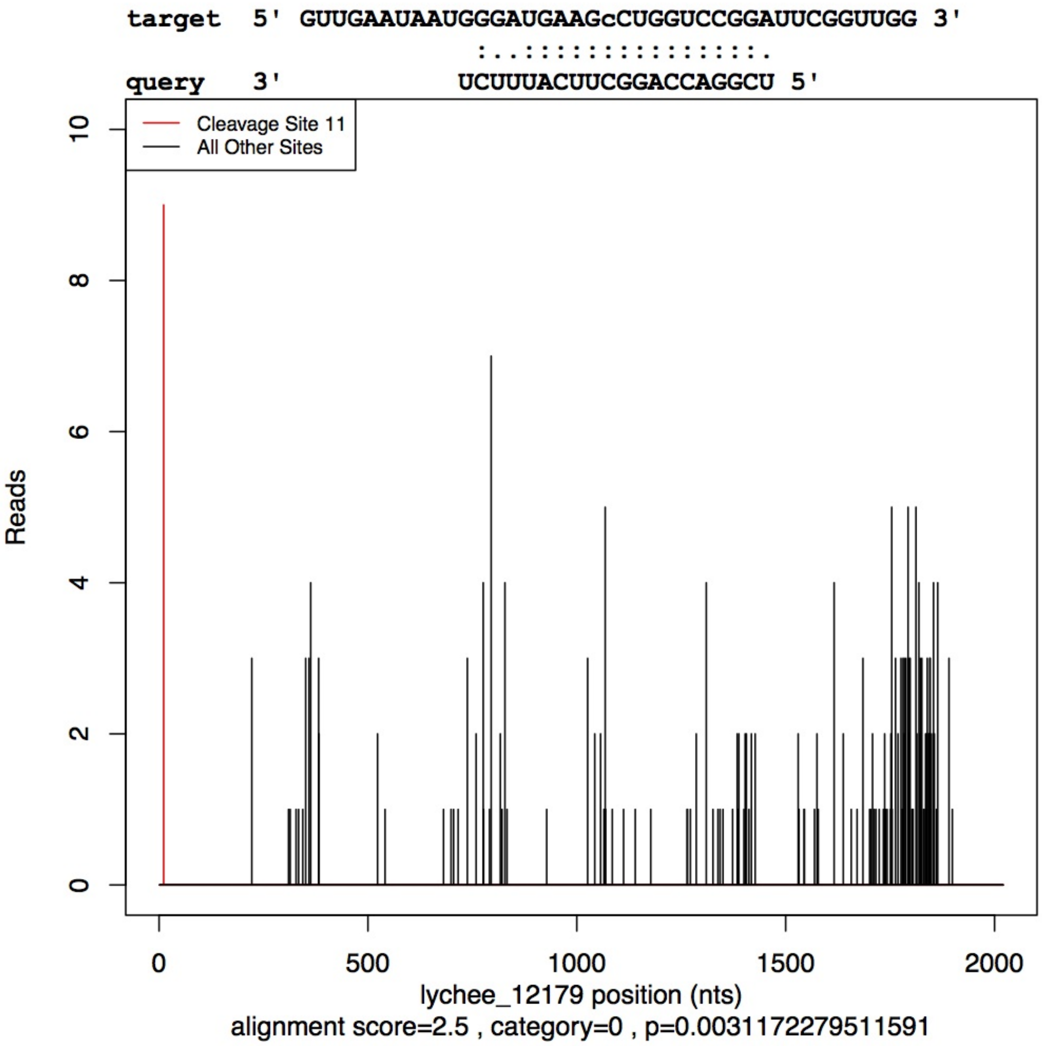

133_miR319_1692_lychee_43435

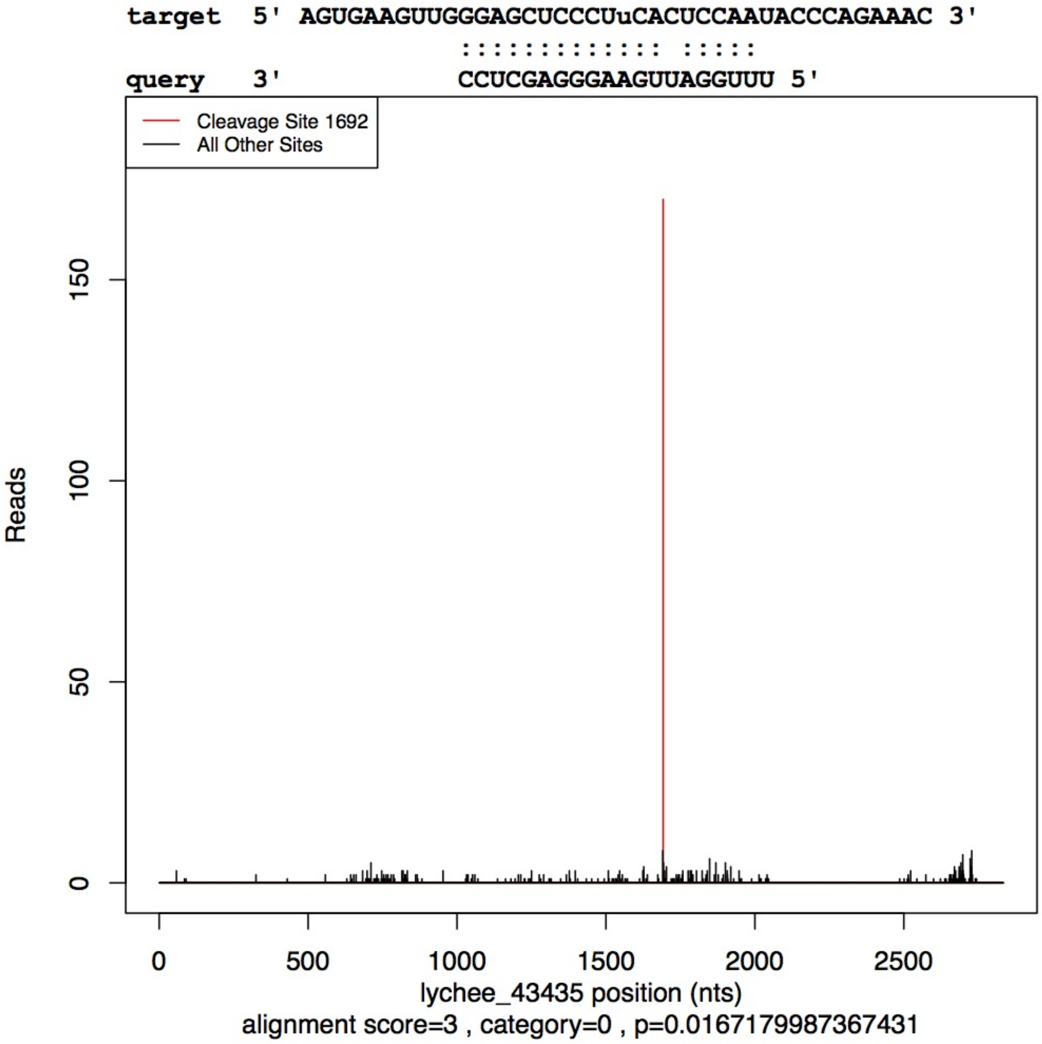

139_miR319_1692_lychee_43435

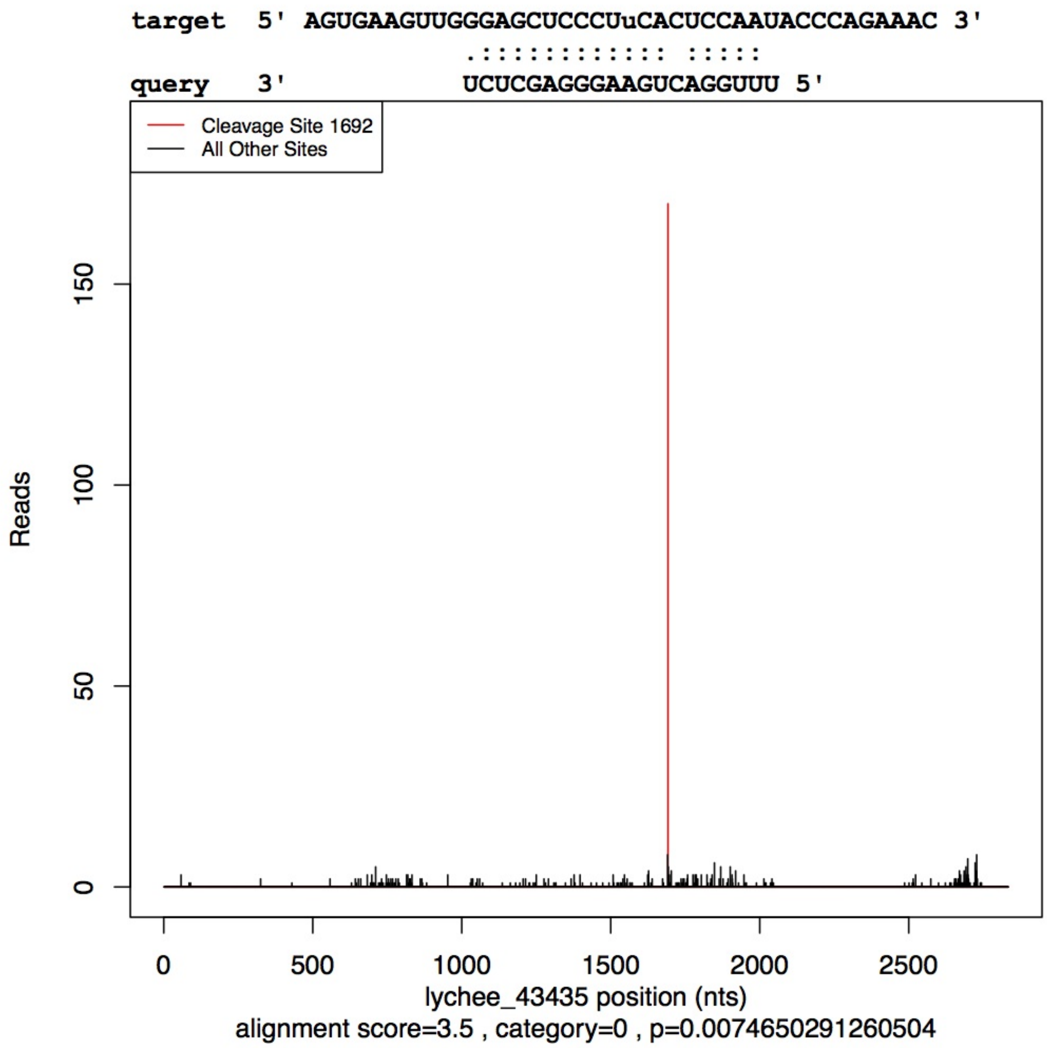

140_miR319_1692_lychee_43435

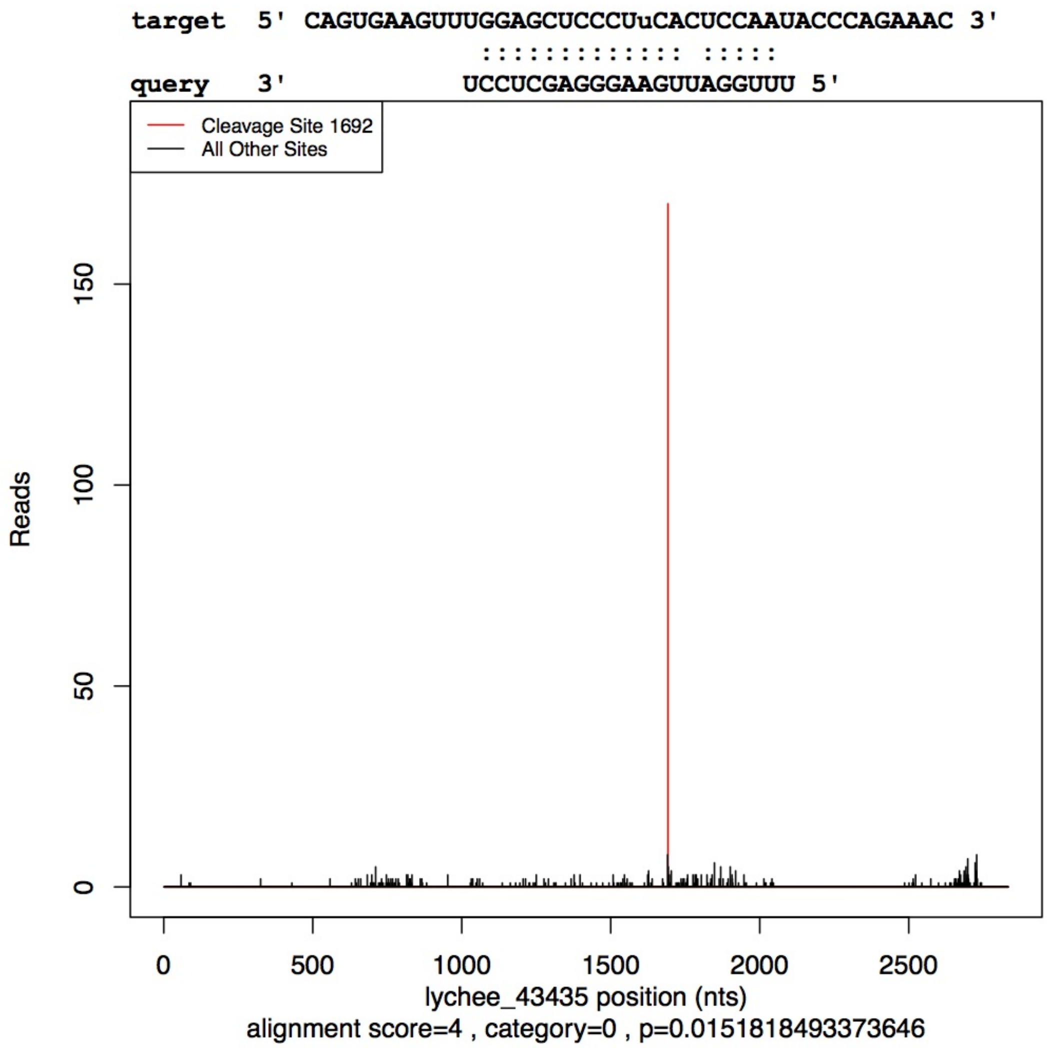

145_miR393_1613_lychee_55456

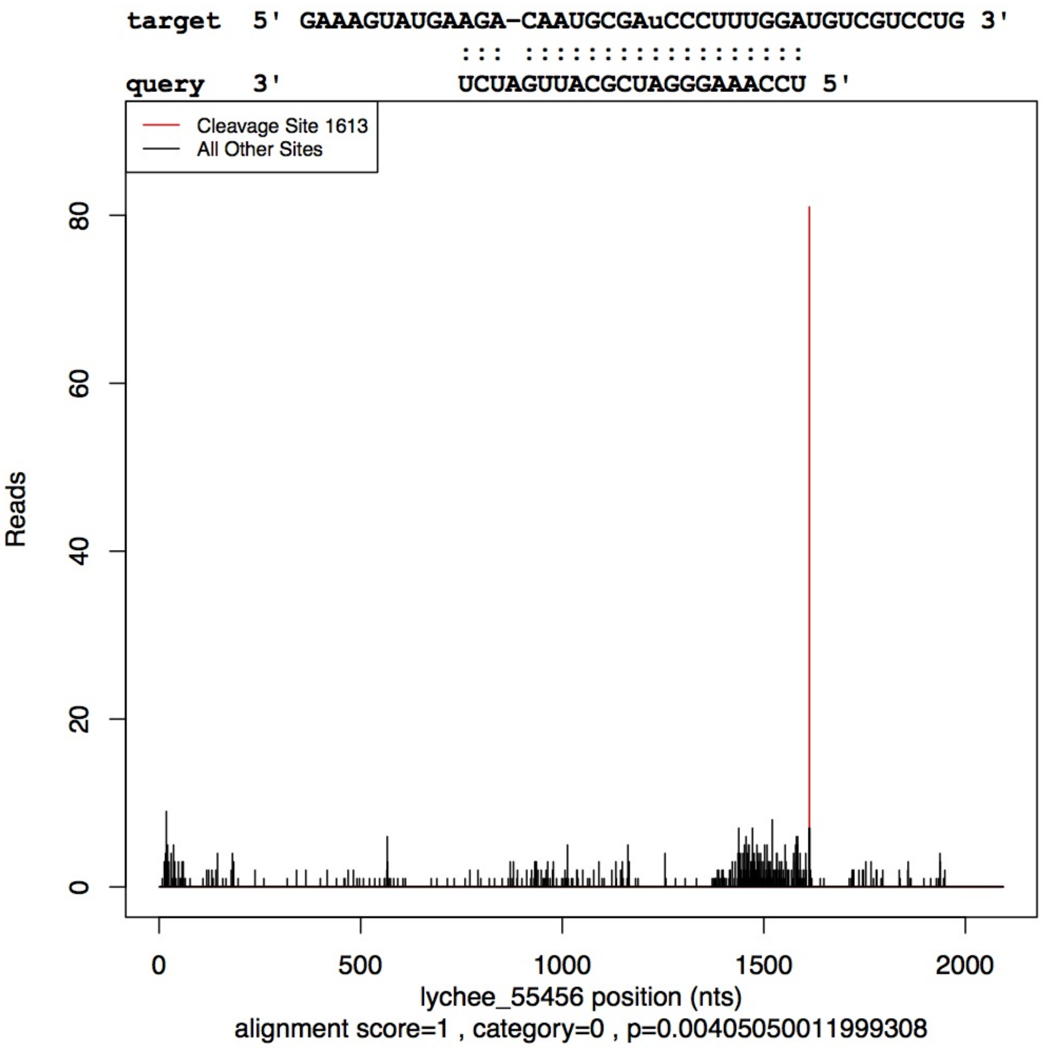

147_miR393_1613_lychee_55456

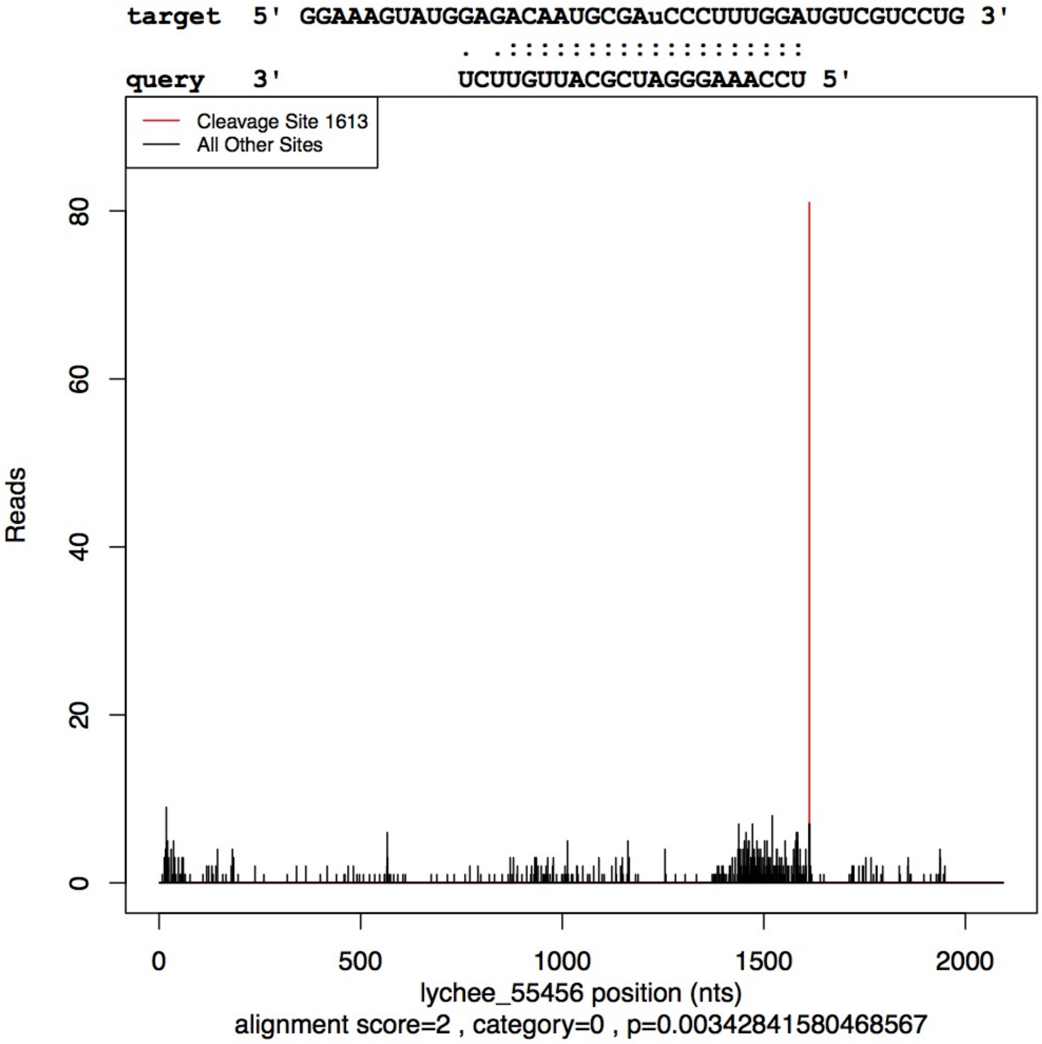

153_miR393_1613_lychee_55456

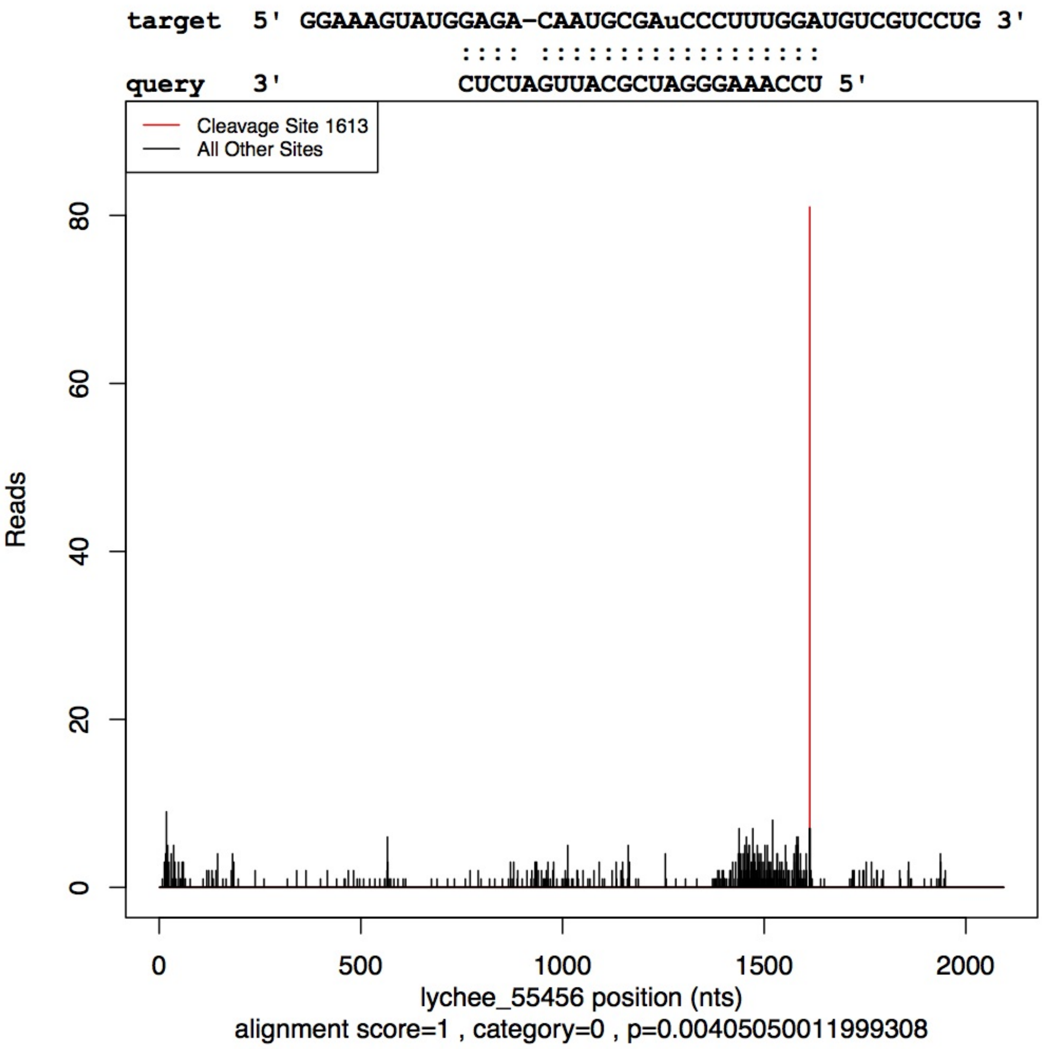

154_miR393_1613_lychee_55456

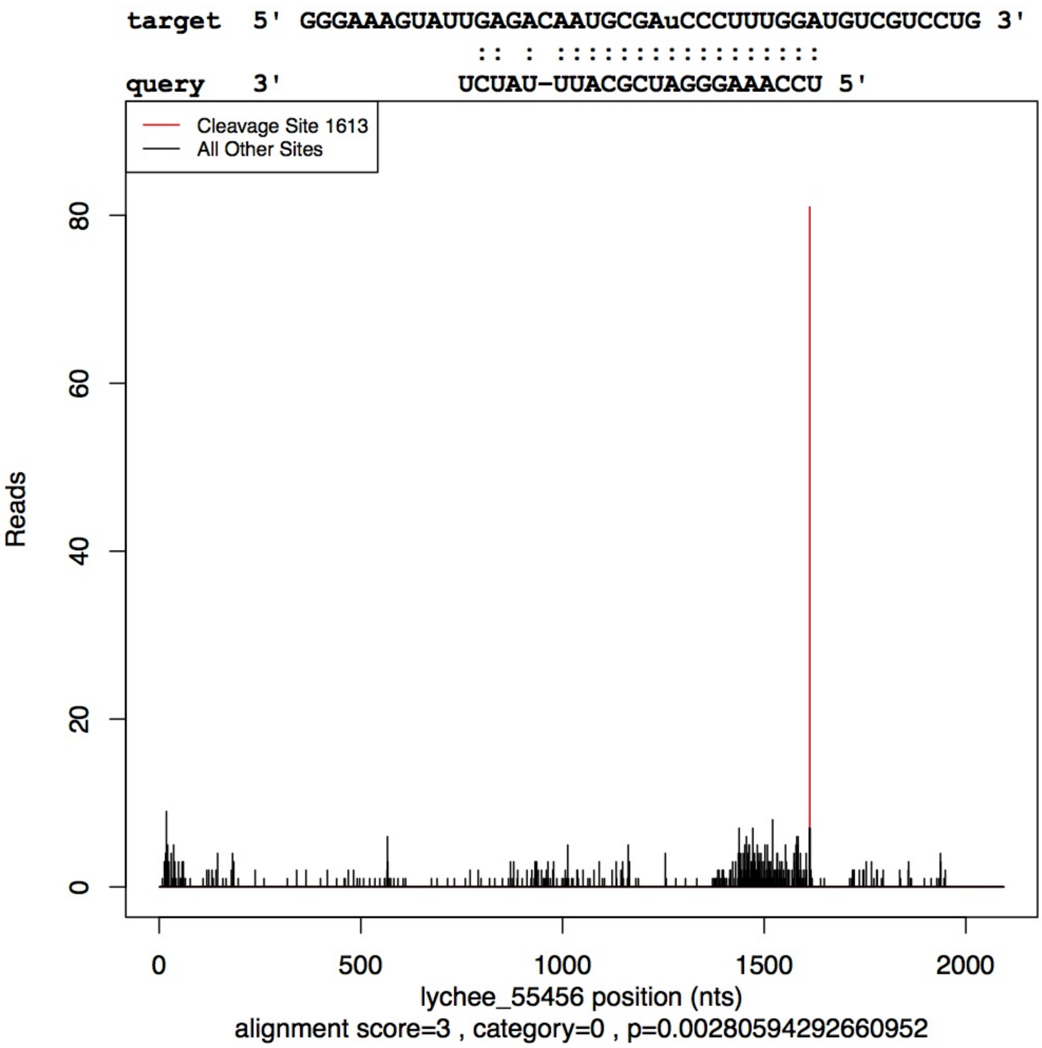

162_miR396_1029_lychee_55389

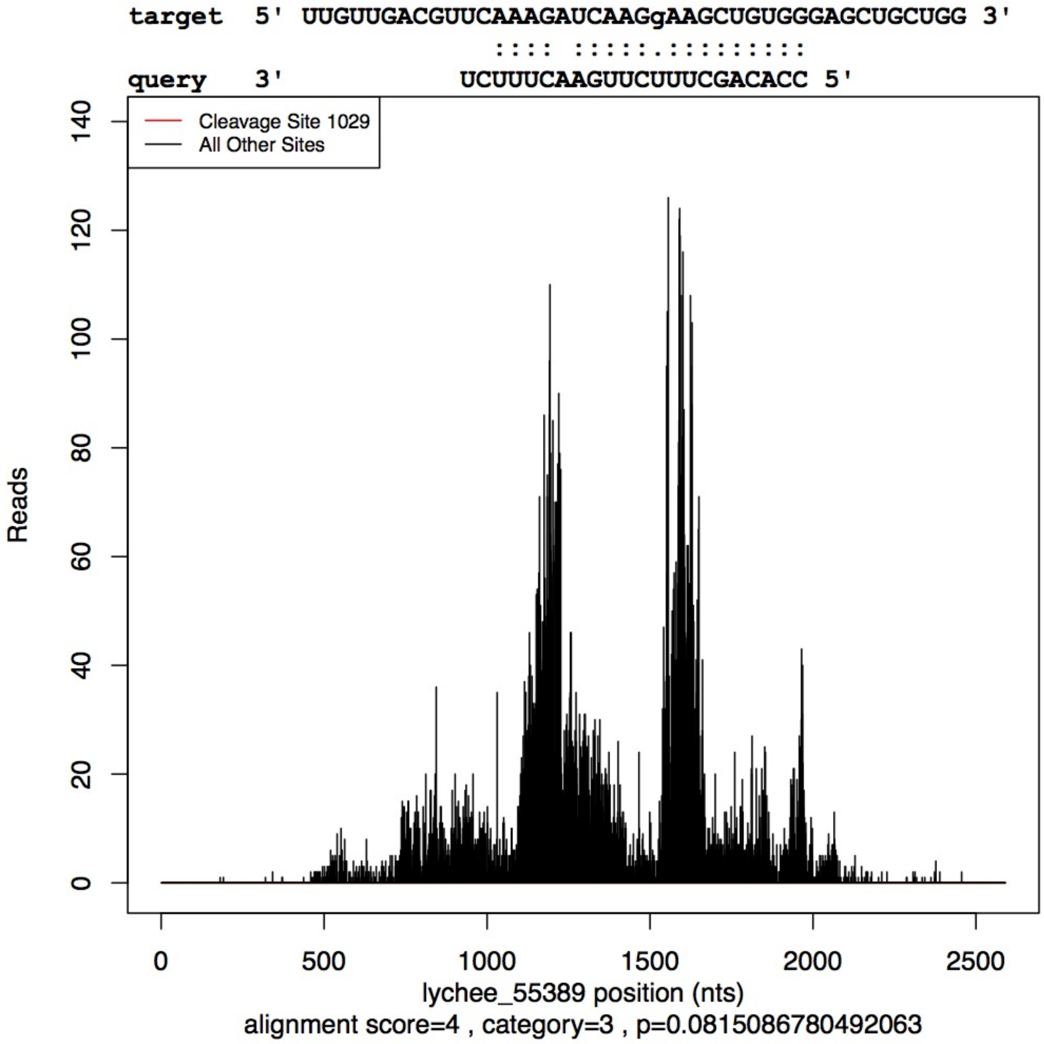

182_miR396_1029_lychee_55389

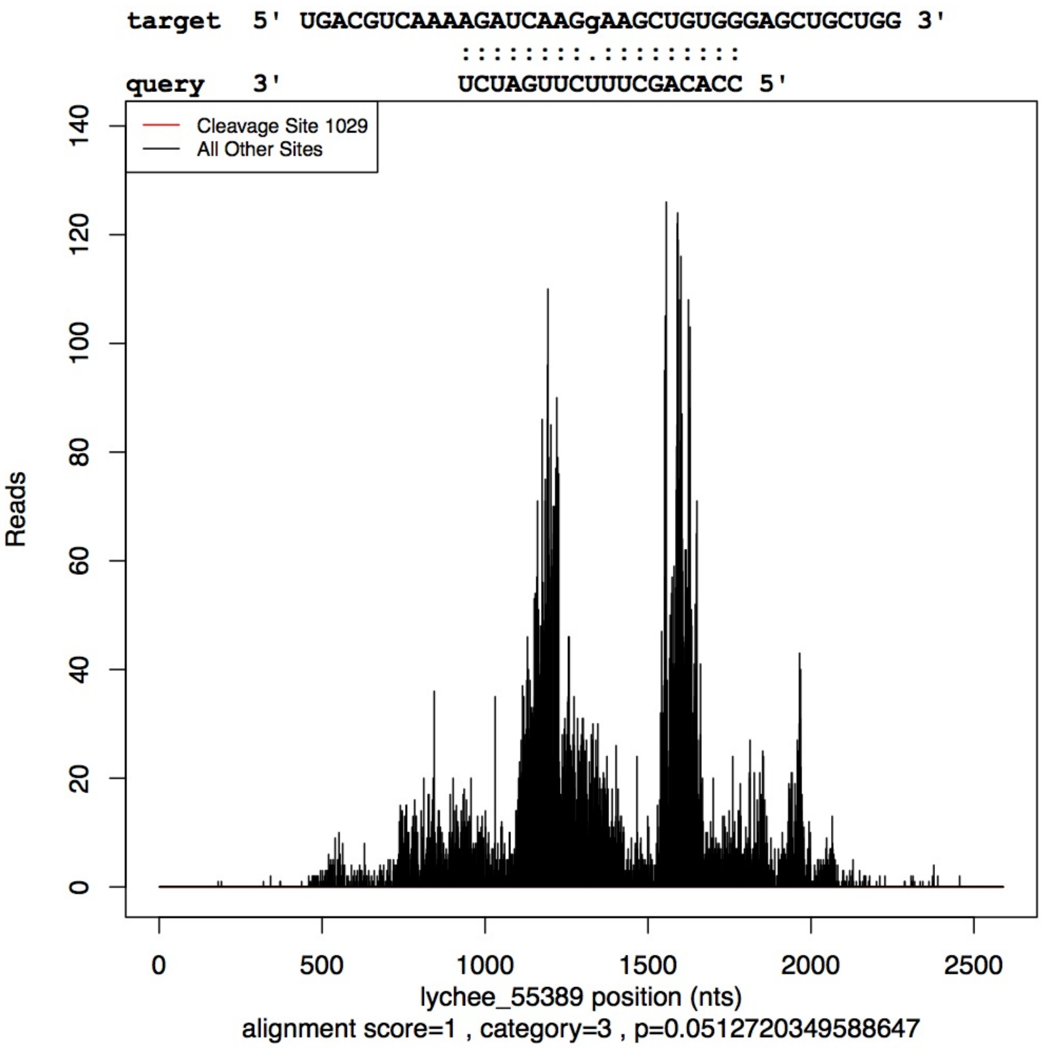

194_miR396_1029_lychee_55389

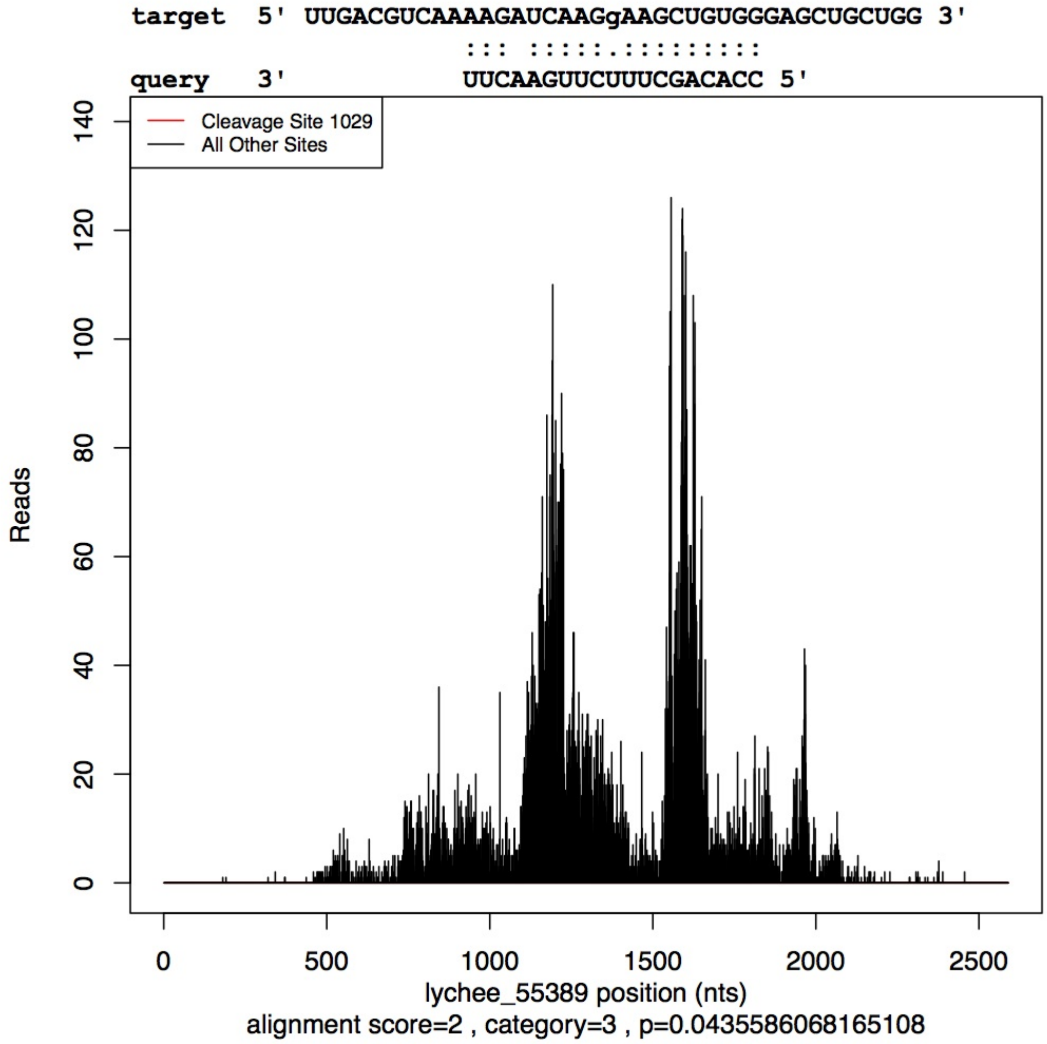

209_miR398_107_lychee_14851

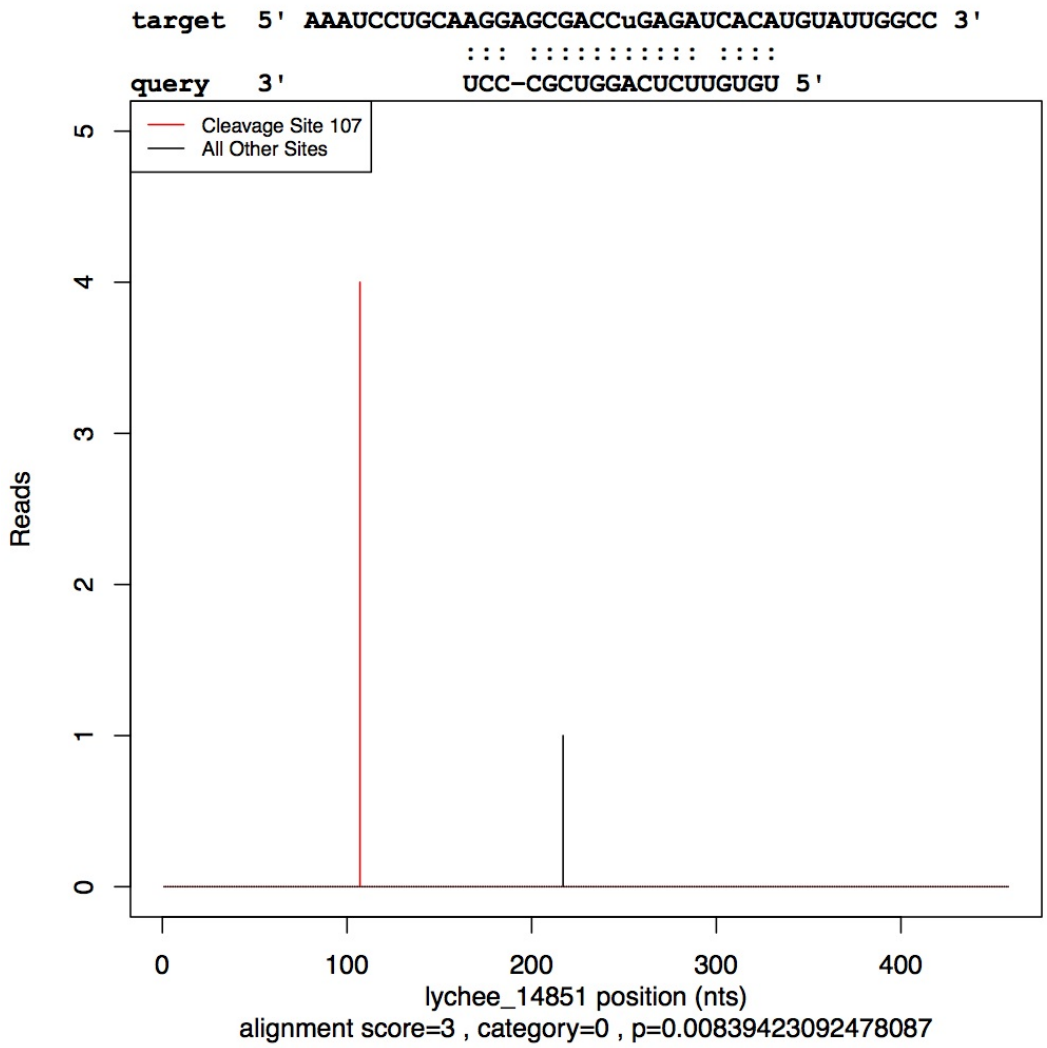

210_miR398_107_lychee_14851

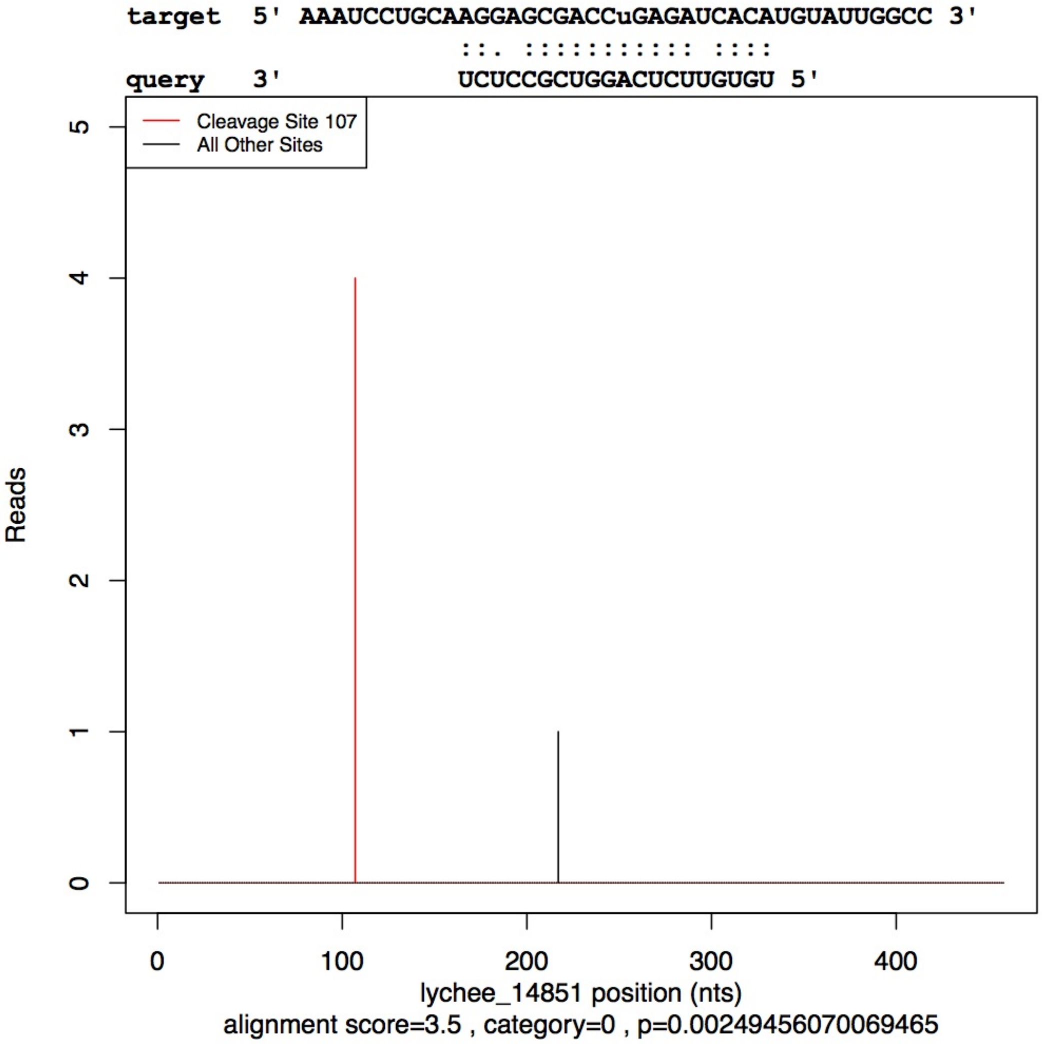

230_miR447_871_lychee_4093

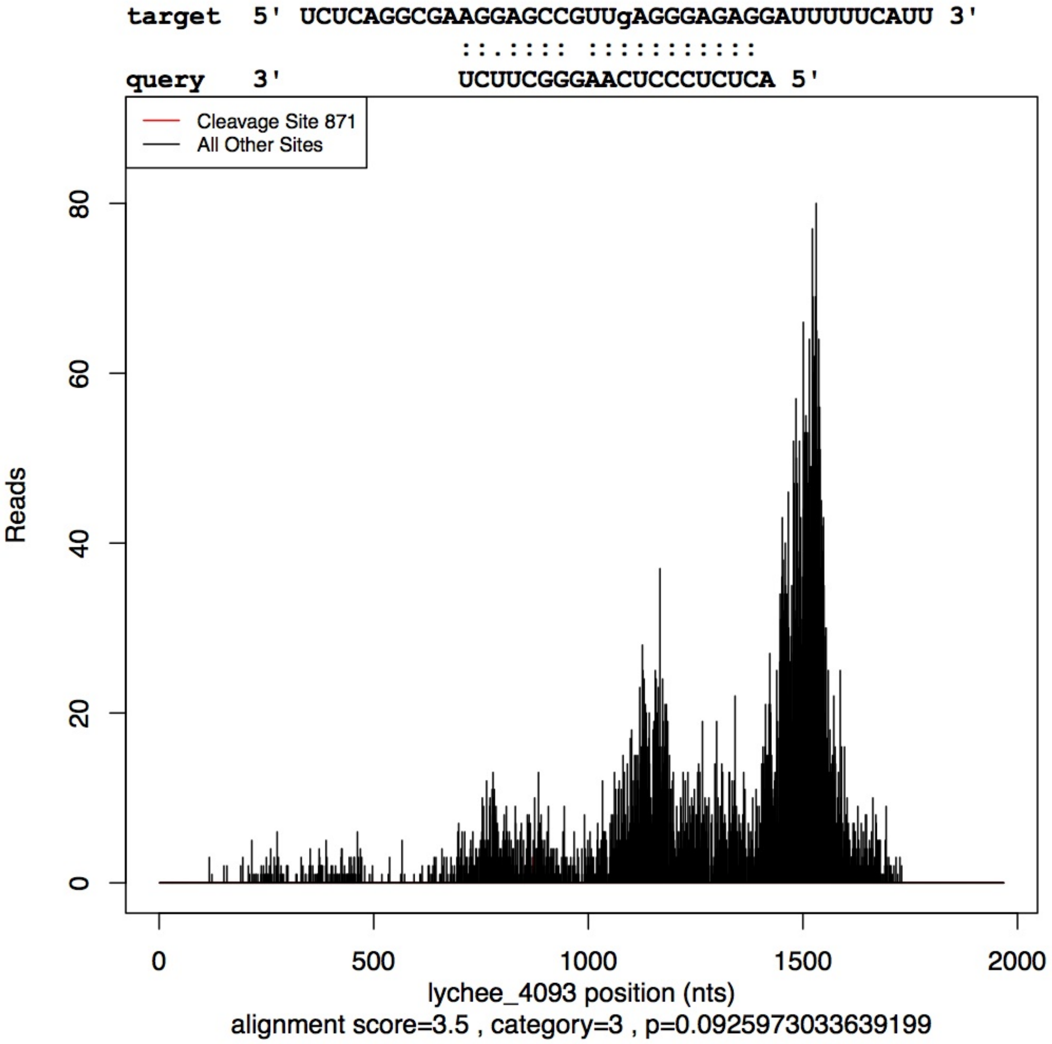

235_miR473_871_lychee_4093

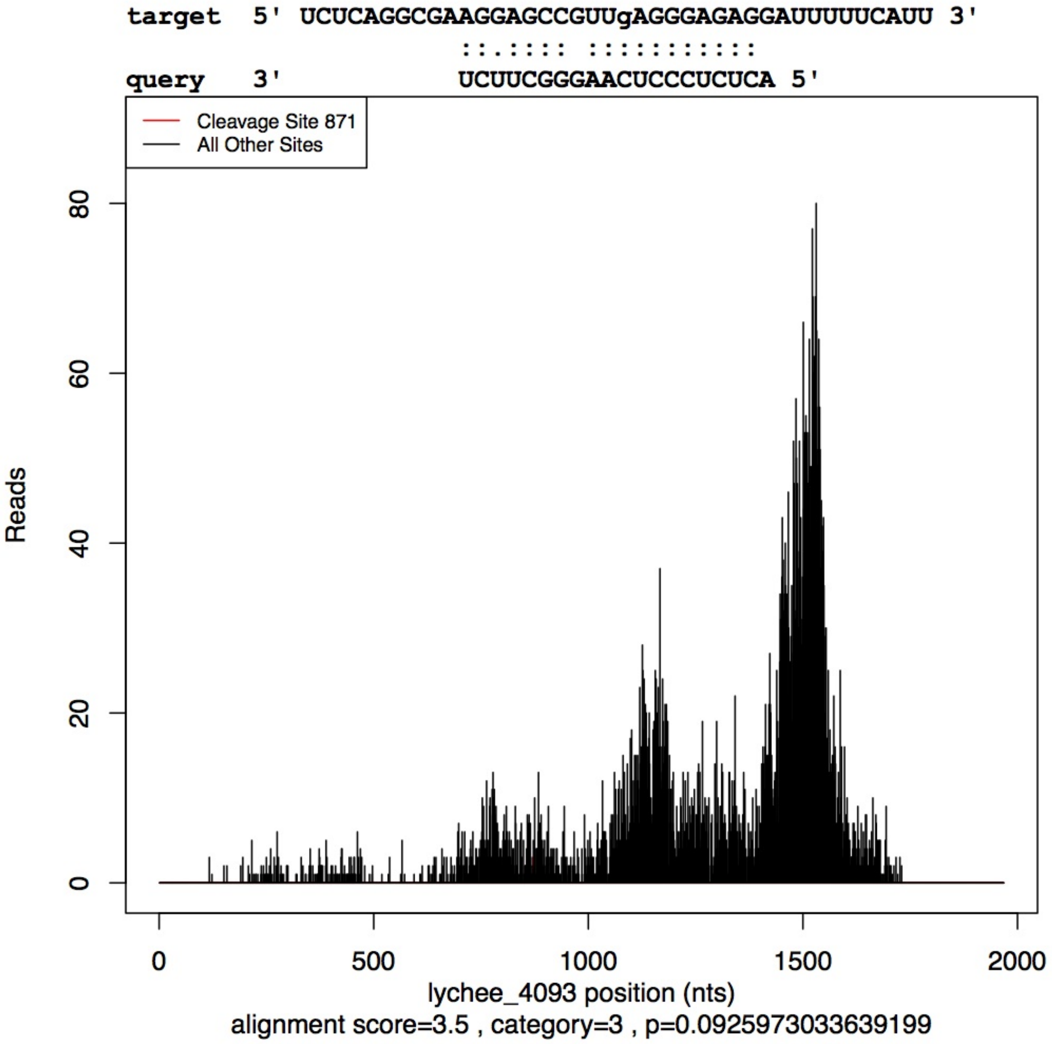

238_miR477_871_lychee_4093

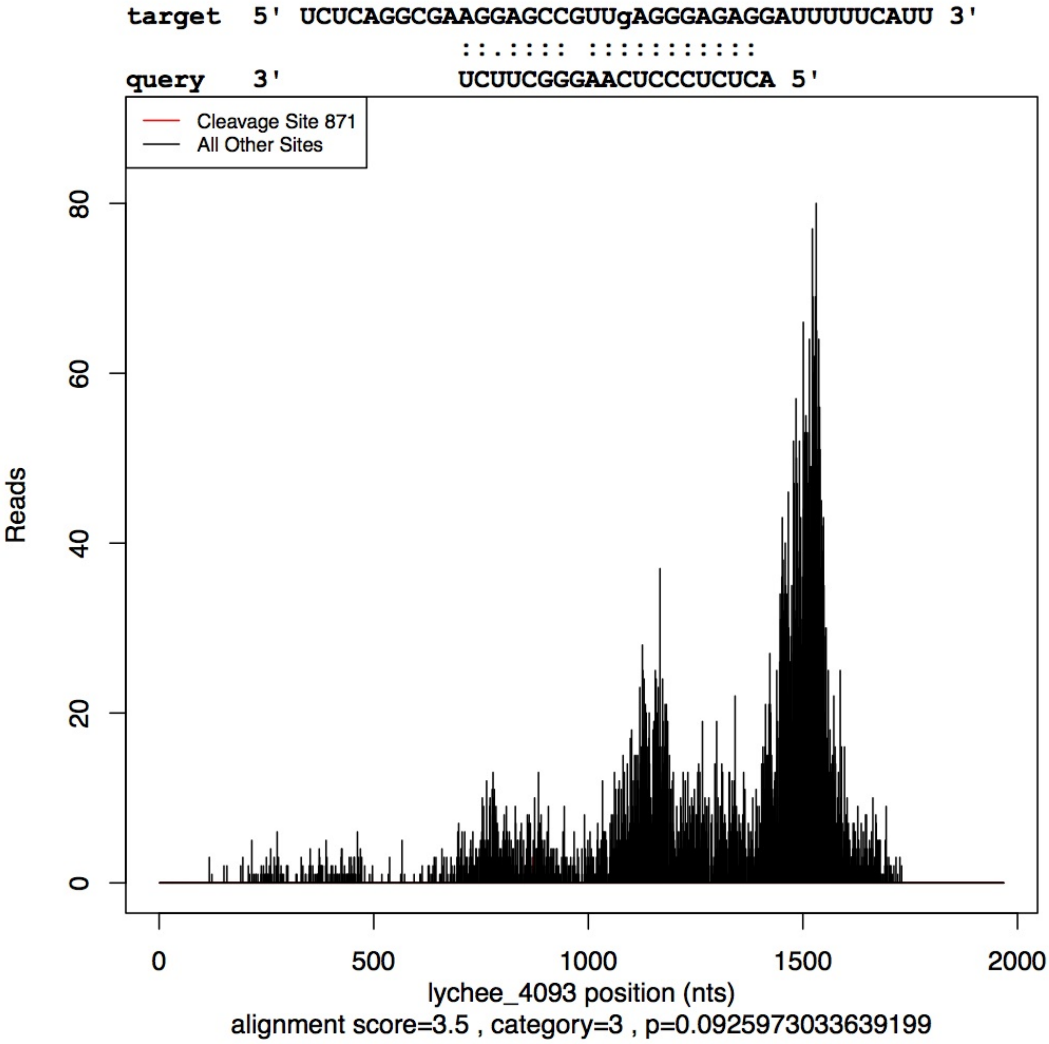

247_miR477_871_lychee_4093

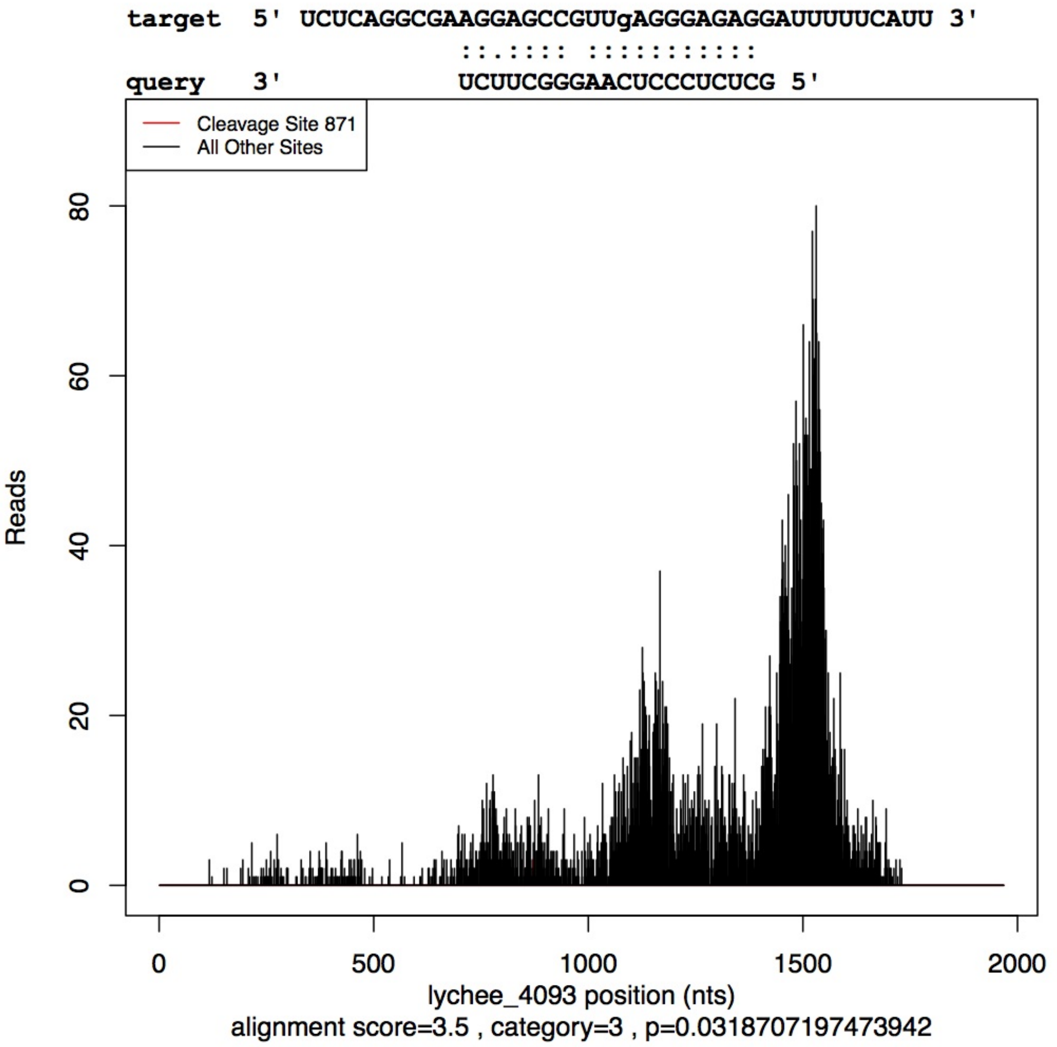

250_miR477_869_lychee_4093

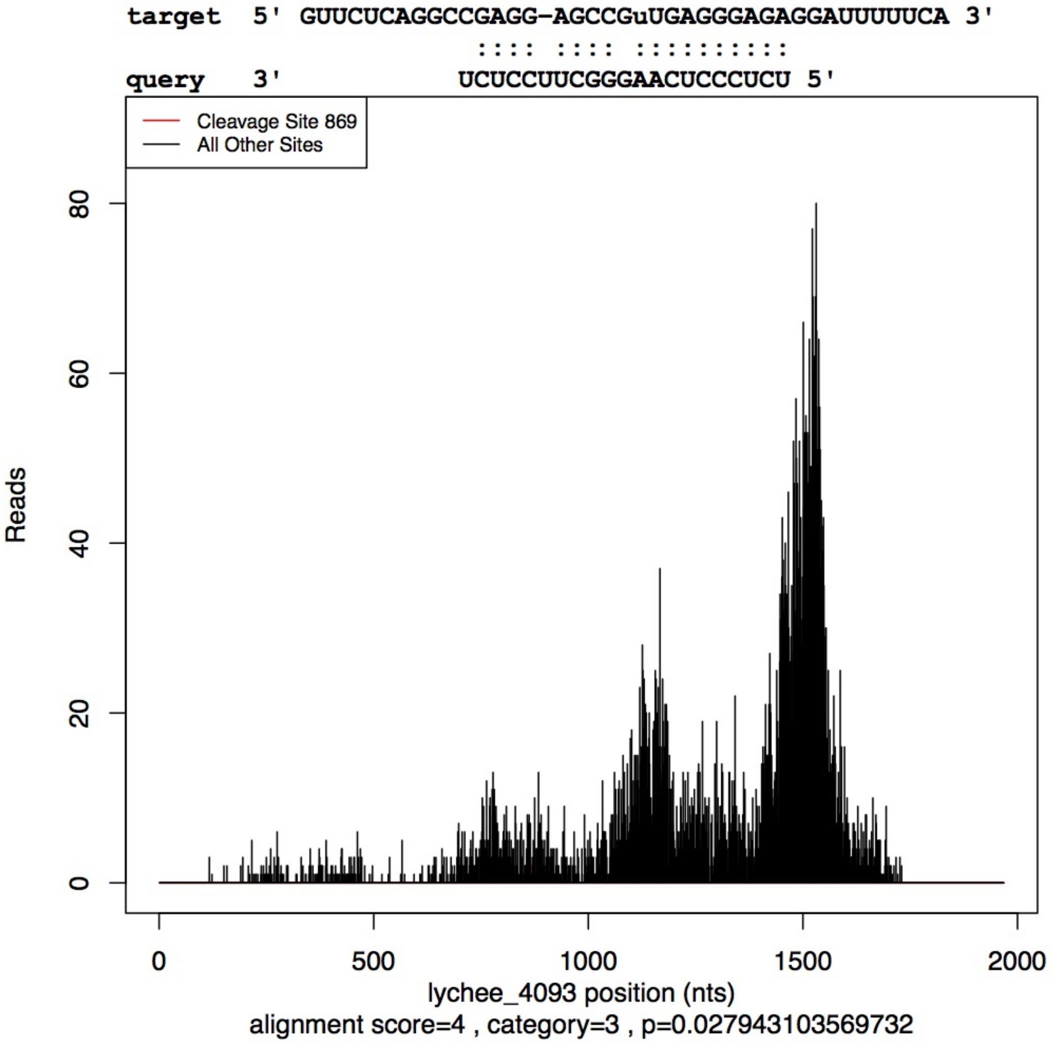

274_miR858_185_lychee_18326

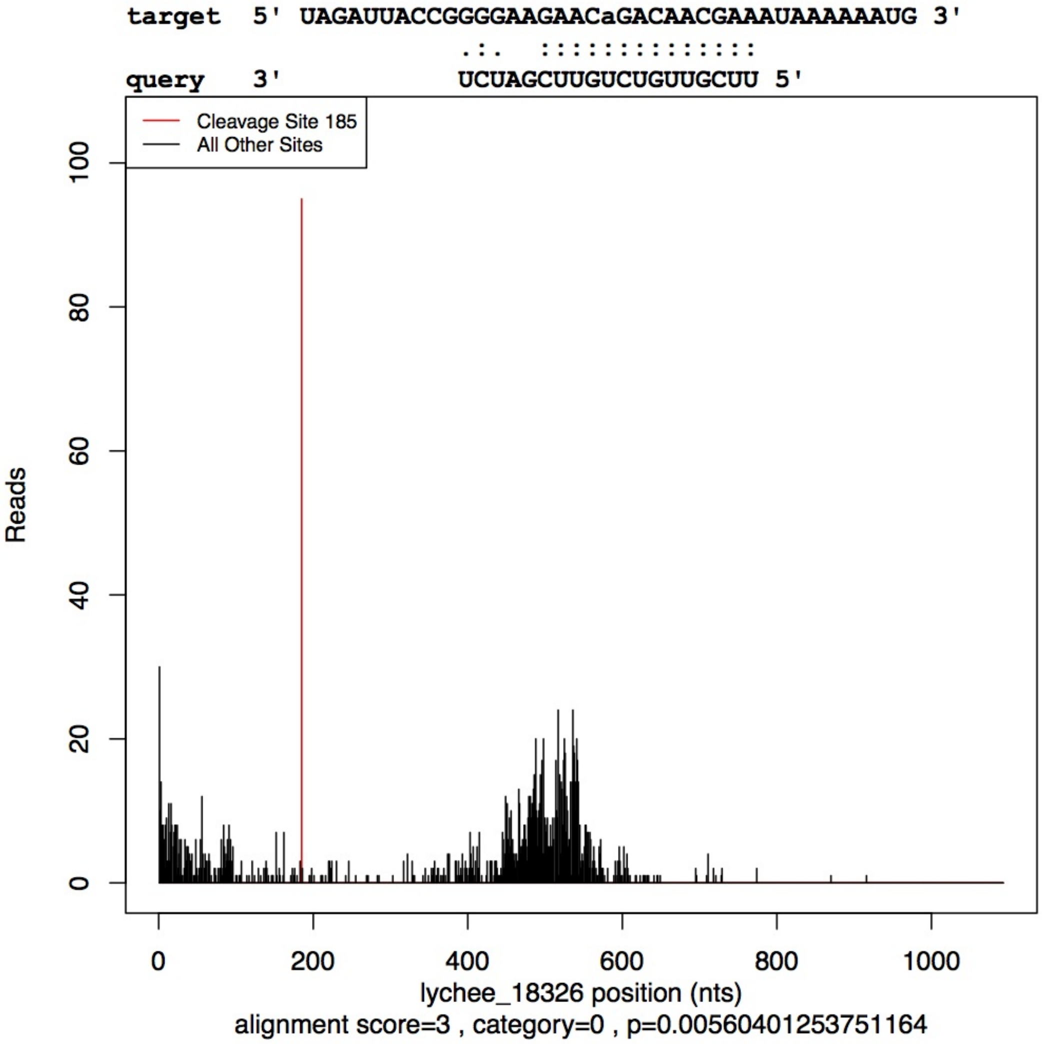

274_miR858_356_lychee_40412

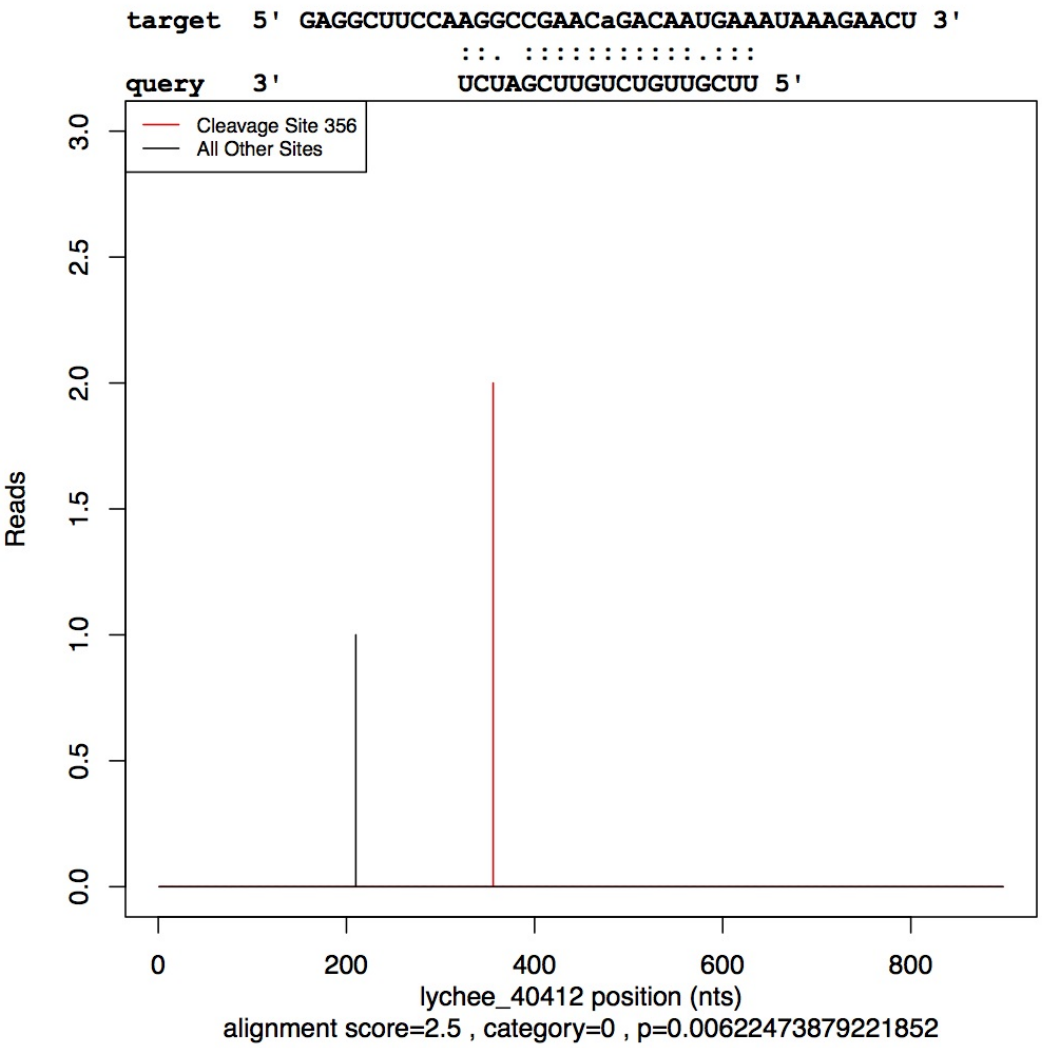

276_miR858_185_lychee_18326

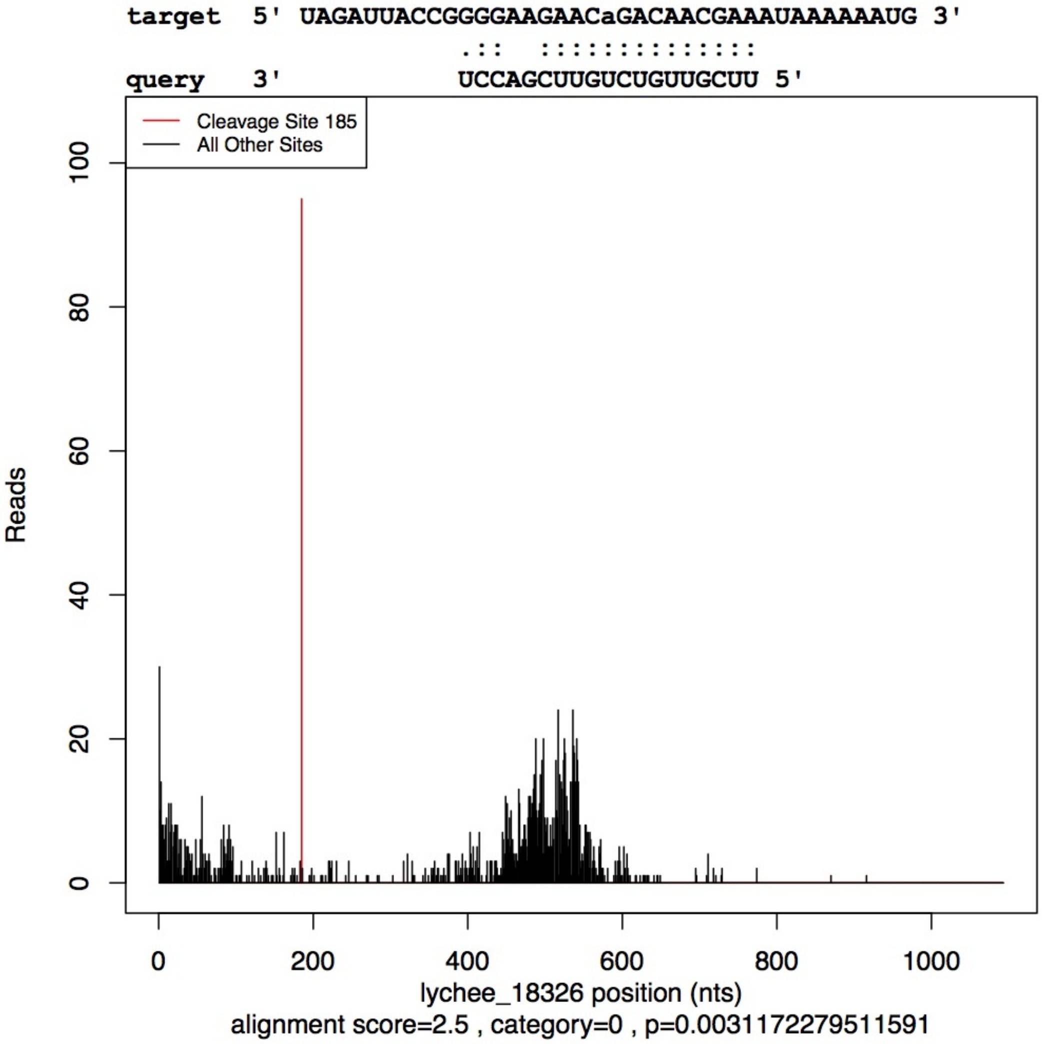

276_miR858_356_lychee_40412

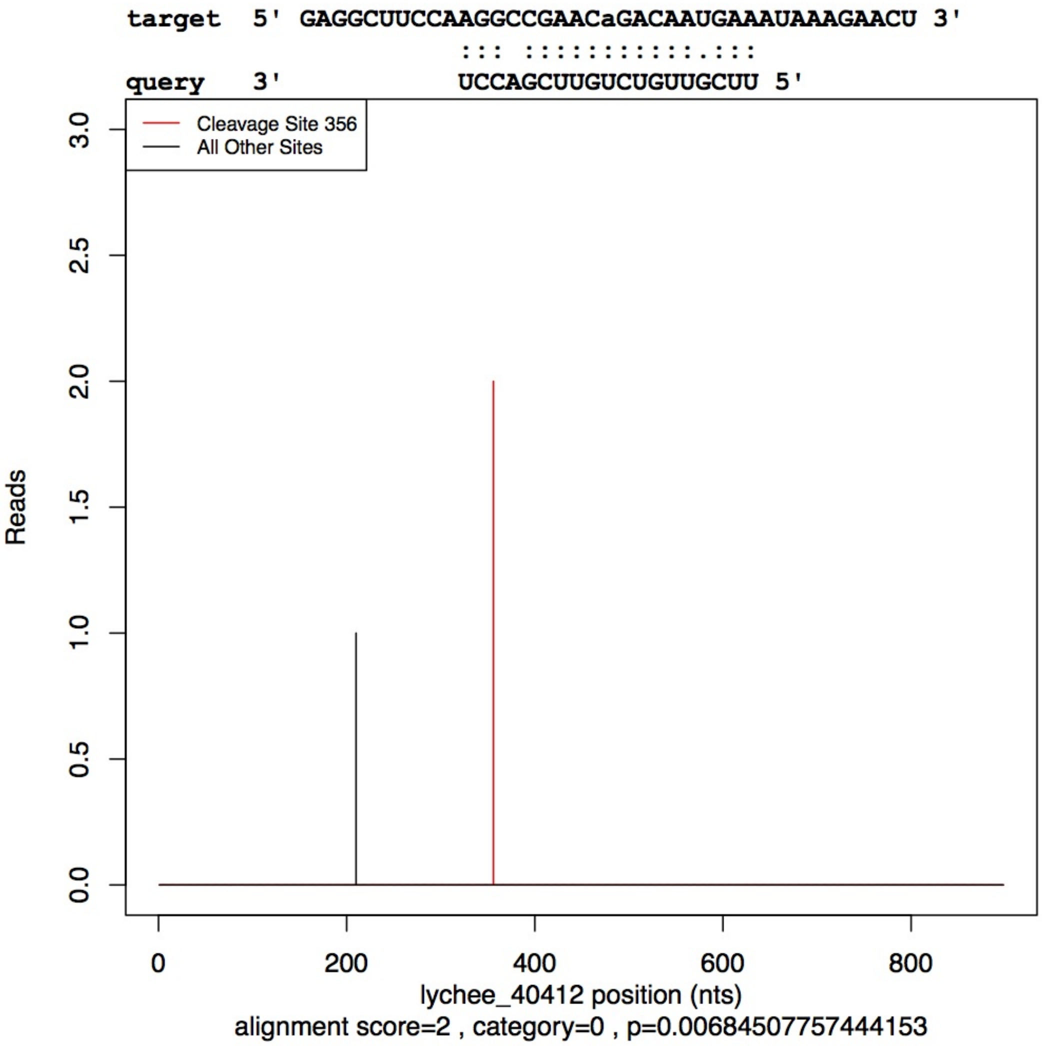

276_miR858_475_lychee_30086

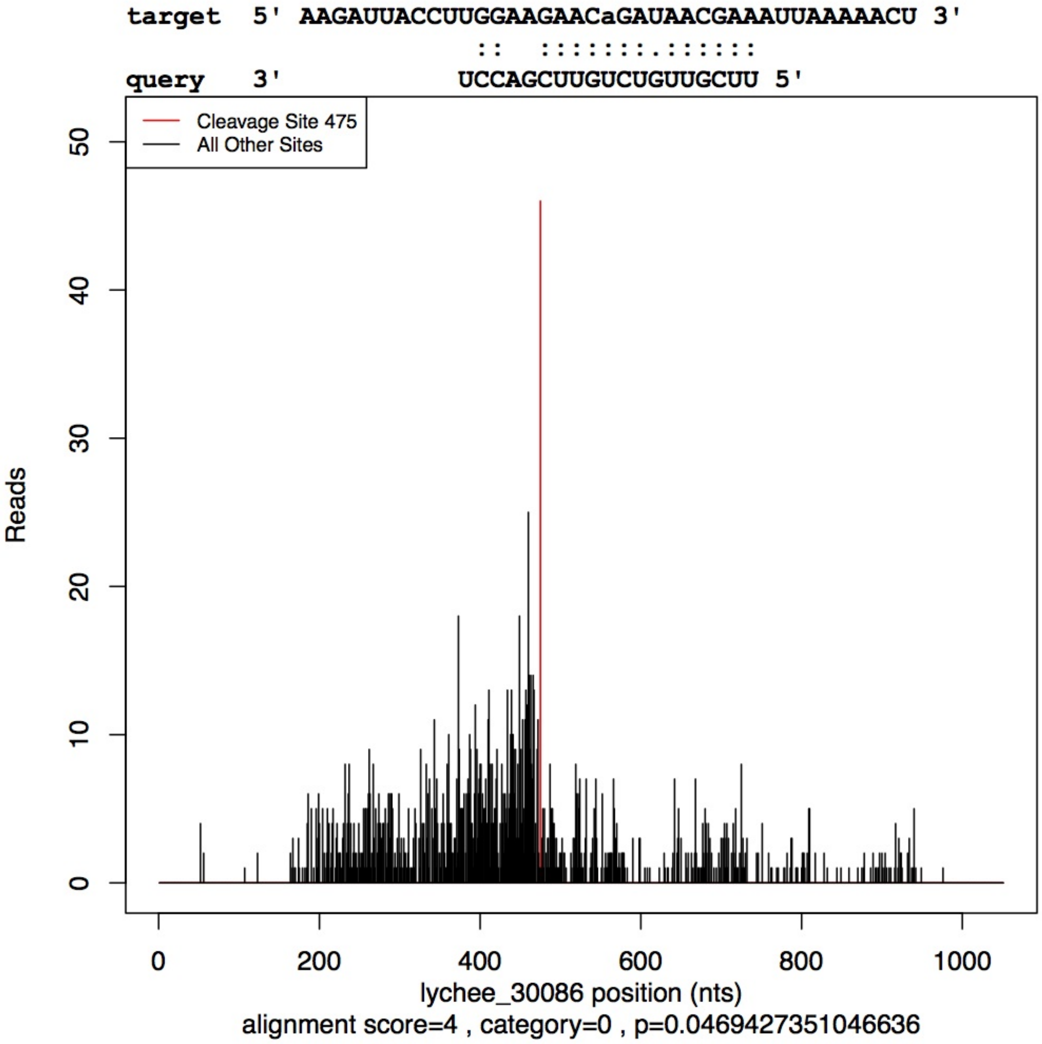

277_miR858_185_lychee_18326

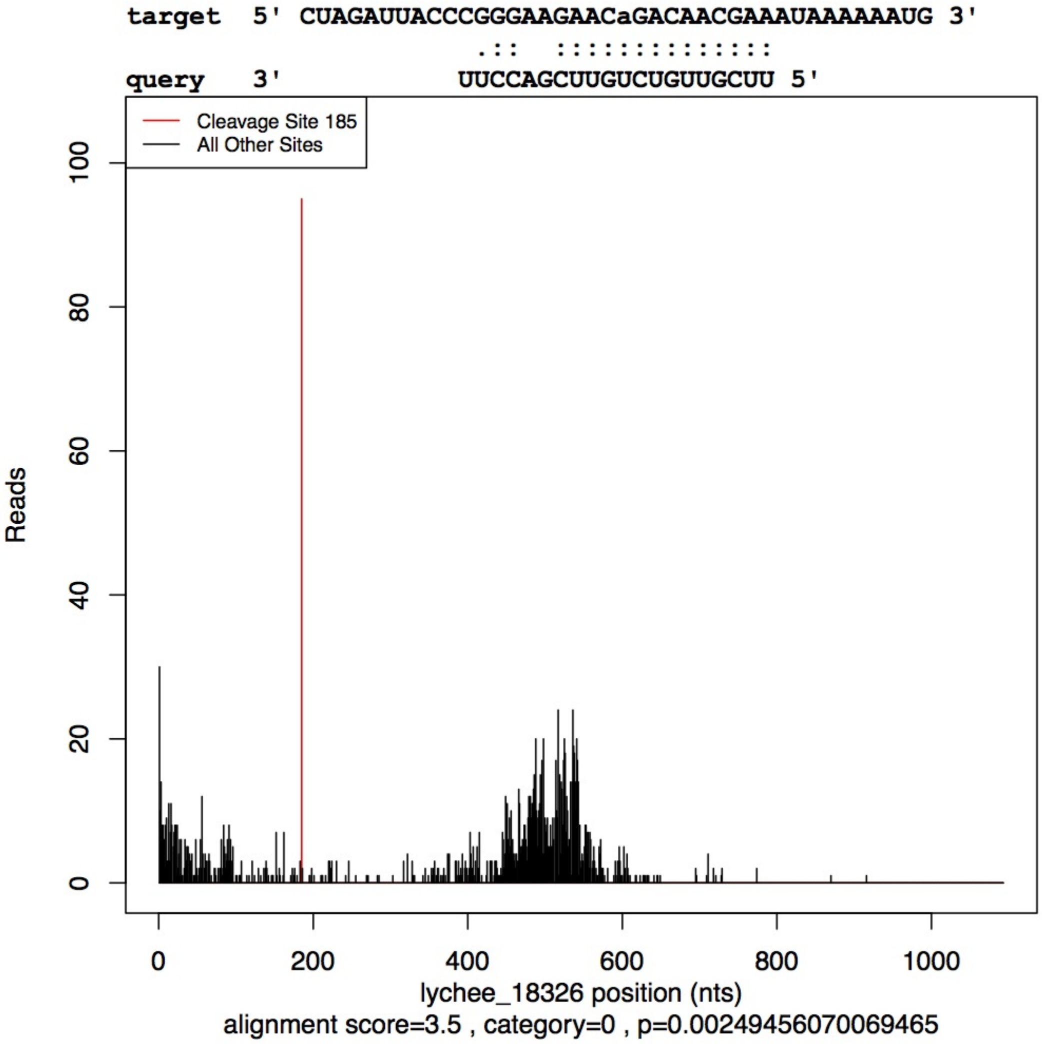

277_miR858_356_lychee_40412

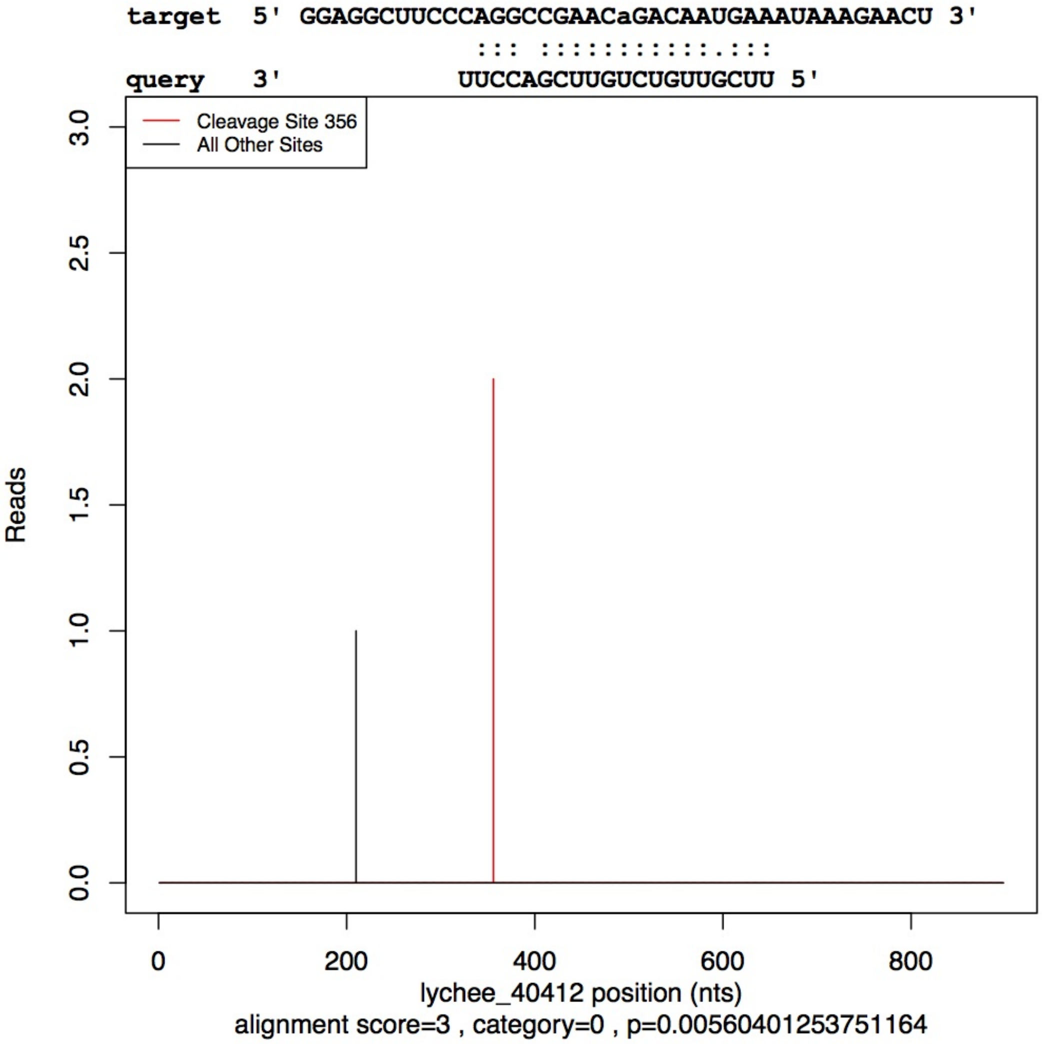

lch-miRC6_1342_lychee_57528

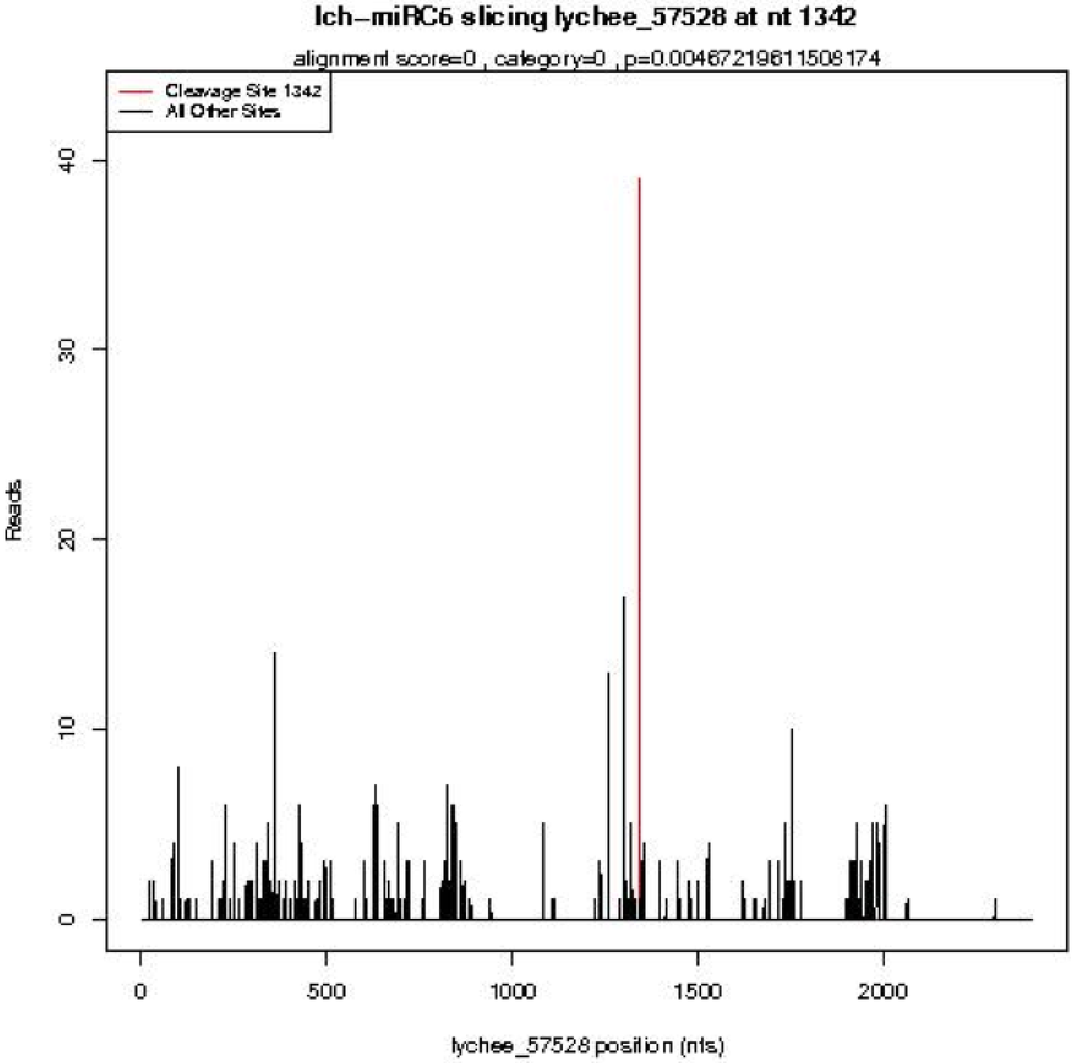

Supplement: Additional file 6: — T-plots for all targets of litchi miRNAs. This file contains t-plots for all targets of litchi miRNAs. Signature abundance throughout the length of the indicated lychee unigene transcripts is plotted. Red line indicates signatures consistent with miRNA-directed cleavage. miRNA:mRNA alignment is shown on the top. [file 12870_2015_509_MOESM6_ESM.pdf]
