# Supplementary figures and images for: MicroRNAs and targets in senescent litchi fruit during ambient storage and post-cold storage shelf life
Source: BMC Plant Biol. 2015 Jul 16;15:181. doi: 10.1186/s12870-015-0509-2 (PMC4504174; doi:10.1186/s12870-015-0509-2)

Figure S3. GO and KEGG functional classification for litchi miRNA targets.

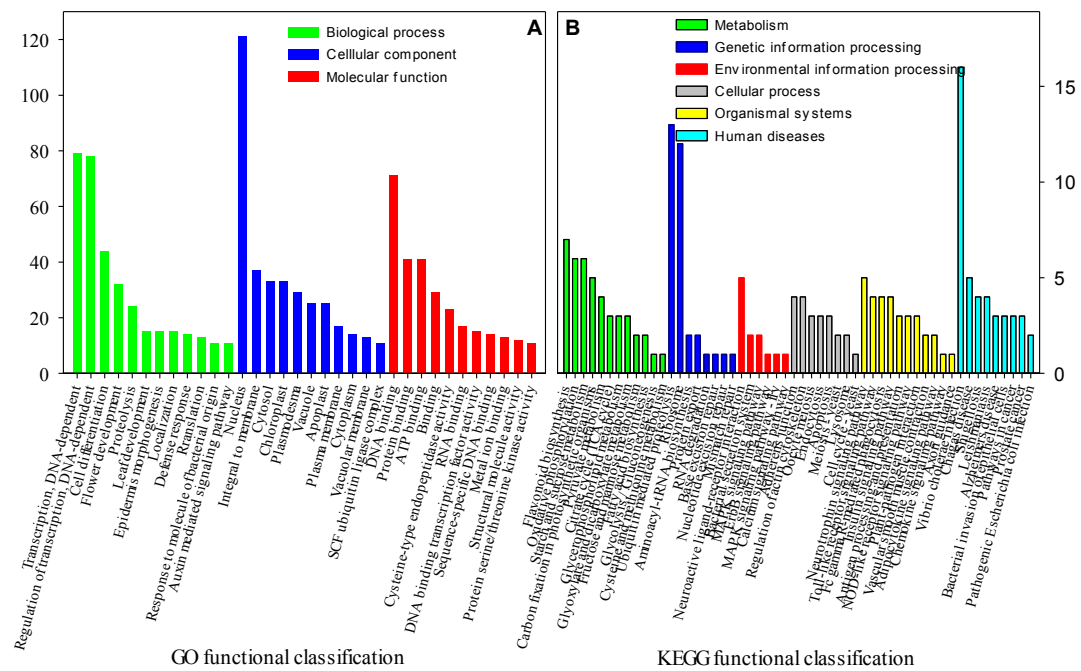

Supplement: Additional file 7: — GO and KEGG functional classification for litchi miRNA targets. This file contains gene functional enrichment analysis for both GO and KEGG pathways. The numbers of genes in the enriched functional classes are presented in the differently colored bars. [file 12870_2015_509_MOESM7_ESM.pdf]

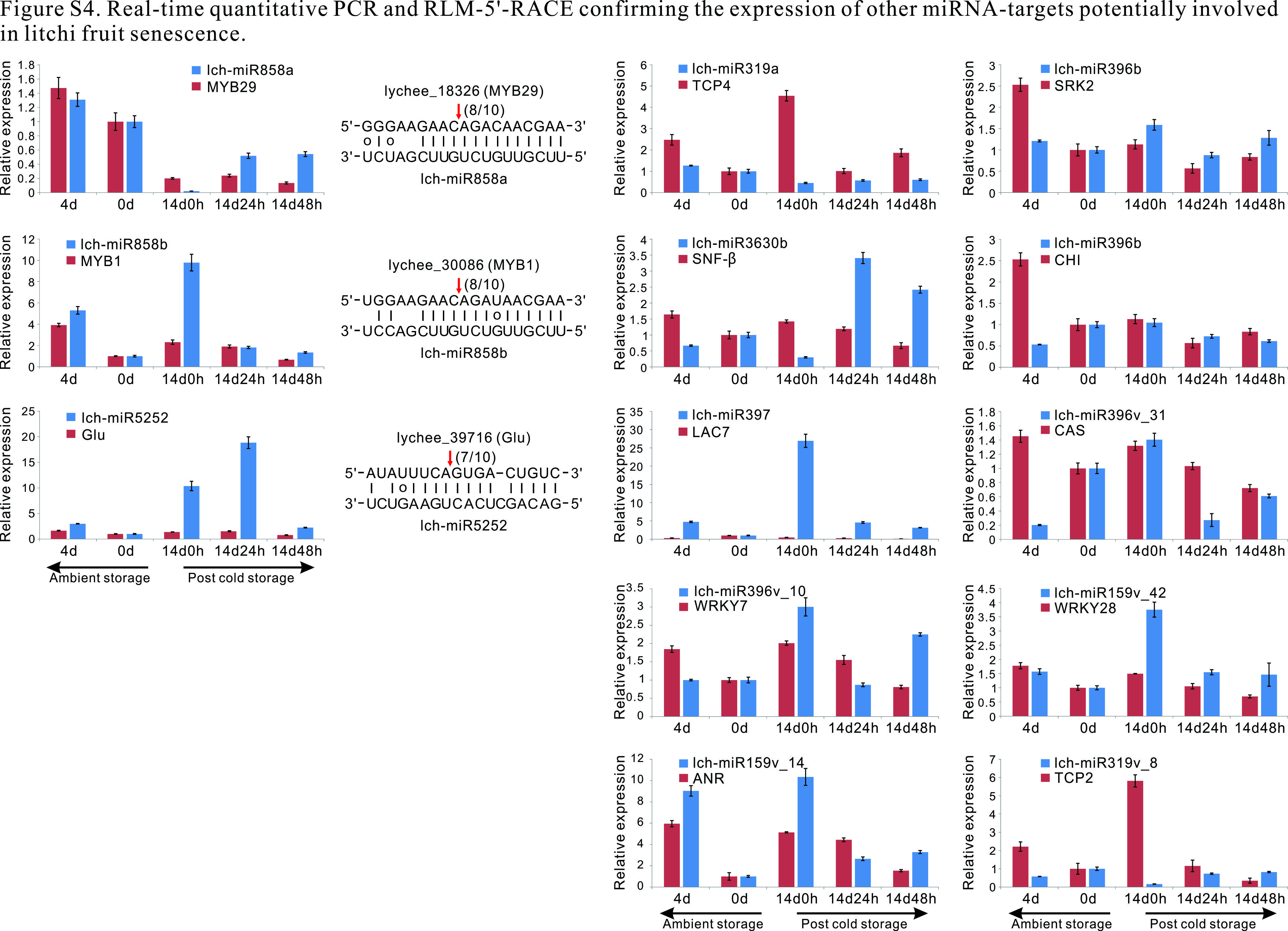

Supplement: Additional file 10: — Real-time quantitative PCR and RLM-5′-RACE confirming the expression of other miRNA-targets potentially involved in litchi fruit senescence. This file contains both qPCR and 5′-RACE validations of selected litchi miRNA-target pairs. Relative expression is normalized to actin, and the normalized miRNA expression at day 0 is arbitrarily set to 1. The data are the mean ± standard deviation (n = 3). The number next to the red arrow above the miRNA:mRNA alignment indicates the number of sequences found at the exact cleavage site. [file 12870_2015_509_MOESM10_ESM.jpeg]
